# A Universal Method for the Synthesis of new Heterocyclic Systems: Pyrimido[2,1‐*f*][1,2,4]triazines

**DOI:** 10.1002/open.202400379

**Published:** 2025-01-31

**Authors:** Samvel N. Sirakanyan, Domenico Spinelli, Athina Geronikaki, Victor G. Kartsev, Elmira K. Hakobyan, Hasmik V. Jughetsyan, Hasmik A. Yegoryan, Anush A. Hovakimyan

**Affiliations:** ^1^ Scientific Technological Center of Organic and Pharmaceutical Chemistry of National Academy of Science of Republic of Armenia Institute of Fine Organic Chemistry of A. L. Mnjoyan Armenia 0014 Yerevan, Ave. Azatutyan 26; ^2^ Dipartimento di Chimica G. Ciamician Alma Mater Studiorum-Università di Bologna Via F. Selmi 2 Bologna 40126 Italy; ^3^ Aristotle University of Thessaloniki School of Pharmacy Thessaloniki 54124 Greece; ^4^ InterBioScreen Moscow 119019 Russia

**Keywords:** acylation, aminomethyl derivatives, cyclization, hydrazinolysis, pyrimido[2,1-*f*][1,2,4]triazine

## Abstract

The synthesis of pyrimido[2,1‐*f]*[1,2,4]triazines was performed in four steps. Compounds obtained by acylation of the starting amino esters of thieno[2,3‐*b*]pyridines were reacted with various amines. The resulting amino derivatives underwent cyclization in the presence of hydrazine hydrate leading to new aminomethyl derivatives of thieno[3,2‐*d*]pyrimidines. Further cyclization of the latter resulted to the synthesis of new unique heterocyclic systems: pyrido[3′′,2′′:4′,5′]thieno[3′,2′:4,5]pyrimido[2,1‐*f*][1,2,4]triazines. The uniqueness of these systems lies in the fact that even the combination of the last two cycles represents a new heterocyclic system.

## Graphical abstract

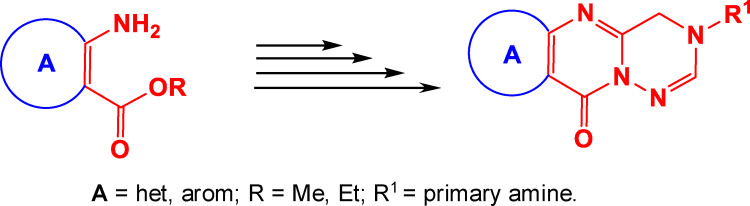

## Introduction

**Figure 1:**
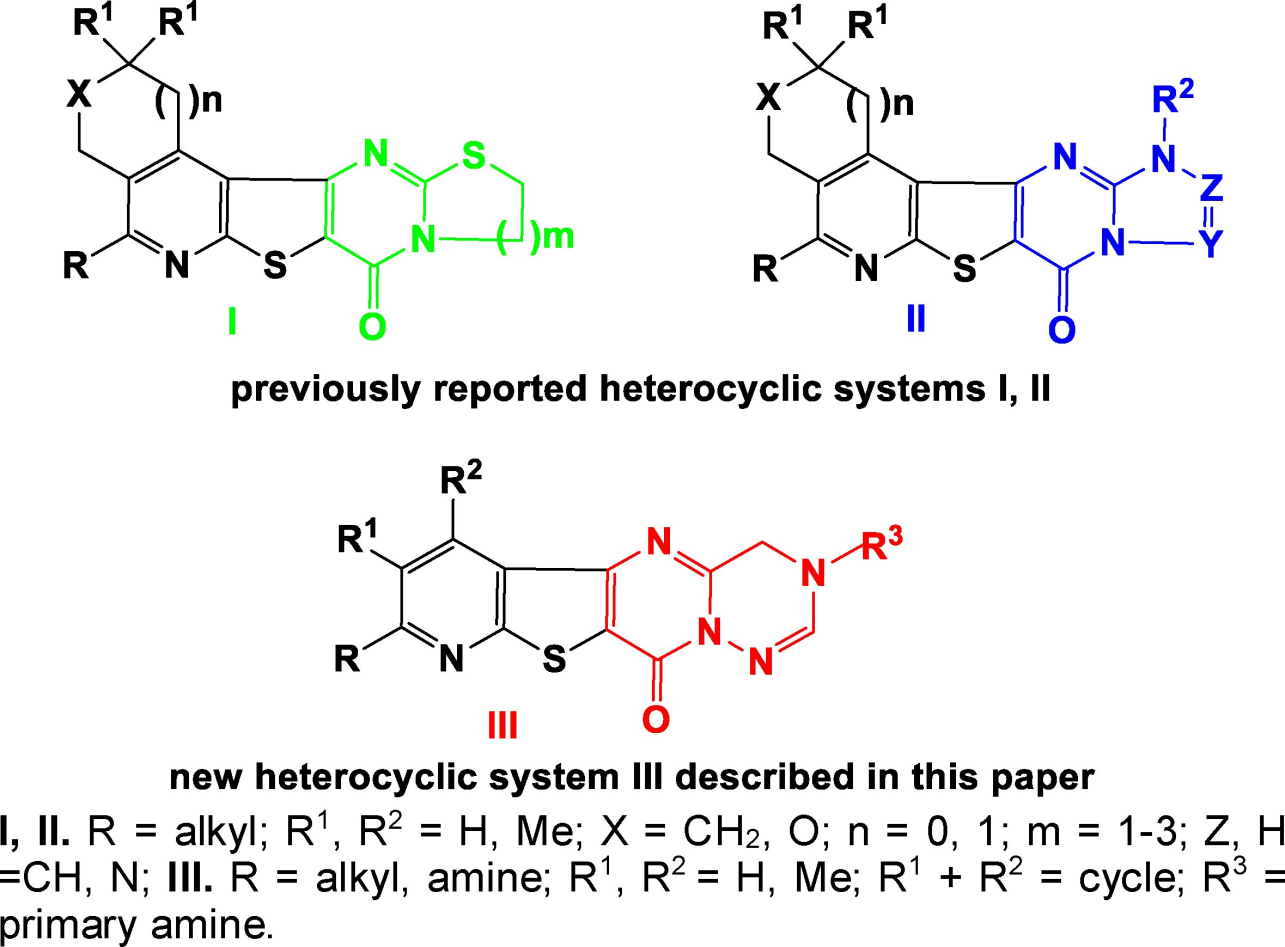
The general structures of previously **I, II** and new **III** synthesized compounds.

Heterocyclic compounds attract an interest of scientists^[1]^ especially medicinal chemists due to their wide range of different pharmacological activities. Among them, many derivatives of condensed thieno[3,2‐*d*]pyrimidines, exhibiting high biological activity[^2^, ^3^, ^4^, ^5^, ^6^, ^7^, ^8^, ^9^, ^10^, ^11^, ^12^] can be distinguished. In particular, studies have shown that these compounds possess antimicrobial,[^13^, ^14^, ^15^] antitumor,[^16^, ^17^, ^18^, ^19^, ^20^] cytotoxic^[21]^ and antiproliferative[^22^, ^23^] activities. Previously, we have also reported on the synthesis and biological activity of some derivatives of fused thieno[3,2‐*d*]pyrimidines.[^24^, ^25^, ^26^, ^27^, ^28^, ^29^, ^30^, ^31^] The above observation prompted us to synthesize new 3‐substituted derivatives of fused pyrimidines with suitable functional group, followed by the synthesis of new heterocyclic systems, namely pyrimido[2,1‐*f*][1,2,4]triazines. It should be noted that obtained new heterocyclic systems are open new directions in the field of fused heterocycles. The derivatives of such fused pentacyclic systems that we synthesized are distinguished by different biological activities. In particular, thiazolo[3,2‐*a*]pyrimidines, pyrimido[2,1‐*b*][1,3]thiazines **I** and triazolopyrimidines **II** have shown high antimicrobial activity (Figure 1).[^26^, ^27^, ^30^] Considering the significance of heterocyclic compounds, it is highly beneficial to develop universal techniques for synthesizing a wide range of new heterocyclic compounds. This article discusses a technique that is intriguing from both a practical and theoretical perspective.

## Results and Discussion

**Scheme 1:**
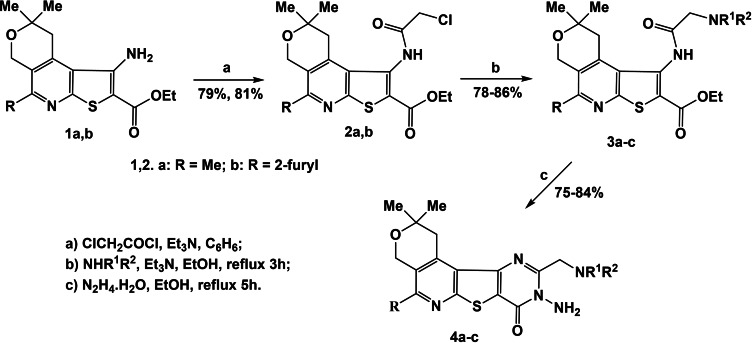
The synthesis of new 3‐substituted derivatives of fused pyrimidines **4 a–c**.

Our preliminary goal was the synthesis of 3‐substituted fused pyrimidine derivatives with four cycles **4** based on available suitable functional groups of amino esters **1a,b**.^[30]^ By treating amino esters of thieno[2,3‐b]pyridines **1a,b** with chloroacetyl chloride followed by substitution of the active chlorine atom with various amines, a novel series of amino derivatives of thieno[2,3‐*b*]pyridines **3a–c** was synthesized. Compounds **3 a–c** undergo cyclization in ethanol under the action of hydrazine hydrate^[32]^ leading to aminomethyl derivatives of thieno[3,2‐*d*]pyrimidines **4 a–c** (Scheme 1, Table 1).

**Table 1:** Thieno[2,3‐*b*]pyridines **3 a–c** and thieno[3,2‐*d*]pyrimidines **4 a–c**.

N	Compound	N	Compound
3a	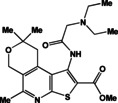	4a	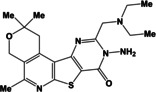
3b	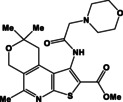	4b	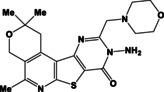
3c	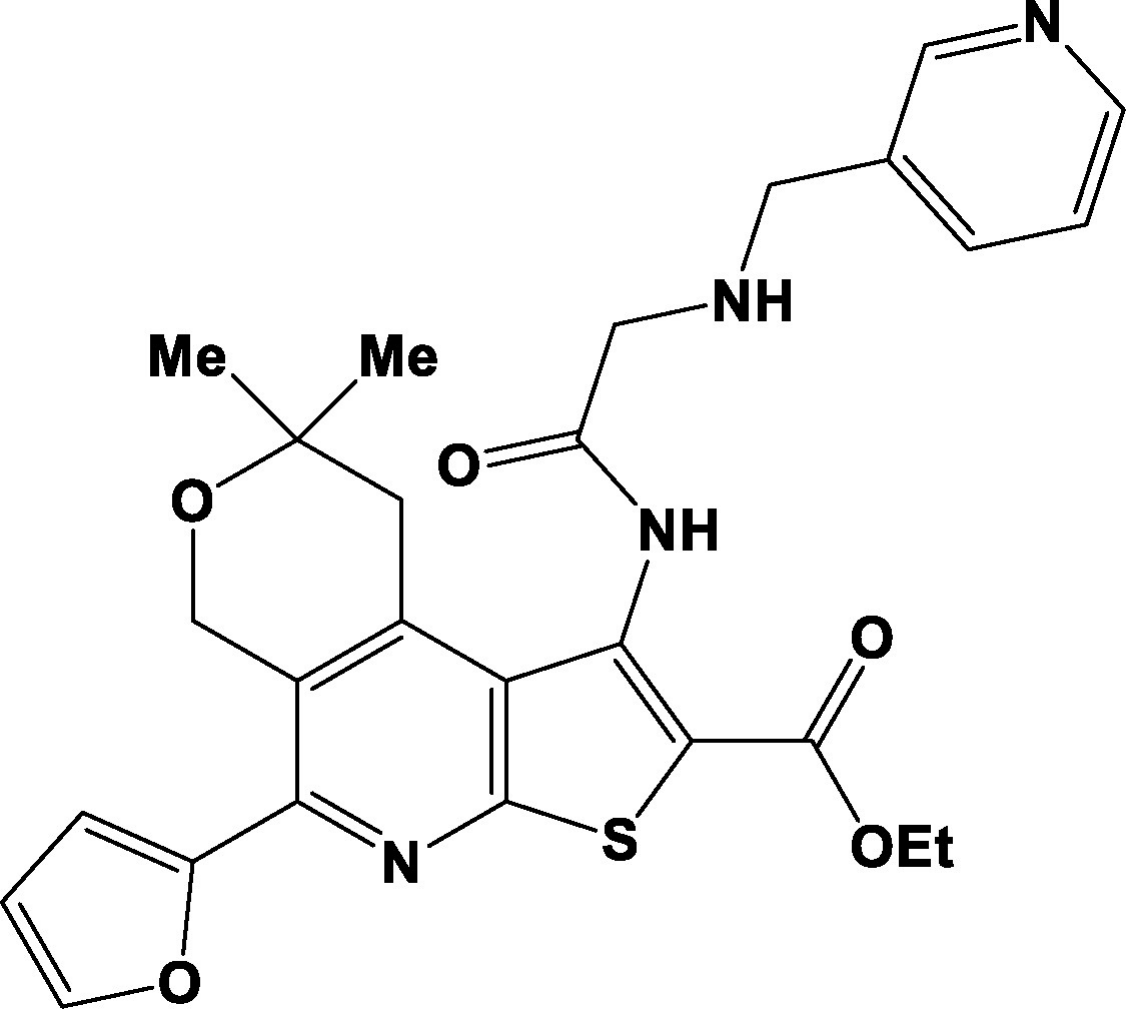	4c	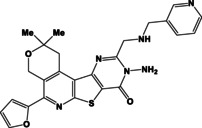

**Scheme 2:**
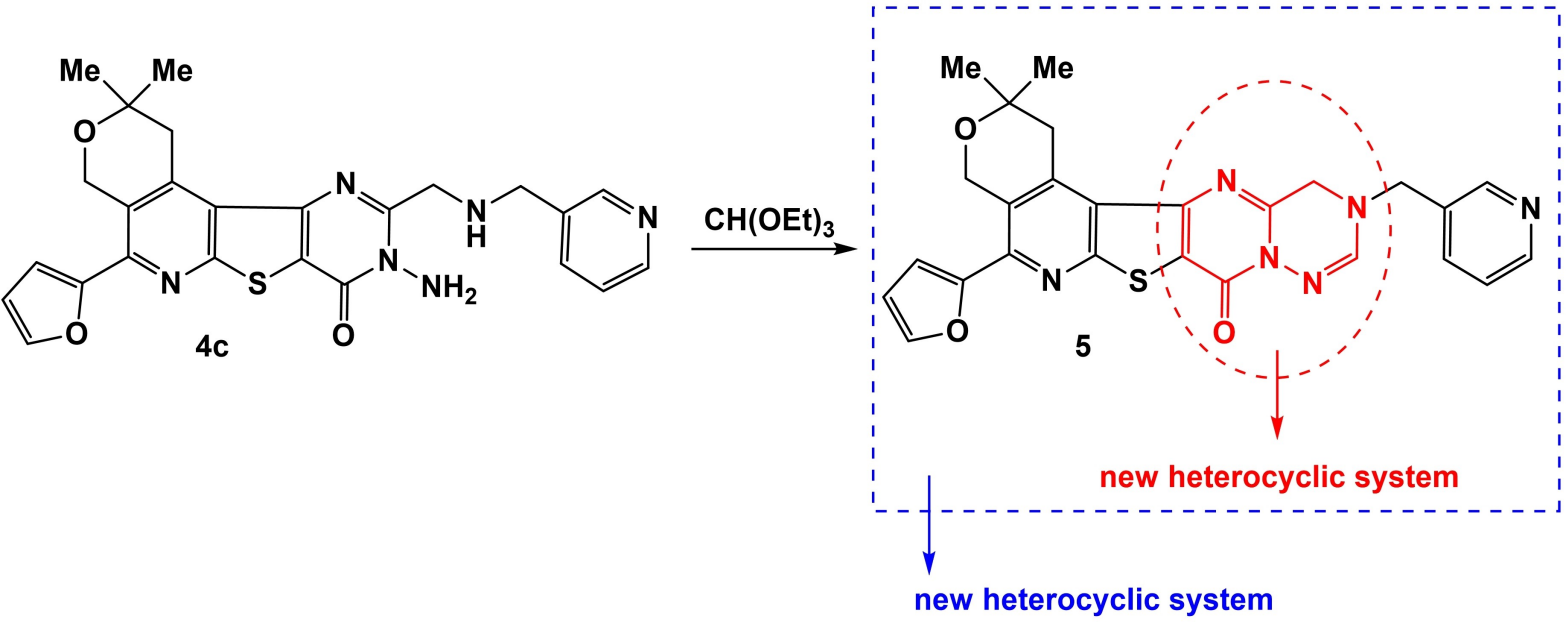
Synthesis of new heterocyclic system: 5‐(2‐furyl)‐2,2‐dimethyl‐12‐(pyridin‐3‐ylmethyl)‐1,4,12,13‐tetrahydro‐2*H*,8*H*‐pyrano[4′′′,3′′:4′′,5′′]pyrido[3′′,2′′:4′,5′]thieno[3′,2′:4,5]pyrimido[2,1‐*f*][1,2,4]triazin‐8‐one **5**.

The presence of NH group in the 3^rd^ position of the pyrimidine ring in compound **4c**, enabled a cyclization reaction with triethyl orthoformate to form a new heterocyclic system with 5 rings: 5‐(2‐furyl)‐2,2‐dimethyl‐12‐(pyridin‐3‐ylmethyl)‐1,4,12,13‐tetrahydro‐2*H*,8*H*‐pyrano[4′′′,3′′:4′′,5′′]pyrido[3′′,2′′:4′,5′]thieno[3′,2′:4,5]pyrimido[2,1‐*f*][1,2,4]triazin‐8‐one **5**. The resulting heterocyclic system appeared to be new, unique and differs from the pentacyclic heterocyclic systems previously obtained by our group. The uniqueness of compound **5** is attributed to the fact that, in addition to the first three main cycles (pyrano[4,3‐*d*]thieno[2,3‐*b*]pyridine), the combination of the last two cycles (pyrimido[2,1‐*f*][1,2,4]triazine) by itself represents a new heterocyclic system (Scheme 2).

It should be noted that there are two conditions for the implementation of this method:

- the starting compounds must have vicinal amino and ester groups,
- the amine substituted in the 3^rd^ position of the pyrimidine ring (in compounds **4**) must be primary amine.

**Figure 2:**
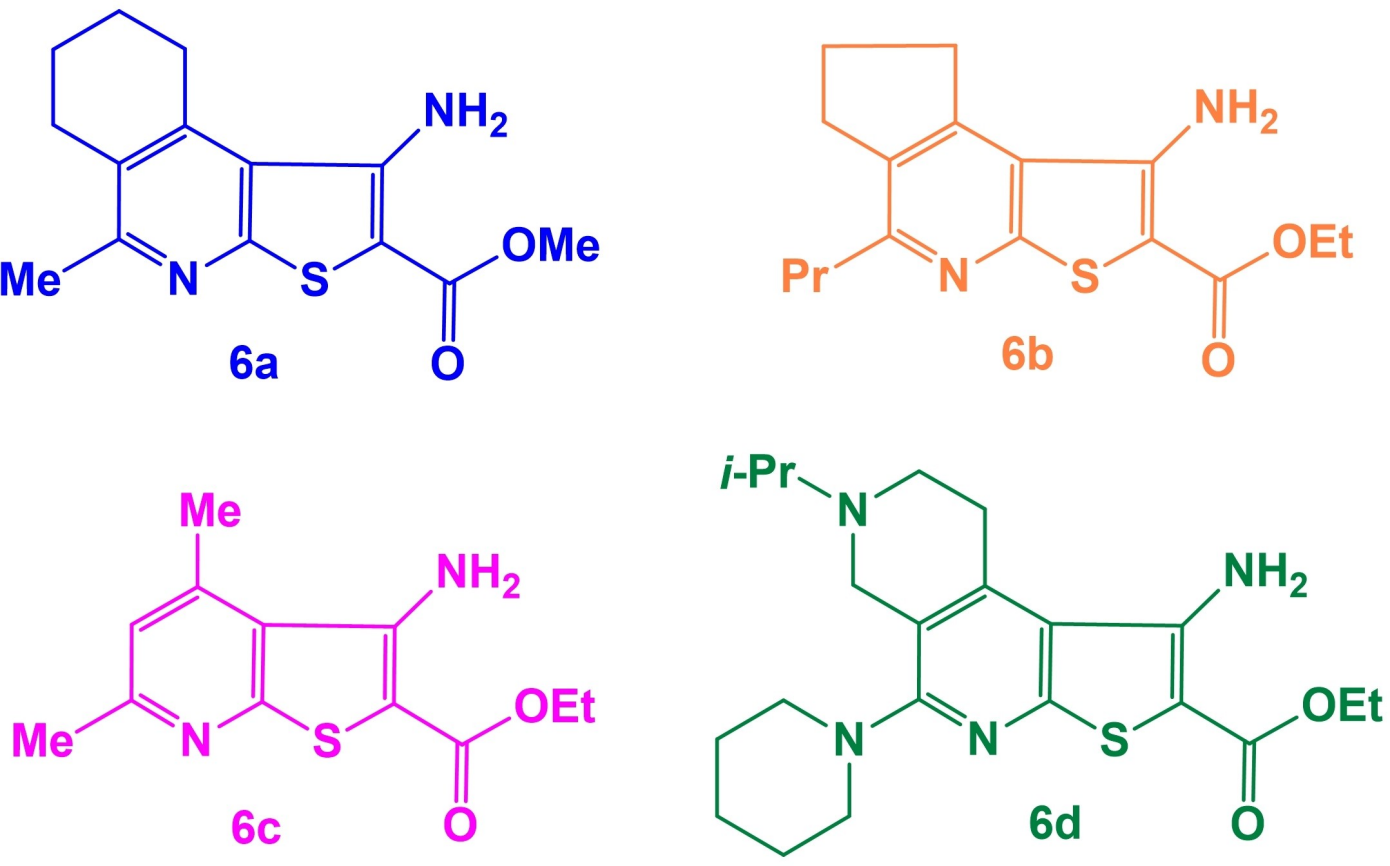
Starting compounds **6a‐d**.

This methodology was subsequently employed for the systems derived from tetrahydroisoquinoline **6a**, cyclopenta[c]pyridine **6b**, dimethylpyridine **6c**, and 2,7‐naphthyridine **6d** (Figure 2).[^31^, ^33^]

**Scheme 3:**
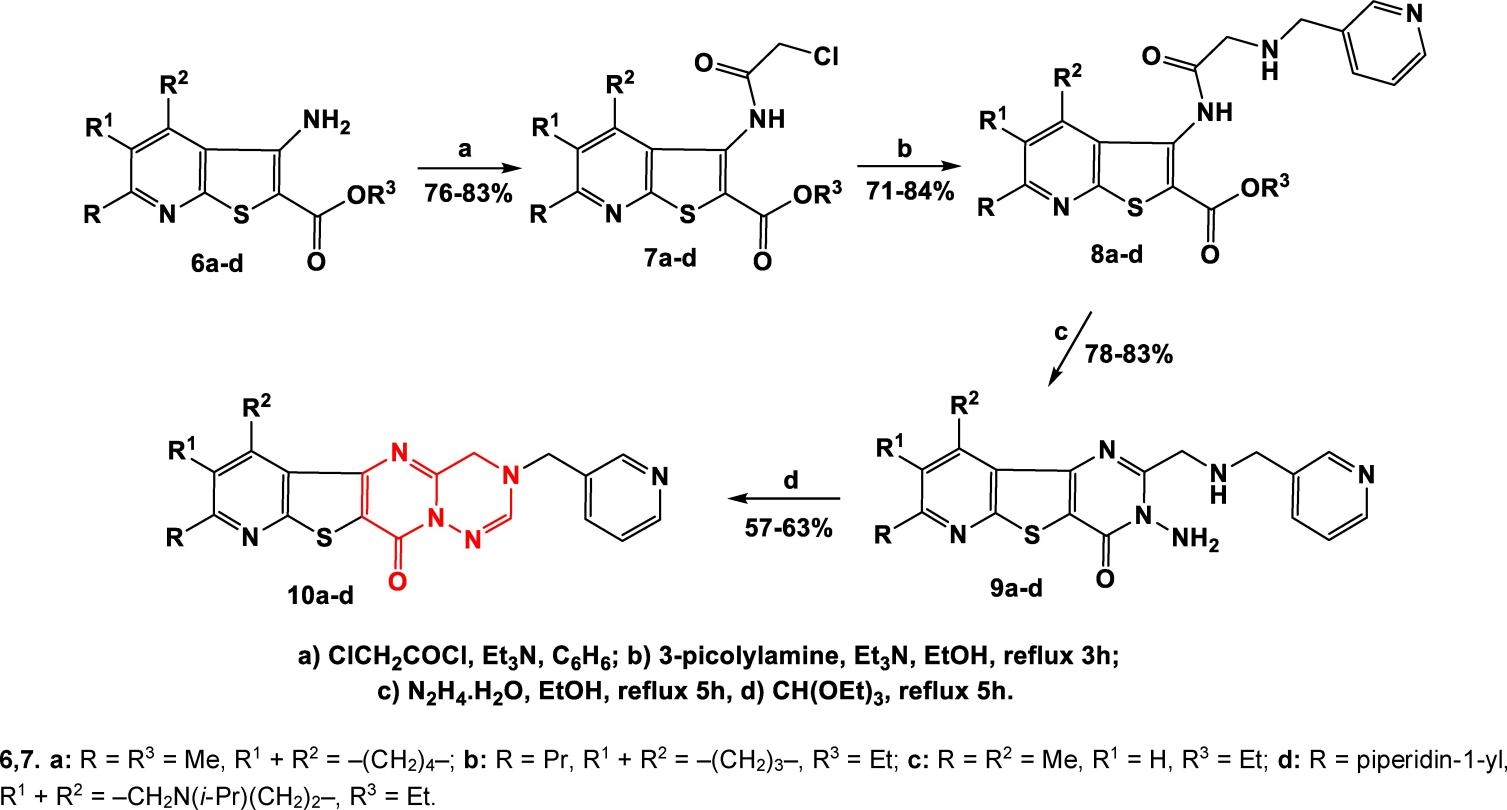
Synthesis of new heterocyclic systems: pyrimido[2,1‐*f*][1,2,4]trazines **10a**–**d**.

The transformations carried out according to Schemes 1 and 2 led to the synthesis of four new heterocyclic systems bearing a pyrimido[2,1‐*f*][1,2,4]triazine ring, which once again proved that the resulting heterocyclic systems are truly unique (Scheme 3, Table 2).

**Table 2:** Thieno[2,3‐*b*]pyridines **8a**–**d**, thieno[3,2‐*d*]pyrimidines **9a**–**d** and pyrimido[2,1‐*f*][1,2,4]triazines **10a**–**d**.

N	Compound**	Yield*(%)	N	Compound**	Yield*(%)
8a	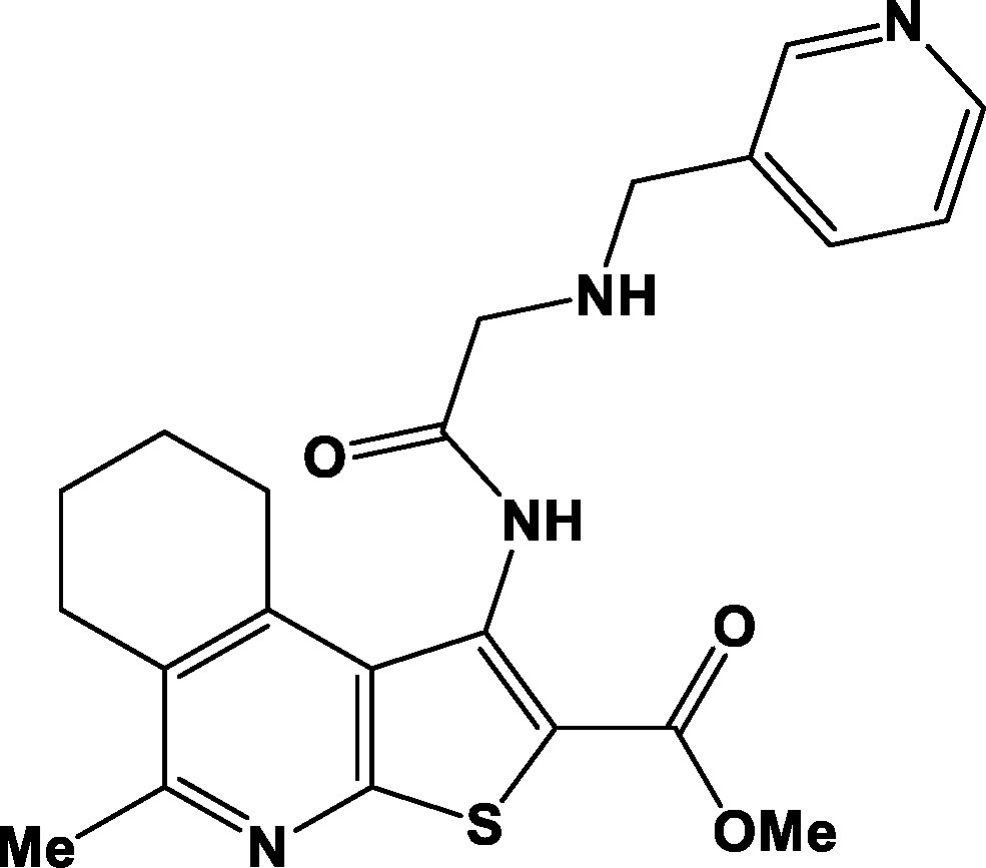	79	9c	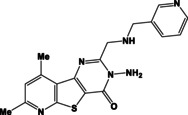	78
8b	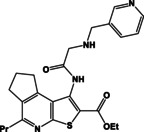	82	9d	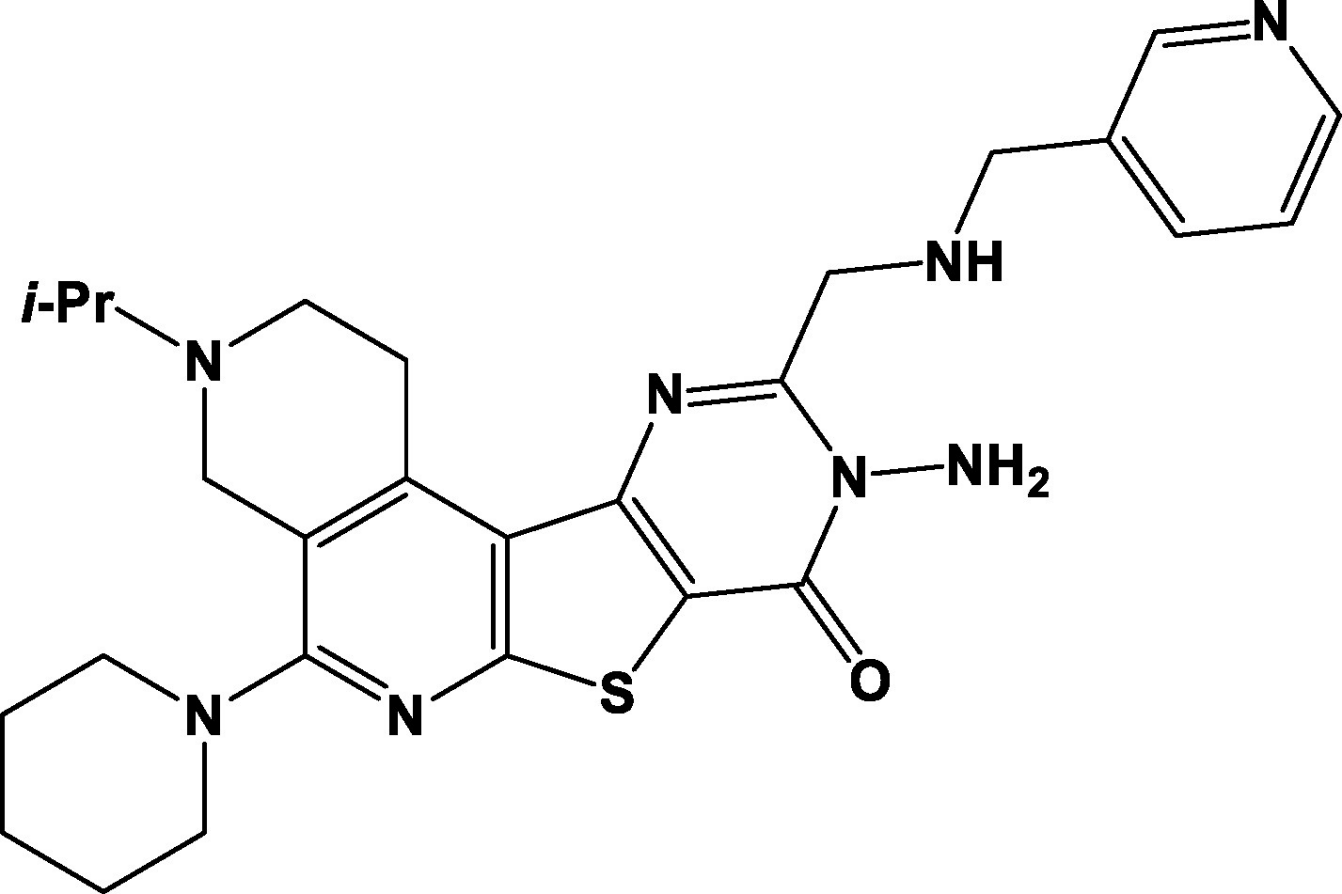	80
8c	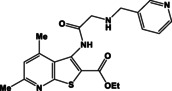	84	10a	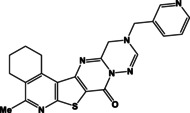	61
8d	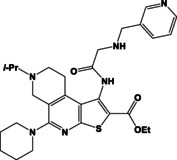	71	10b	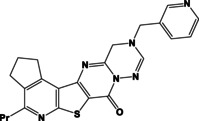	63
9a	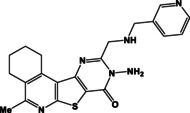	79	10c	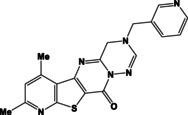	59
9b	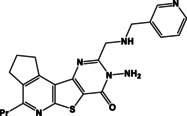	83	10d	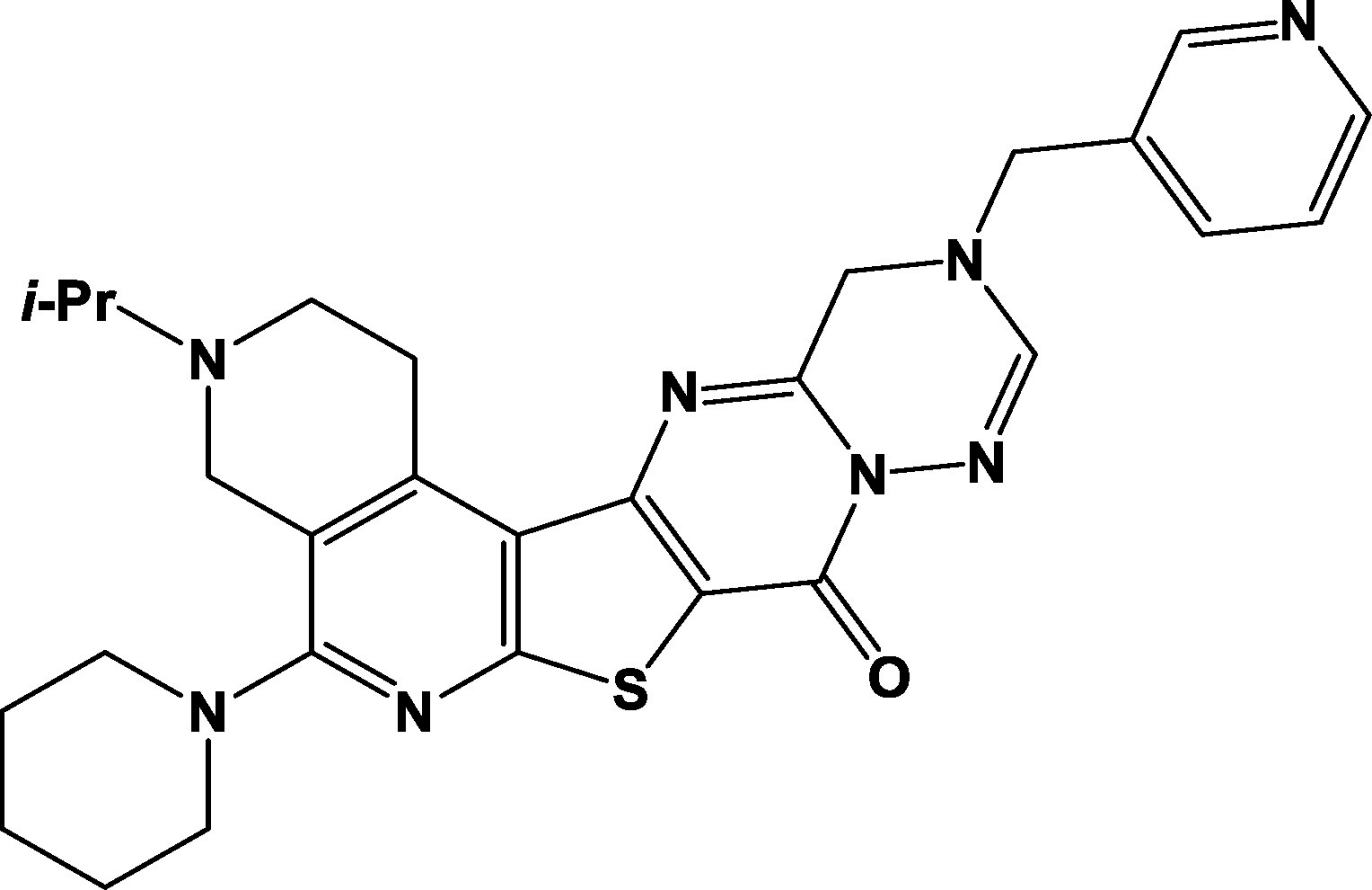	57

*Yields after recrystallization. **Purity of compounds≤95 %.

**Scheme 4:**
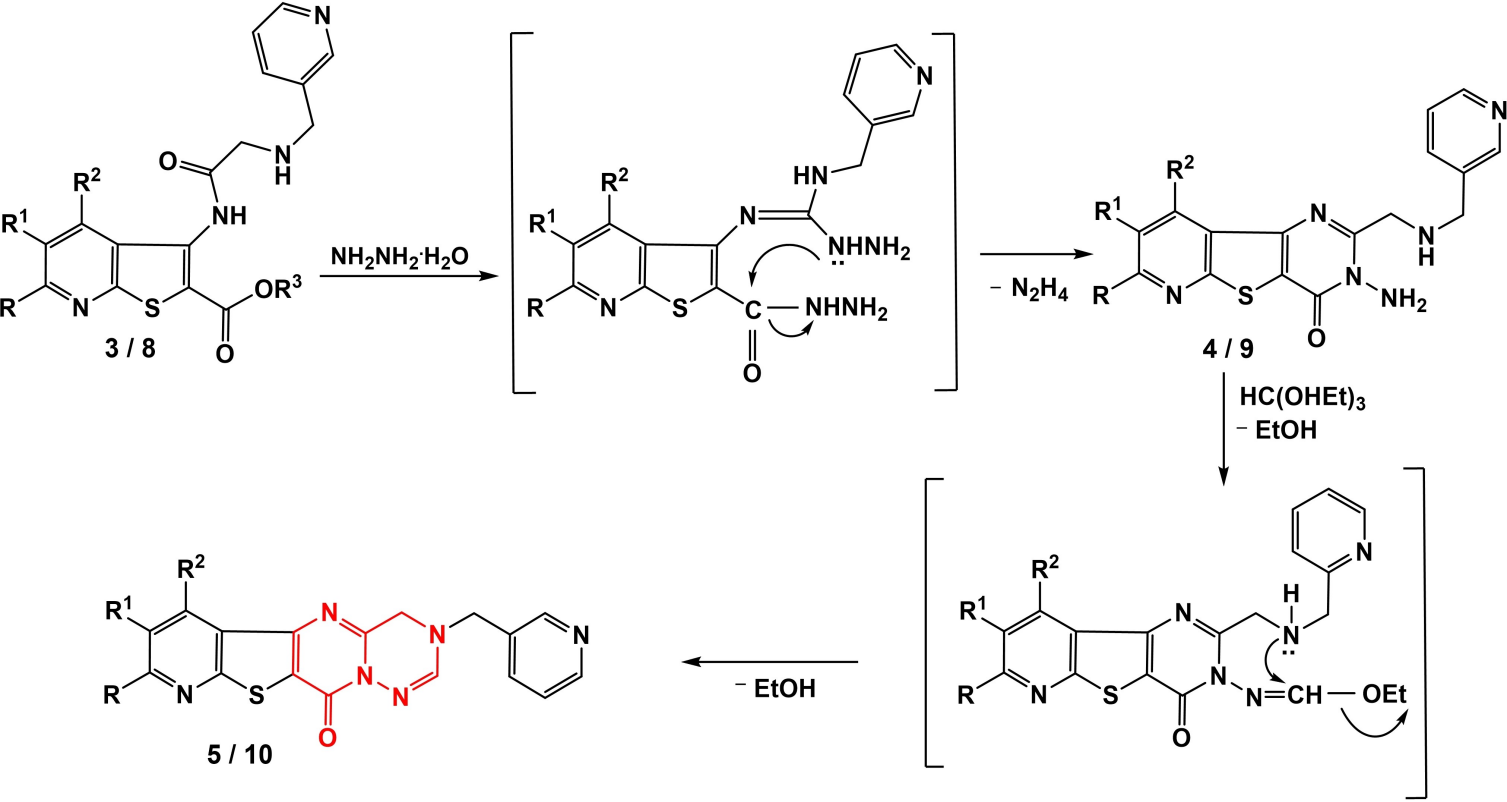
Mechanism proposed for the synthesis of **5/10** starting from compounds **3/8**.

The tentative mechanism concerning the reaction between aminoesters and hydrazine hydrate (leading to the synthesis of compounds **4/9**) is well‐documented in the existing literature^[32]^ and is illustrated below (Scheme 4). The suggested mechanism for the synthesis of compounds **5/10** can be delineated as follows.

The structure of the obtained compounds **4, 5, 9** and **10** was confirmed by the NMR, IR, MS spectra, and elemental analyses.

In the ^1^H NMR spectra of the compounds **4a–c** and **9a–d** the signals of the ester group are absent and the signals of the NH_2_ group appear at 5.91–6.16 ppm. In the ^1^H NMR spectra of the newly synthesized systems **5** and **10a–d**, the resonances corresponding to the NH_2_ and NH functional groups are notably absent; conversely, the resonance associated with the CH group of the triazine ring is observed at a chemical shift of 7.68–7.71 ppm, whereas the signals attributed to the NCH_2_ and CH_2_N groups are shifted to a weaker magnetic field, appearing at 4.30–4.38 ppm and 4.60–4.64 ppm, respectively.

In the IR spectrum, absorption of the NH_2_ and NH groups is absent, whereas absorption of the CO group is observed at 1664–1681 cm^−1^.

Thus, as a result of the study, five new heterocyclic systems were obtained.

In the future, it is planned to continue studies on fused furans and other heterocycles.

## Experimental Section

^1^H and ^13^C NMR spectra were recorded in DMSO/CCl_4_ (1/3) and CDCl_3_ solutions (300 MHz for ^1^H and 75 MHz for ^13^C, respectively) on a Mercury 300VX spectrometer (Varian Inc., Palo Alto, CA, USA). Chemical shifts were reported as *δ* (parts per million) relative to TMS as internal standard. IR spectra were recorded on Nicolet Avatar 330‐FT‐IR spectrophotometer (Thermo Nicolet, CA, USA) and the reported wave numbers were given in cm^‐1^. MS spectra were recorded on Waters Q‐Tof. All melting points were determined in an open capillary and were uncorrected. Elemental analyses were performed on an Elemental Analyzer Euro EA 3000. Compounds **1 a**,**b** ^[30]^ and **6 a–d**[^31^, ^33^] were already described. Physicochemical data for compound **8d** are not given, it did not crystallize and was isolated as an oil (yield 71 %).

**General Procedure for the Synthesis of Compounds 2 a,b** and **7a–d**. To a mixture of ester **1a,b** (**6a–d** for compounds **7a–d**) (50 mmol) and triethylamine (60 mmol) in anhydrous benzene (75 mL) chloroacetyl chloride (4.8 mL, 60 mmol), was added dropwise with stirring, and the mixture was stirred for 6 h at 35 °C. After cooling to room temperature and evaporation to dryness, the residue was treated with water (50 mL), and the precipitate was filtered off, washed with water, dried, and recrystallized from ethanol.

**General Procedure for the Synthesis of Compounds 3a–c and 8a–d**. A mixture of compound **2a,b** (**7a–d** for compounds **8a–d**) (5 mmol), the corresponding amine (5.5 mmol) and triethylamine (5.5 mmol) in absolute ethanol (50 mL) was refluxed for 3 h. The mixture was cooled, the solvent was distilled off to dryness, the residue was treated with water (50 mL), and the precipitate was filtered off, washed with water, dried, and recrystallized from ethanol.

**General Procedure for the Synthesis of Compounds 4a–c and 9a–d**. A mixture of compound **3a–c** (**8a–d** for compounds **9a–d**) (5 mmol) and hydrazine monohydrate (50 mmol) in ab‐solute ethanol (50 mL) was refluxed for 5 h. The mixture was cooled, and the precipitate was filtered off, washed with water, dried, and recrystallized from ethanol.

**General Procedure for the Synthesis of Compounds 5 and 10a–d**. A mixture of compound **4c** (**9a–d** for compounds **10a–d**) (1 mmol) and triethyl orthoformate (15 mL) was refluxed for 5 hours. The excess of triethyl orthoformate was distilled off, ethanol (15 mL) was added to the residue, the precipitated crystals filtered off, washed with water, dried and crystallized from ethanol.

## Conclusions

As a result of the research, a method was proposed that allows the synthesis of many new heterocyclic compounds. The synthesis of pyrimido[2,1‐*f*][1,2,4]triazines opens up new prospects for the synthesis of many new unique heterocyclic systems. The construction of these two cycles on any other cycles and heterocycles leads to completely new heterocyclic systems. An important condition for implementation is the presence of vicinal groups of amino esters and primary amines substituted in the 3^rd^ position of aminomethyl derivatives of thieno[3,2‐*d*]pyrimidines.


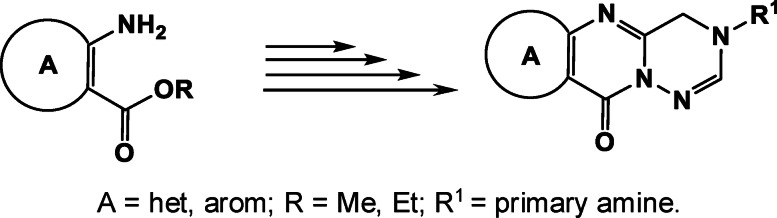


## Conflict of Interests

The authors declare no conflict of interest.

## Supporting information

As a service to our authors and readers, this journal provides supporting information supplied by the authors. Such materials are peer reviewed and may be re‐organized for online delivery, but are not copy‐edited or typeset. Technical support issues arising from supporting information (other than missing files) should be addressed to the authors.

Supporting Information

## Data Availability

The data that support the findings of this study are available from the corresponding author upon reasonable request.
