# Supplementary material for: A Universal Method for the Synthesis of new Heterocyclic Systems: Pyrimido[2,1‐f][1,2,4]triazines
Source: ChemistryOpen. 2025 Jan 31;14(6):e202400379. doi: 10.1002/open.202400379 (PMC12138049; doi:10.1002/open.202400379)
Supplement: Supplementary file 1 — Supporting Information [file OPEN-14-e202400379-s001.pdf]

ChemistryOpen

Supporting Information

A Universal Method for the Synthesis of new Heterocyclic Systems: Pyrimido[2,1-*f*][1,2,4]triazines

Samvel N. Sirakanyan,* Domenico Spinelli, Athina Geronikaki, Victor G. Kartsev,
Elmira K. Hakobyan,* Hasmik V. Jughetsyan, Hasmik A. Yegoryan, and Anush A. Hovakimyan

Supporting Information

A universal method for the synthesis of new heterocyclic systems: pyrimido[2,1-*f*][1,2,4]triazines

Samvel N. Sirakanyan,*[a] Domenico Spinelli,[b] Athina Geronikaki,[c] Victor G. Kartsev,[d] Elmira K. Hakobyan,*[a] Hasmik V. Jughetsyan,[a] Hasmik A. Yegoryan,[a] Anush A. Hovakimyan[a]

1 Scientific Technological Center of Organic and Pharmaceutical Chemistry of National Academy of Science of Republic of Armenia, Institute of Fine Organic Chemistry of A. L. Mnjoyan, Armenia 0014, Yerevan, Ave. Azatutyan 26; shnnr@mail.ru (S.N.S.); hakobyan.elmira@mail.ru (E.K.H.); jughetsyan2002@mail.ru (H.V.J.); hasmik.yegoryan@mail.ru (H.A.Y.); aaa.h.87@mail.ru (A.A.H.)

2 Dipartimento di Chimica G. Ciamician, Alma Mater Studiorum-Università di Bologna, Via F. Selmi 2, Bologna 40126, Italy; domenico.spinelli@unibo.it

3 Aristotle University of Thessaloniki, School of Pharmacy, Thessaloniki 54124, Greece; geronik@pharm.auth.gr

4 InterBioScreen, Moscow 119019, Russia; vkartsev@ibscreen.chg.ru

***Corresponding authors Emails:**

Samvel Sirakanyan – shnnr@mail.ru

Elmira Hakobyan – hakobyan.elmira@mail.ru

Table of Contents

1. General information......S2

2. General Procedure for the Synthesis of Compounds **2a, b** and **7a-d**......S2

3. General Procedure for the Synthesis of Compounds **3a–c** and **8a–d**......S3

4. General Procedure for the Synthesis of Compounds **4a–c** and **9a–d**......S5

5. General Procedure for the Synthesis of Compounds **5** and **10a–d**......S7

6. The copies of ^{1}H, ^{13}C NMR and MS spectra for all new synthesized compounds......S8

General Information

^{1}H and ^{13}C NMR spectra were recorded in DMSO/CCl4 (1/3) and CDCl3 solutions (300 MHz for ^{1}H and 75 MHz for ^{13}C, respectively) on a Mercury 300VX spectrometer (Varian Inc., Palo Alto, CA, USA). Chemical shifts were reported as *δ* (parts per million) relative to TMS as internal standard. IR spectra were recorded on Nicolet Avatar 330-FT-IR spectrophotometer (Thermo Nicolet, CA, USA) and the reported wave numbers were given in cm$^{\square 1}$. MS spectra were recorded on Waters Q-Tof. All melting points were determined in an open capillary and were uncorrected. Elemental analyses were performed on an Elemental Analyzer Euro EA 3000. Compounds **1a,b** and **6a–d**[30,31,33] were already described. Physicochemical data for compound **8d** are not given, it did not crystallize and was isolated as an oil (yield 71%).

General Procedure for the Synthesis of Compounds 2a,b and 7a–d

Chloroacetyl chloride (4.8 mL, 60mmol) was added dropwise with stirring to a mixture of ester **1a,b** (**6a–d** for compounds **7a-d**) (50 mmol) and triethylamine (60 mmol) in anhydrous benzene (75 mL), and the mixture was stirred for 6 h at 35°C. After cooling to room temperature and evaporation to dryness, the residue was treated with water (50 mL), and the precipitate was filtered off, washed withwater, dried, and recrystallized from ethanol.

Methyl 5,8,8-trimethyl-1-[(chloroacetyl)amino]-8,9-dihydro-6*H*-pyrano[4,3-*d*]thieno[2,3-*b*]pyridine-2-carboxylate (2a). Colorless solid, yield 79 %, m.p. 202–204 °C. IR *v*/cm^{-1}: 1688, 1709 (C=O), 3275 (NH). ^{1}H NMR (300 MHz, DMSO/CCl4, 1/3) δ 1.27 (s, 6H, C(CH3)2), 2.46 (s, 3H, CH3), 3.06 (s, 2H, CH2), 3.90 (s, 3H, OCH_3), 4.19 (s, 2H, CH_2Cl), 4.72 (s, 2H, OCH_2), 9.99 (br s, 1H, NH). Anal. calcd. for $C_{17}H_{19}ClN_2O_4S$: C 53.33; H 5.00; N 7.32%. Found: C 53.66; H 5.19; N 7.57%.

Ethyl 1-[(chloroacetyl)amino]-5-(2-furyl)-8,8-dimethyl-8,9-dihydro-6*H*-pyrano[4,3-*d*]thieno[2,3-*b*]pyridin-2-carboxylate (2b). Yellow solid, yield 81 %, m.p. 156–158 °C. IR *v*/cm^{-1}: 1695, 1701 (C=O), 3118, 3140 (NH). ^{1}H NMR (300 MHz, DMSO/CCl_4, 1/3) δ 1.30 (s, 6H, $C(CH_3)_2$), 1.41 (t, *J* = 7.1 Hz, 3H, $CH_2C\underline{H}_3$), 3.51 (s, 2H, CH_2), 4.20 (s, 2H, CH_2Cl), 4.35 (q, *J* = 7.1 Hz, 2H, $C\underline{H}_2CH_3$), 5.10 (s, 2H, OCH_2), 6.61 (dd, *J* = 3.5, 1.8 Hz, 1H, 4-CH_{furyl}), 7.19 (dd, *J* = 3.5, 0.8 Hz, 1H, 3-CH_{furyl}), 7.73 (dd, *J* = 1.8, 0.8 Hz, 1H, 5-CH_{furyl}), 10.03 (br s, 1H, NH). ^{13}C NMR (75 MHz, DMSO/CCl_4, 1/3) δ 13.7, 26.0, 35.3, 42.0, 60.6, 60.7, 68.1, 111.5, 112.5, 123.2, 124.7, 126.4, 132.9, 141.1, 143.8, 144.2, 152.9, 156.5, 160.4, 165.4. Anal. calcd. for $C_{21}H_{21}ClN_2O_5S$: C 56.18; H 4.72; N 6.24%. Found: C 56.54; H 4.93; N 6.51%.

Methyl 1-[(chloroacetyl)amino]-5-methyl-6,7,8,9-tetrahydrothieno[2,3-*c*]isoquinoline-2-carboxylate (7a). Colorless solid, yield 83 %, m.p. 161–163 °C. ^{1}H NMR (300 MHz, DMSO/CCl4, 1/3) δ 1.75–1.94 (m, 4H, 7, 8-CH_2), 2.51 (s, 3H, CH_3), 2.67–2.75 (m, 2H, 6-CH_2), 3.11–3.19 (m, 2H, 9-CH_2), 3.89 (s, 3H, OCH_3), 4.19 (s, 2H, CH_2Cl), 9.91 (br s, 1H, NH). Anal. calcd. for $C_{16}H_{17}ClN_2O_3S$: C 54.46; H 4.86; N 7.94%. Found: C 54.77; H 5.03; N 8.17%.

Ethyl 1-[(chloroacetyl)amino]-5-propyl-7,8-dihydro-6*H*-cyclopenta[*d*]thieno[2,3-*b*]pyridine-2-

carboxylate (7b). Colorless solid, yield 77 %, m.p. 168–170 °C. IR *v*/cm^{-1}: 1686, 1696 (C=O), 3252 (NH). ^{1}H NMR (300 MHz, DMSO/CCl_4, 1/3) δ 1.03 (t, 3H, *J* = 6.7 Hz, $CH_2CH_2C\underline{H}_3$), 1.41 (t, *J* = 7.1 Hz, 3H, $CH_2C\underline{H}_3$), 1.72–1.88 (m, 2H, $CH_2C\underline{H}_2CH_3$), 2.10–2.24 (m, 2H, 7-CH_2), 2.79 (t, *J* = 7.6 Hz, 2H, $C\underline{H}_2CH_2CH_3$), 2.98 (t, *J* = 7.4 Hz, 2H, 6-CH_2), 3.24 (t, *J* = 7.5 Hz, 2H, 8-CH_2), 4.22 (s, 2H, CH_2Cl), 4.35 (q, *J* = 7.1 Hz, 2H, $C\underline{H}_2CH_3$), 9.99 (br s, 1H, NH). ^{13}C NMR (75 MHz, DMSO/CCl_4, 1/3) δ 13.7, 13.8, 20.8, 23.7, 29.5, 30.9, 37.3, 42.0, 60.5, 121.9, 125.5, 132.6, 134.9, 149.0, 156.6, 158.3, 160.9, 165.1. Anal. calcd. for $C_{18}H_{21}ClN_2O_3S$: C 56.76; H 5.56; N 7.35%. Found: C 57.15; H 5.78; N 7.63%.

Ethyl 3-[(chloroacetyl)amino]-4,6-dimethylthieno[2,3-*b*]pyridine-2-carboxylate (7c). Colorless solid, yield 82 %, m.p. 194–196 °C. IR *v*/cm^{-1}: 1671, 1718 (C=O), 3238, 3434 (NH). ^{1}H NMR (300 MHz, DMSO/CCl_4, 1/3) δ 1.41 (t, *J* = 7.1 Hz, 3H, $CH_2C\underline{H}_3$), 2.59 (s, 3H, CH_3), 2.61 (s, 3H, CH_3), 4.22 (s, 2H, CH_2Cl), 4.35 (q, *J* = 7.1 Hz, 2H, $C\underline{H}_2CH_3$), 7.01 (s, 1H, 5-CH), 9.99 (br s, 1H, NH). ^{13}C NMR (75 MHz, DMSO/CCl4, 1/3) δ 13.8, 18.3, 23.6, 42.1, 60.6, 122.4, 122.9, 126.5, 133.3, 144.4, 158.2, 158.7, 160.9, 165.4. Anal. calcd. for $C_{14}H_{15}ClN_2O_3S$: C 51.45; H 4.63; N 8.57%. Found: C 51.77; H 4.81; N 8.81%.

Ethyl 1-[(chloroacetyl)amino]-7-isopropyl-5-piperidin-1-yl-6,7,8,9-tetrahydrothieno[2,3-c]-2,7-naphthyridine-2-carboxylate (7d). Yellow solid, yield 76 %, m.p. 200–202 °C. IR *v*/cm^{-1}: 1689, 1724 (C=O), 3243 (NH). ^{1}H NMR (300 MHz, DMSO/CCl_4, 1/3) δ 1.12 (d, *J* = 6.5 Hz, 6H, $CH(C\underline{H}_3)_2$), 1.39 (t, *J* = 7.1 Hz, 3H, $CH_2C\underline{H}_3$), 1.61–1.80 (m, 6H, $C_5H_{10}N$), 2.70–2.95 (m, 3H, $NC\underline{H}_2CH_2$, $C\underline{H}(CH_3)_2$), 3.10–3.25 (m, 6H, $NCH_2C\underline{H}_2$, $N(CH_2)_2$), 3.54 (s, 2H, NCH_2), 4.19 (s, 2H, CH_2Cl), 4.31 (q, *J* = 7.1 Hz, 2H, $C\underline{H}_2CH_3$), 9.84 (br s, 1H, NH). Anal. calcd. for $C_{23}H_{31}ClN_4O_3S$: C 57.67; H 6.52; N 11.70%. Found: C 58.02; H 6.72; N 11.96%.

General Procedure for the Synthesis of Compounds 3a–c and 8a–d

A mixture of compound **2a,b** (**7a–d** for compounds **8a-d**) (5 mmol), the corresponding amine (5.5 mmol) and triethylamine (5.5 mmol) in absolute ethanol (50 mL) was refluxed for 3 h. The mixture was cooled, the solvent was distilled off to dryness, the residue was treated with water (50 mL), and the precipitate was filtered off, washed with water, dried, and recrystallized from ethanol.

Methyl 1-[(*N*,*N*-diethylglycyl)amino]-5,8,8-trimethyl-8,9-dihydro-6*H*-pyrano[4,3-d]thieno[2,3-*b*]pyridine-2-carboxylate (3a). Colorless solid, yield 78 %, m.p. 167–169 °C. IR *v*/cm^{-1}: 1673, 1726 (C=O), 3199 (NH). ^{1}H NMR (300 MHz, DMSO/CCl_4, 1/3) δ 1.17 (t, *J* = 7.1 Hz, 6H, $2CH_2C\underline{H}_3$), 1.23 (s, 6H, $C(CH_3)_2$), 2.47 (s, 3H, CH_3), 2.72 (q, *J* = 7.1 Hz, 4H, $2C\underline{H}_2CH_3$), 3.02 (s, 2H, CH_2), 3.15 (s, 2H, $COCH_2$), 3.89 (s, 3H, OCH_3), 4.72 (s, 2H, OCH_2), 9.70 (br s, 1H, NH). ^{13}C NMR (75 MHz, DMSO/CCl_4, 1/3) δ 11.7, 20.8, 25.9, 34.8, 47.6, 51.5, 57.0, 60.1, 68.5, 119.8, 124.5, 126.0, 134.8, 139.2, 154.4, 156.3, 161.4, 171.1. Anal. calcd. for $C_{21}H_{29}N_3O_4S$: C 60.12; H 6.97; N 10.02%. Found: C 60.49; H 7.18; N 10.29%.

Methyl 5,8,8-trimethyl-1-[(morpholin-4-ylacetyl)amino]-8,9-dihydro-6*H*-pyrano[4,3-*d*]thieno[2,3-

***b*]pyridin-2-carboxylate (3b).** Colorless solid, yield 81 %, m.p. 220–222 °C. IR *ν*/cm^{-1}: 1675, 1720 (C=O), 3211 (NH). ^{1}H NMR (300 MHz, DMSO/CCl_4, 1/3) δ 1.23 (s, 6H, $C(CH_3)_2$), 2.47 (s, 3H, CH_3), 2.62–2.69 (m, 4H, $N(CH_2)_2$), 3.02 (s, 2H, CH_2), 3.12 (s, 2H, $COCH_2$), 3.72–3.77 (m, 4H, $O(CH_2)_2$), 3.89 (s, 3H, OCH_3), 4.72 (s, 2H, OCH_2), 9.78 (br s, 1H, NH). ^{13}C NMR (75 MHz, DMSO/CCl_4, 1/3) δ 20.8, 25.9, 34.8, 51.5, 53.4, 60.1, 61.8, 65.9, 68.5, 119.7, 124.3, 125.93, 134.7, 139.3, 154.4, 156.3, 161.5, 169.5. Anal. calcd. for $C_{21}H_{27}N_3O_5S$: C 58.18; H 6.28; N 9.69%. Found: C 58.51; H 6.47; N 9.93%.

Ethyl 5-(2-furyl)-8,8-dimethyl-1-{[*N*-(pyridin-3-ylmethyl)glycyl]amino}-8,9-dihydro-6*H*-pyrano[4,3-*d*]thieno[2,3-*b*]pyridine-2-carboxylate (3c). Colorless solid, yield 86 %, m.p. 199–201 °C. IR *ν*/cm^{-1}: 1692 (C=O), 3177 (NH). ^{1}H NMR (300 MHz, DMSO/CCl_4, 1/3) δ 1.28 (s, 6H, C(CH3)2), 1.42 (t, *J* =7.1 Hz, 3H, CH2C<u>H</u>3), 2.89 (br, 1H, NH), 3.24 (s, 2H, CH_2NH), 3.46 (s, 2H, CH_2), 3,93 (s, 2H, $NHCH_2$), 4.35 (q, 2H, *J* = 7.1, C<u>H</u>$_2$$CH_3$), 5.12 (s, 2H, OCH_2), 6.63 (dd, *J* = 3.5, 1.8 Hz, 1H, 4-CH_{furyl}), 7.22 (dd, *J* = 3.5, 0.8 Hz, 1H, 3-CH_{furyl}), 7.25 (dd, *J* = 7.8, 4.8 Hz, 1H, 5-H Py), 7.74 (dd, *J* = 1.8, 0.8 Hz, 1H, 5-CH_{furyl}), 7.77 (dt, *J* = 7.8, 1.8 Hz, 1H, 6-H Py), 8.49 (dd, *J* = 4.8, 1.8 Hz, 1H, 4-H Py), 8.58 (d, *J* = 1.8 Hz, 1H, 2-H Py), 9,91 (br s, 1H, NH). ^{13}C NMR (75 MHz, DMSO/CCl_4, 1/3) δ 13.8, 26.0, 35.2, 50.2, 51.6, 60.6, 68.1, 111.5, 112.5, 122.5, 122.6, 123.1, 126.5, 134.3, 134.8, 135.2, 141.2, 143.8, 144.2, 147.6, 149.3, 153.0, 156.5, 160.8, 171.3. Anal. calcd. for $C_{27}H_{28}N_4O_5S$: C 62.29; H 5.42; N 10.76%. Found: C 62.60; H 5.58; N 10.98%.

Methyl 5-methyl-1-{[*N*-(pyridin-3-ylmethyl)glycyl]amino}-6,7,8,9-tetrahydrothieno[2,3-*c*]isoquinoline-2-carboxylate (8a). Cream solid, yield 79 %, m.p. 184–186 °C. IR *ν*/cm^{-1}: 1683, 1724 (C=O), 3164, 3347 (NH). ^{1}H NMR (300 MHz, DMSO/CCl_4, 1/3) δ 1.69–1.95 (m, 4H, 7,8-CH_2), 2.51 (s, 3H, CH_3), 2.66–2.75 (m, 2H, 6-CH_2), 2.87 (br, 1H, NH), 3.10–3.18 (m, 2H, 9-CH_2), 3.36 (s, 2H, $COCH_2$), 3.88 (s, 2H, $NHCH_2$), 3.89 (s, 3H, OCH_3), 7.27 (dd, *J* = 7.8, 4.8 Hz, 1H, 5-H Py), 7.81 (dt, *J* = 7.8, 1.8 Hz, 1H, 6-H Py), 8.42 (dd, *J* = 4.8, 1.8 Hz, 1H, 4-H Py), 8.59 (d, *J* = 1.8 Hz, 1H, 2-H Py), 9.78 (br s, 1H, NHCO). ^{13}C NMR (75 MHz, DMSO/CCl_4, 1/3) δ 21.2, 21.3, 22.3, 24.5, 25.9, 38.7, 120.4, 122.6, 126.4, 127.3, 134.8, 135.0, 135.2, 143.1, 147.6, 149.3, 155.5, 157.9, 161.4, 171.2. Anal. calcd. for $C_{22}H_{24}N_4O_3S$: C 62.24; H 5.70; N 13.20%. Found: C 62.58; H 5.89; N 13.46%.

Ethyl 5-propyl-1-{[*N*-(pyridin-3-ylmethyl)glycyl]amino}-7,8-dihydro-6*H*-cyclopenta[*d*]thieno[2,3-*b*]pyridine-2-carboxylate (8b). Colorless solid, yield 82 %, m.p. 77–79 °C. IR *ν*/cm^{-1}: 1674, 1694 (C=O), 3267, 3342 (NH). ^{1}H NMR (300 MHz, DMSO/CCl4, 1/3) δ 1.03 (t, *J* = 6.7 Hz, 3H, CH2CH2C<u>H</u>3), 1.40 (t, *J* = 7.1 Hz, 3H, CH2C<u>H</u>3), 1.72–1.91 (m, 2H, CH2C<u>H</u>2CH3), 2.05–2.21 (m, 2H, 7-CH2), 2.78 (t, *J* = 7.6 Hz, 2H, C<u>H</u>$_2$$CH_2CH_3$), 2.83–2.96 (m, 3H, 6-$CH_2$, NH), 3.19 (t, *J* = 7.5 Hz, 2H, 8-CH_2), 3.37 (s, 2H, $COCH_2$), 3.89 (s, 2H, $NHCH_2$), 4.35 (q, *J* = 7.1 Hz, 2H, C<u>H</u>$_2$$CH_3$), 7.28 (dd, *J* = 7.8, 4.8 Hz, 1H, 5-H Py), 7.81 (dt, *J* = 7.8, 1.8 Hz, 1H, 6-H Py), 8.42 (dd, *J* = 4.8, 1.8 Hz, 1H, 4-H Py), 8.59 (d, *J* = 1.8 Hz, 1H, 2-H Py), 9.93 (br s, 1H, NH). ^{13}C NMR (75 MHz, DMSO/CCl_4, 1/3) δ 13.7, 13.9, 20.8, 23.8, 29.6, 31.2, 37.4, 50.3, 51.6, 60.5, 119.3, 122.6, 125.6,

134.2, 134.7, 134.8, 135.2, 147.6, 149.3, 149.4, 156.7, 158.2, 161.4, 170.9. Anal. calcd. for $C_{24}H_{28}N_4O_3S$: C 63.69; H 6.24; N 12.38%. Found: C 64.01; H 6.42; N 12.62%.

Ethyl 4,6-dimethyl-3-{[*N*-(pyridin-3-ylmethyl)glycyl]amino}thieno[2,3-*b*]pyridine-2-carboxylate (8c). Yellow solid, yield 84 %, m.p. 119–121 °C. IR *v*/cm^{-1}: 1670, 1708 (C=O), 3249, 3321 (NH). ^{1}H NMR (300 MHz, DMSO/CCl$_4$, 1/3) δ 1.40 (t, *J* = 7.1 Hz, 3H, CH$_2$C<u>H</u>$_3$), 2.59 (s, 3H, CH$_3$), 2.61 (s, 3H, CH$_3$), 2.92 (br s, 1H, NH), 3.36 (s, 2H, CH$_2$NH), 3.89 (s, 2H, NHCH$_2$), 4.35 (q, *J* = 7.1 Hz, 2H, C<u>H</u>$_2$CH$_3$), 6.99 (s, 1H, 5-CH), 7.27 (dd, *J* = 7.8, 4.8 Hz, 1H, 5-H Py), 7.81 (dt, *J* = 7.8, 1.8 Hz, 1H, 6-H Py), 8.43 (dd, *J* = 4.8, 1.8 Hz, 1H, 4-H Py), 8.58 (d, *J* = 1.8 Hz, 1H, 2-H Py), 9.89 (br s, 1H, NHCO). ^{13}C NMR (75 MHz, DMSO/CCl$_4$, 1/3) δ 13.8, 18.3, 23.6, 50.3, 51.6, 60.5, 120.4, 122.2, 122.6, 126.5, 134.8, 134.9, 135.2, 144.6, 147.6, 149.3, 158.1, 158.7, 161.1, 171.2. Anal. calcd. for $C_{20}H_{22}N_4O_3S$: C 60.28; H 5.56; N 14.06%. Found: C 60.68; H 5.79; N 14.35%.

General Procedure for the Synthesis of Compounds 4a–c and 9a–d

A mixture of compound **3a–c** (**8a–d** for compounds **9a-d**) (5 mmol) and hydrazine monohydrate (50 mmol) in absolute ethanol (50 mL) was refluxed for 5 h. The mixture was cooled, and the precipitate was filtered off, washed with water, dried, and recrystallized from ethanol.

9-Amino-10-[(diethylamino)methyl]-2,2,5-trimethyl-1,4-dihydro-2*H*-pyrano[4'',3'':4',5']pyrido[3',2':4,5]thieno[3,2-*d*]pyrimidin-8(9*H*)-one (4a). Colorless solid, yield 77 %, m.p. 236–238 °C IR *v*/cm^{-1}: 1668 (C=O), 3211, 3306 (NH2). ^{1}H NMR (300 MHz, DMSO/CCl$_4$, 1/3) δ 1.11 (t, *J* = 7.1 Hz, 6H, 2CH$_2$C<u>H</u>$_3$), 1.32 (s, 6H, C(CH$_3$)$_2$), 2.50 (s, 3H, CH$_3$), 2.73 (q, *J* = 7.1 Hz, 4H, 2C<u>H</u>$_2$CH$_3$), 3.44 (s, 2H, CH$_2$), 3.97 (s, 2H, NCH$_2$), 4.76 (s, 2H, OCH$_2$), 6.41 (br s, 2H, NH$_2$). ^{13}C NMR (75 MHz, DMSO/CCl$_4$, 1/3) δ 11.2, 20.9, 26.0, 46.3, 55.4, 60.0, 68.6, 118.1, 123.1, 124.2, 140.2, 148.9, 152.5, 154.5, 154.7, 159.3. Anal. calcd. for $C_{20}H_{27}N_5O_2S$: C 59.83; H 6.78; N 17.44%. Found: C 60.21; H 7.00; N 17.71%.

9-Amino-2,2,5-trimethyl-10-(morpholin-4-ylmethyl)-1,4-dihydro-2*H*-pyrano[4'',3'':4',5']pyrido[3',2':4,5]thieno[3,2-*d*]pyrimidin-8(9*H*)-one (4b). Colorless solid, yield 75 %, m.p. 246–248 °C. IR *v*/cm^{-1}: 1672 (C=O), 3300, 3542 (NH2). ^{1}H NMR (300 MHz, DMSO/CCl$_4$, 1/3) δ 1.33 (s, 6H, C(CH$_3$)$_2$), 2.50 (s, 3H, CH$_3$), 2.67–2.73 (m, 4H, N(CH$_2$)$_2$), 3.43 (s, 2H, CH$_2$), 3.63–3.69 (m, 4H, O(CH$_2$)$_2$), 3.89 (s, 2H, NCH$_2$), 4.75 (s, 2H, CH$_2$), 6.16 (br s, 2H, NH$_2$). ^{13}C NMR (75 MHz, DMSO/CCl$_4$, 1/3) δ 21.0, 26.1, 36.4, 52.6, 59.4, 59.9, 66.1, 68.7, 117.9, 123.1, 124.6, 140.4, 149.1, 153.1, 155.0, 155.6, 159.1. Anal. calcd. for $C_{20}H_{25}N_5O_3S$: C 57.81; H 6.06; N 16.85%. Found: C 58.13; H 6.25; N 17.08%.

9-Amino-5-(2-furyl)-2,2-dimethyl-10-{[(pyridin-3-ylmethyl)amino]methyl}-1,4-dihydro-2*H*-pyrano[4'',3'':4',5']pyrido[3',2':4,5]thieno[3,2-*d*]pyrimidin-8(9*H*)-one (4c). Colorless solid, yield 84 %, m.p. 257–259 °C. IR *v*/cm^{-1}: 1669 (C=O), 3190, 3302 (NH). ^{1}H NMR (300 MHz, DMSO/CCl$_4$, 1/3) δ 1.27 (s, 6H, C(CH$_3$)$_2$), 2.91 (br, 1H, NH), 3.48 (s, 2H, CH$_2$), 3.90 (s, 2H,

C$\underline{H}_2$NH), 4.09 (s, 2H, NHC$\underline{H}_2$), 5.11 (s, 2H, OCH_2), 5.96 (s, 2H, NH_2), 6.62 (dd, *J* = 3.5, 1.8 Hz, 1H, 4-CH_{furyl}), 7.22 (dd, *J* = 3.5, 0.8 Hz, 1H, 3-CH_{furyl}), 7.24 (dd, *J* = 7.8, 4.8 Hz, 1H, 5-H Py), 7.73 (dd, *J* = 1.8, 0.8 Hz, 1H, 5-CH_{furyl}), 7.75 (dt, *J* = 7.8, 1.8 Hz, 1H, 6-H Py), 8.41 (dd, *J* = 4.8, 1.8 Hz, 1H, 4-H Py), 8.54 (d, *J* = 1.8 Hz, 1H, 2-H Py). ^{13}C NMR (75 MHz, DMSO/CCl_4, 1/3) δ 25.9, 36.9, 38.7, 49.3, 49.6, 60.5, 68.1, 111.6, 112.6, 118.9, 122.2, 123.0, 123.5, 134.9, 135.1, 142.5, 143.8, 144.4, 147.5, 149.1, 149.2, 153.1, 156.2, 156.3, 159.6. Anal. calcd. for $C_{25}H_{24}N_6O_3S$: C 61.46; H 4.95; N 17.20%. Found: C 61.81; H 5.15; N 17.44%.

9-Amino-5-methyl-10-{[(pyridin-3-ylmethyl)amino]methyl}-1,2,3,4-tetrahydropyrimido[4',5':4,5]thieno[2,3-*c*]isoquinolin-8(9*H*)-one (9a). Yellow solid, yield 79 %, m.p. 254–256 °C. IR *v*/cm^{-1}: 1681 (C=O), 3190, 3270, 3300 (NH, NH_2). ^{1}H NMR (300 MHz, DMSO/CCl_4, 1/3) δ 1.77–1.97 (m, 4H, 7,8-CH_2), 2.53 (s, 3H, CH_3), 2.69–2.78 (m, 2H, 6-CH_2), 2.86 (br, 1H, NH), 3.49–3.56 (m, 2H, 9-CH_2), 3.88 (s, 2H, CH_2NH), 4.08 (s, 2H, NHCH_2), 5.91 (s, 2H, NH_2), 7.23 (dd, *J* = 7.8, 4.8 Hz, 1H, 5-H Py), 7.74 (dt, *J* = 7.8, 1.8 Hz, 1H, 6-H Py), 8.39 (dd, *J* = 4.8, 1.8 Hz, 1H, 4-H Py), 8.51 (d, *J* = 1.8 Hz, 1H, 2-H Py). ^{13}C NMR (75 MHz, DMSO/CCl_4, 1/3) δ 21.1, 21.9, 22.3, 25.7, 26.4, 26.4, 49.6, 49.9, 117.9, 122.4, 123.4, 127.2, 135.0, 135.1, 144.3, 147.5, 149.2, 149.7, 155.7, 156.3, 158.1, 158.5. Anal. calcd. for $C_{21}H_{22}N_6OS$: C 62.05; H 5.45; N 20.67%. Found: C 62.38; H 5.63; N 20.89%. ESI HRMS [$C_{21}H_{22}N_6OS+H^+$] Calculated: 408.1681. Found: 408.1693.

8-Amino-4-propyl-9-{[(pyridin-3-ylmethyl)amino]methyl}-2,3-dihydro-1*H*-cyclopenta[4',5']pyrido[3',2':4,5]thieno[3,2-*d*]pyrimidin-7(8*H*)-one (9b). Colorless solid, yield 83 %, m.p. 205–207 °C. IR *v*/cm^{-1}: 1662 (C=O), 3280, 3342 (NH, NH_2). ^{1}H NMR (300 MHz, DMSO/CCl_4, 1/3) δ 1.03 (t, *J* = 6.7, 3H, $CH_2CH_2C\underline{H}_3$), 1.75–1.89 (m, 2H, $CH_2C\underline{H}_2CH_3$), 2.18–2.32 (m, 2H, 7-CH_2), 2.82 (t, *J* = 7.6 Hz, 2H, $C\underline{H}_2CH_2CH_3$), 2.87 (br, 1H, NH), 3.01 (t, *J* = 7.4 Hz, 2H, 6-CH_2), 3.50 (t, *J* = 7.5 Hz, 2H, 8-CH_2), 3.87 (s, 2H, CH_2NH), 4.06 (s, 2H, NHCH_2), 5.92 (s, 2H, NH_2), 7.24 (dd, *J* = 7.8, 4.8 Hz, 1H, 5-H Py), 7.74 (dt, *J* = 7.8, 1.8 Hz, 1H, 6-H Py), 8.39 (dd, *J* = 4.8, 1.8 Hz, 1H, 4-H Py), 8.51 (d, *J* = 1.8 Hz, 1H, 2-H Py).^{13}C NMR (75 MHz, DMSO/CCl_4, 1/3) δ 13.6, 20.9, 24.0, 29.5, 31.5, 31.5, 37.4, 49.5, 49.8, 118.1, 122.5, 122.9, 134.9, 135.0, 147.5, 148.9, 149.2, 150.1, 156.3, 156.6, 158.6, 159.7. Anal. calcd. for $C_{22}H_{24}N_6OS$: C 62.83; H 5.75; N 19.98%. Found: C 63.19; H 5.96; N 20.23%. ESI HRMS [$C_{22}H_{24}N_6OS+H^+$] Calculated: 422.1838. Found: 422.1876.

3-Amino-7,9-dimethyl-2-{[(pyridin-3-ylmethyl)amino]methyl}pyrido[3',2':4,5]thieno[3,2-*d*]pyrimidin-4(3*H*)-one (9c). Yellow solid, yield 78 %, m.p. 189–191 °C. IR *v*/cm^{-1}: 1665 (C=O), 3186, 3310 (NH). ^{1}H NMR (300 MHz, DMSO/CCl_4, 1/3) δ 2.60 (s, 3H, CH_3), 2.89 (s, 3H, CH_3), 2.96 (br s, 1H, NH), 3.88 (s, 2H, C$\underline{H}_2$NH), 4.07 (s, 2H, NHC$\underline{H}_2$), 5.94 (s, 2H, NH_2), 7.06 (s, 1H, 5-CH), 7.23 (dd, *J* = 7.8, 4.8 Hz, 1H, 5-H Py), 7.74 (dt, *J* = 7.8, 1.8 Hz, 1H, 6-H Py), 8.38 (dd, *J* = 4.8, 1.8 Hz, 1H, 4-H Py), 8.51 (d, *J* = 1.8 Hz, 1H, 2-H Py). ^{13}C NMR (75 MHz, DMSO/CCl_4, 1/3) δ 18.7, 23.8, 49.5, 49.9, 117.9, 121.8, 122.5, 123.7, 135.1, 145.7, 147.5, 149.2, 149.5, 156.2,

156.3, 158.6, 161.9. Anal. calcd. for $C_{18}H_{18}N_6OS$: C 59.00; H 4.95; N 22.93%. Found: C 59.31; H 5.11; N 23.15%. ESI HRMS [$C_{18}H_{18}N_6OS+H^+$] Calculated: 367.1341. Found: 367.1361.

9-Amino-3-isopropyl-5-piperidin-1-yl-10-{[(pyridin-3-ylmethyl)amino]methyl}-1,2,3,4-tetrahydropyrimido[4',5':4,5]thieno[2,3-*c*]-2,7-naphthyridin-8(9*H*)-one (9d). Yellow solid, yield 80 %, m.p. 111–113 °C. IR *v*/cm^{-1}: 1659 (C=O), 3177, 3304 (NH). ^{1}H NMR (300 MHz, DMSO/CCl_4, 1/3 δ 1.14 (d, *J* = 6.5 Hz, 6H, CH(C<u>H</u>$_3$)$_2$), 1.60–1.84 (m, 6H, $C_5H_{10}N$), 2.64–2.98 (m, 4H, NC<u>H</u>$_2$CH$_2$, NH, C<u>H</u>(CH$_3$)$_2$), 3.13–3.25 (m, 4H, N(CH$_2$)$_2$), 3.44 (t, *J* = 5.9 Hz, 2H, NCH$_2$C<u>H</u>$_2$), 3.55 (s, 2H, NCH$_2$), 3.87 (s, 2H, C<u>H</u>$_2$NH), 4.01 (s, 2H, NHC<u>H</u>$_2$), 5.80 (s, 2H, NH$_2$), 7.23 (dd, *J* = 7.8, 4.8 Hz, 1H, 5-H Py), 7.74 (dt, *J* = 7.8, 1.8 Hz, 1H, 6-H Py), 8.40 (dd, *J* = 4.8, 1.8 Hz, 1H, 4-H Py), 8.53 (d, *J* = 1.8 Hz, 1H, 2-H Py). ^{13}C NMR (75 MHz, DMSO/CCl_4, 1/3) δ 18.2, 24.0, 25.6, 28.0, 45.0, 48.5, 49.6, 49.7, 50.6, 53.4, 115.9, 119.8, 120.6, 122.4, 135.0, 135.2, 143.6, 147.5, 149.2, 149.9, 155.9, 156.3, 158.9, 160.7. Anal. calcd. for $C_{27}H_{34}N_8OS$: C 62.52; H 6.61; N 21.60%. Found: C 62.85; H 6.79; N 21.84%. ESI HRMS [$C_{27}H_{34}N_8OS+H^+$] Calculated: 519.2654. Found: 519.2657.

General Procedure for the Synthesis of Compounds 5 and 10a–d

A mixture of compound **4c** (**9a–d** for compounds **10a-d**) (1 mmol) and triethyl orthoformate (15 mL) was refluxed for 5 hours. The excess of triethyl orthoformate was distilled off, ethanol (15 mL) was added to the residue, the precipitated crystals filtered off, washed with water, dried and crystallized from ethanol.

5-(2-Furyl)-2,2-dimethyl-12-(pyridin-3-ylmethyl)-1,4,12,13-tetrahydro-2*H*,8*H*-pyrano[4''',3''':4'',5'']pyrido[3'',2'':4',5']thieno[3',2':4,5]pyrimido[2,1-*f*][1,2,4]triazin-8-one (5). Colorless solid, yield 58 %, m.p. > 350 °C. IR *v*/cm^{-1}: 1679 (C=O). ^{1}H NMR (300 MHz, DMSO/CCl_4, 1/3) 1.33 (s, 6H, C(CH$_3$)$_2$), 3.39 s, (2H, CH$_2$), 4.38 (s, 2H, NCH$_2$), 4.64 (s, 2H, CH$_2$N), 5.09 (s, 2H, OCH$_2$), 6.63 (dd, *J* = 3.5, 1.8 Hz, 1H, 4-CH_{furyl}), 7.20 (dd, *J* = 3.5, 0.8 Hz, 1H, 3-CH_{furyl}), 7.41 (dd, *J* = 7.8, 4.8 Hz, 1H, 5-H Py), 7.70 (s, 1H, N=CH), 7.74 (dd, *J* = 1.8, 0.8 Hz, 1H, 5-CH_{furyl}), 7.90 (dt, *J* = 7.8, 1.8 Hz, 1H, 6-H Py), 8.54 (dd, *J* = 4.8, 1.8 Hz, 1H, 4-H Py), 8.67 (d, *J* = 1.8 Hz, 1H, 2-H Py). Anal. calcd. for $C_{26}H_{22}N_6O_3S$: C 62.64; H 4.45; N 16.86%. Found: C 63.01; H 4.68; N 17.12%. ESI HRMS [$C_{26}H_{22}N_6O_3S+H^+$] Calculated: 499.1552. Found: 499.1548.

5-Methyl-12-(pyridin-3-ylmethyl)-1,2,3,4,12,13-hexahydro-8*H*-[1,2,4]triazino[1'',6'':1',2']pyrimido[4',5':4,5]thieno[2,3-*c*]isoquinolin-8-one (10a). Colorless solid, yield 61 %, m.p. 312–314 °C. IR *v*/cm^{-1}: 1681 (C=O). ^{1}H NMR (300 MHz, DMSO/CCl_4, 1/3) δ 1.71–1.86 (m, 4H, 7,8-CH$_2$), 2.49 (s, 3H, CH$_3$), 2.69 (br t, 2H, *J* = 6.0, 6-CH$_2$), 3.34 (br t, *J* = 6.0 Hz, 2H, 9-CH$_2$), 4.34 (s, 2H, NCH$_2$), 4.60 (s, 2H, CH$_2$N), 7.45 (dd, *J* = 7.8, 4.8 Hz, 1H, 5-H Py), 7.71 (s, 1H, N=CH), 7.91 (dt, *J* = 7.8, 1.8 Hz, 1H, 6-H Py), 8.58 (dd, *J* = 4.8, 1.8 Hz, 1H, 4-H Py), 8.69 (d, *J* = 1.8 Hz, 1H, 2-H Py). Anal. calcd for $C_{22}H_{20}N_6OS$: C 63.44; H 4.84; N 20.19%. Found: C 63.76; H 5.04; N 20.42%. ESI HRMS [$C_{22}H_{20}N_6OS+H^+$]Calculated: 417.1497.

Found: 417.1499.

Propyl-11-(pyridin-3-ylmethyl)-2,3,11,12-tetrahydrocyclopenta[4'',5'']pyrido[3'',2'':4',5']thieno[3',2':4,5]pyrimido[2,1-*f*][1,2,4]triazin-7(1*H*)-one (10b). Colorless solid, yield 63 %, m.p. 268–270 °C. IR *ν*/cm^{-1}: 1678 (C=O). ^{1}H NMR (300 MHz, DMSO/CCl_4, 1/3) δ 0.99 (t, *J* = 6.7 Hz, 3H, $CH_2CH_2C\underline{H}_3$), 1.69–1.84 (m, 2H, $CH_2C\underline{H}_2CH_3$), 2.09–2.24 (m, 2H, 7-CH_2), 2.76 (t, *J* = 7.6 Hz, 2H, $C\underline{H}_2CH_2CH_3$), 2.93 (t, *J* = 7.4 Hz, 2H, 6-CH_2), 3.33 (t, *J* = 7.5 Hz, 2H, 8- CH_2), 4.30 (s, 2H, NCH_2), 4.61 (s, 2H, CH_2N), 7.40 (dd, *J* = 7.8, 4.8 Hz, 1H, 5-H Py), 7.68 (s, 1H, N=CH), 7.88 (dt, *J* = 7.8, 1.8 Hz, 1H, 6-H Py), 8.54 (dd, *J* = 4.8, 1.8 Hz, 1H, 4-H Py), 8.65 (d, *J* = 1.8 Hz, 1H, 2-H Py). ^{13}C NMR (75 MHz, DMSO/CCl_4, 1/3) δ 13.66, 20.9, 23.8, 29.4, 31.5, 37.3, 44.6, 52.2, 120.5, 122.5, 123.3, 130.0, 135.0, 135.7, 144.1, 144.6, 148.0, 149.0, 149.4, 149.9, 152.5, 158.5, 159.3. Anal. calcd. for $C_{23}H_{22}N_6OS$: C 64.16; H 57.15; N 19.52%. Found: C 64.55; H 5.37; N 19.80%. ESI HRMS [$C_{23}H_{22}N_6OS+H^+$] Calculated: 431.1654. Found: 431.1647.

6,8-Dimethyl-3-(pyridin-3-ylmethyl)-3,4-dihydro-11*H*-pyrido[3'',2'':4',5']thieno[3',2':4,5]pyrimido[2,1-*f*][1,2,4]triazin-11-one (10c). Cream solid, yield 59 %, m.p. 310–312 °C. IR *ν*/cm^{-1}: 1664 (C=O). ^{1}H NMR (300 MHz, DMSO/CCl_4, 1/3) δ 2.54 (s, 3H, CH_3), 2.71 (s, 3H, CH_3), 4.33 (s, 2H, NCH_2), 4.60 (s, 2H, CH_2N), 7.16 (s, 1H, 5-CH), 7.45 (dd, *J* = 7.8, 4.8 Hz, 1H, 5-H Py), 7.71 (s, 1H, N=CH), 7.91 (dt, *J* = 7.8, 1.8 Hz, 1H, 6-H Py), 8.58 (dd, *J* = 4.8, 1.8 Hz, 1H, 4-H Py), 8.69 (d, *J* = 1.8 Hz, 1H, 2-H Py). Anal. calcd. for $C_{19}H_{16}N_6OS$: C 60.62; H 4.28; N 22.33%. Found: C 60.94; H 4.46; N 22.57%. ESI HRMS [$C_{19}H_{16}N_6OS+H^+$] Calculated: 377.1185. Found: 377.1184.

3-Isopropyl-5-piperidin-1-yl-12-(pyridin-3-ylmethyl)-1,2,3,4,12,13-hexahydro-8*H*-[1,2,4]triazino[1'',6'':1',2']pyrimido[4',5':4,5]thieno[2,3-*c*]-2,7-naphthyridin-8-one (10d). Yellow solid, yield 57 %, m.p. > 350 °C. IR *ν*/cm^{-1}: 1671 (C=O). ^{1}H NMR (300 MHz, $CDCl_3$) δ 1.09 (d, *J* = 6.5 Hz, 6H, $CH(C\underline{H}_3)_2$), 1.49–1.83 (m, 6H, $C_5H_{10}N$), 2.68–2.99 (m, 3H, $NC\underline{H}_2CH_2$, $C\underline{H}(CH_3)_2$), 3.11–3.27 (m, 4H, $N(CH_2)_2$), 3.41 (t, *J* = 5.9, 2H, $NCH_2C\underline{H}_2$), 3.62 (s, 2H, NCH_2), 4.26 (s, 2H, NCH_2), 4.59 (s, 2H, CH_2N), 7.45 (dd, *J* = 7.8, 4.8 Hz, 1H, 5-H Py), 7.67 (s, 1H, N=CH), 7.86 (dt, *J* = 7.8, 1.8 Hz, 1H, 6-H Py), 8.67–8.84 (m, 2H, 4-H Py, 2-H Py). ^{13}C NMR (75 MHz, DMSO/CCl_4, 1/3) δ 18.8, 18.9, 24.7, 26.3, 28.3, 45.6, 45.7, 49.4, 51.2, 54.1, 54.3, 119.7, 120.1, 120.9, 124.3, 129.3, 135.8, 142.0, 143.9, 144.0, 149.7, 150.0, 150.6, 154.2, 160.6, 161.9. Anal. calcd. for $C_{28}H_{32}N_8OS$: C 63.61; H 6.10; N 21.20%. Found: C 63.96; H 6.31; N 21.46%. ESI HRMS [$C_{28}H_{32}N_8OS+H^+$] Calculated: 529.2498. Found: 529.2504.

The copies of ^{1}H, ^{13}C NMR and MS spectra for all new synthesized compounds

Molecular Structure Research Centre, Yerevan, Armenia, Varian Mercury-300

H1 300.077 MHz, nt = 16, np = 16000, temp = 30.0 C, lb = -0.3, solvent = DMSO

Nov 10 2005

SAMV_05 s05-054

- S05-054

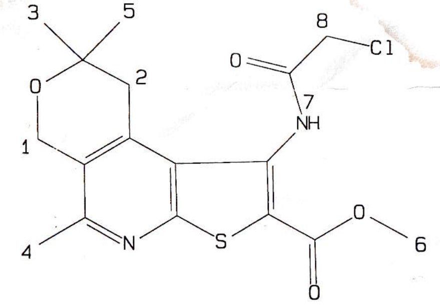

$C_{17}H_{19}ClN_2O_4S$

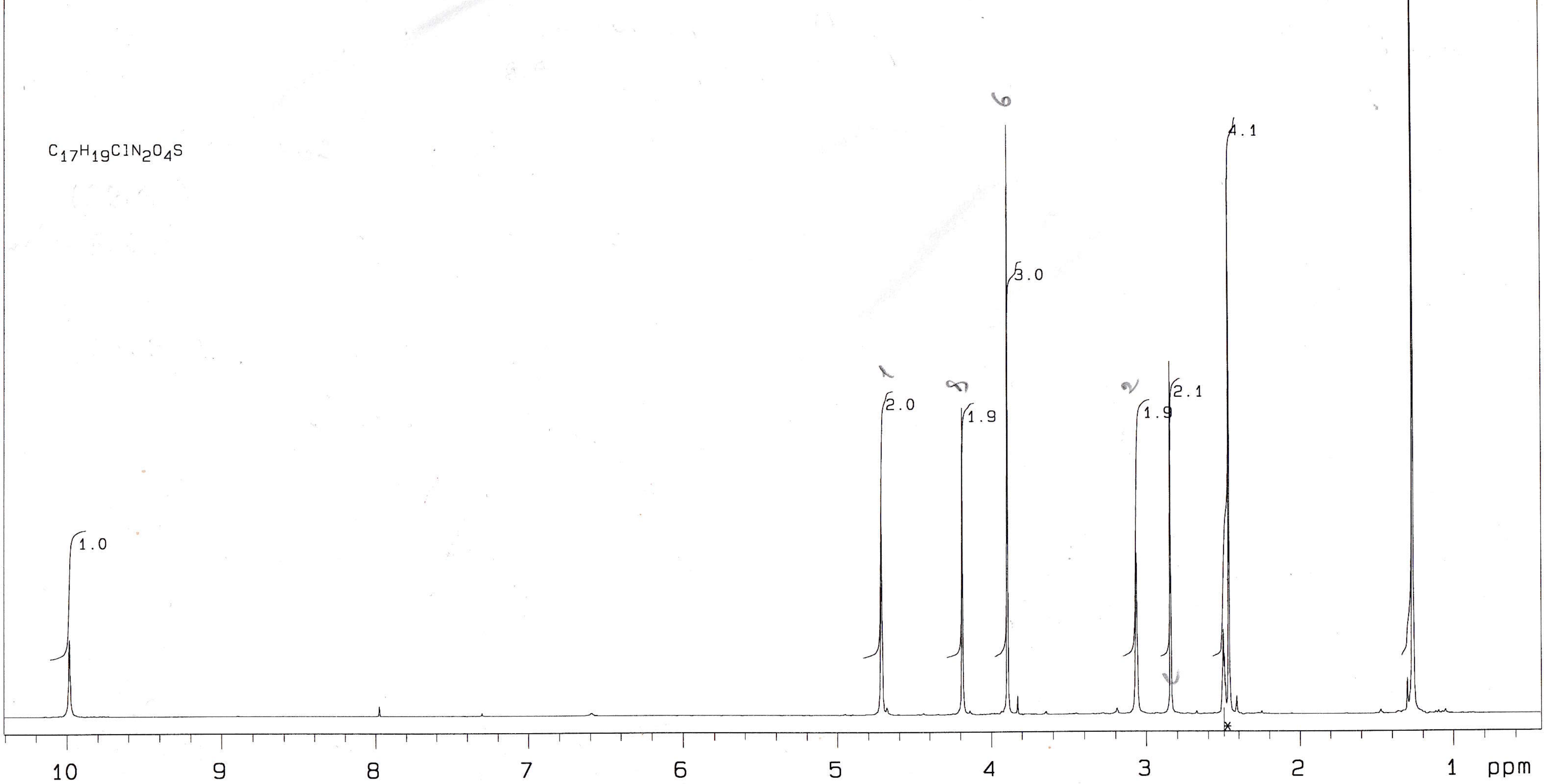

2b

Molecular Structure Research Centre, Yerevan, Armenia, Varian Mercury-300VX

H1 300.088 MHz, nt=16, np=32000, temp=30.0 C, lb=-0.2, solvent=DMSO/CCl4 1/3

Jun 4 2019

NOCI_19 tem-059-1

TEM-059-1

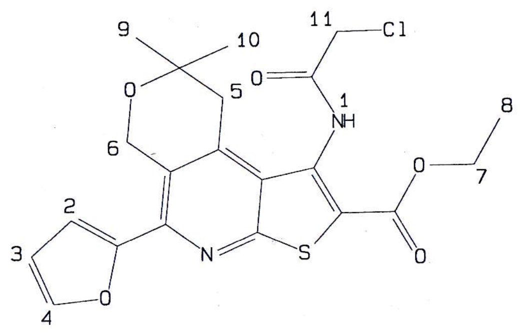

$C_{21}H_{21}ClN_2O_5S$

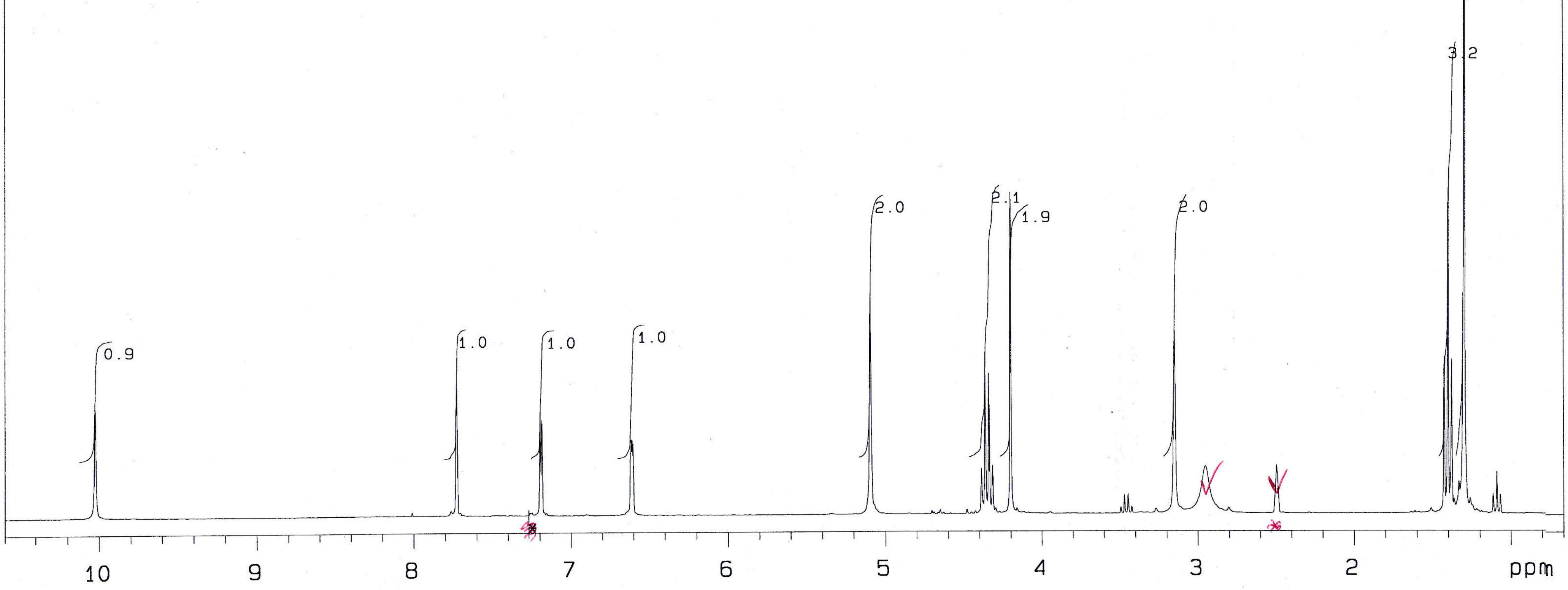

Molecular Structure Research Centre, Yerevan, Armenia, Varian Mercury-300VX

C13 75.465 MHz, nt=848, np=19998, temp=30.0 C, lb=1.0, solvent=DMSO/CCl4 1/3

Jun 4 2019

NOCI_19 tem-059-1

TEM-059-1

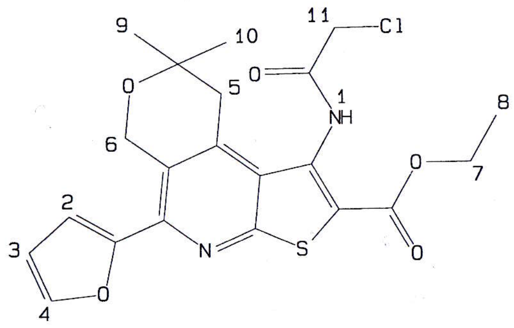

$C_{21}H_{21}ClN_2O_5S$

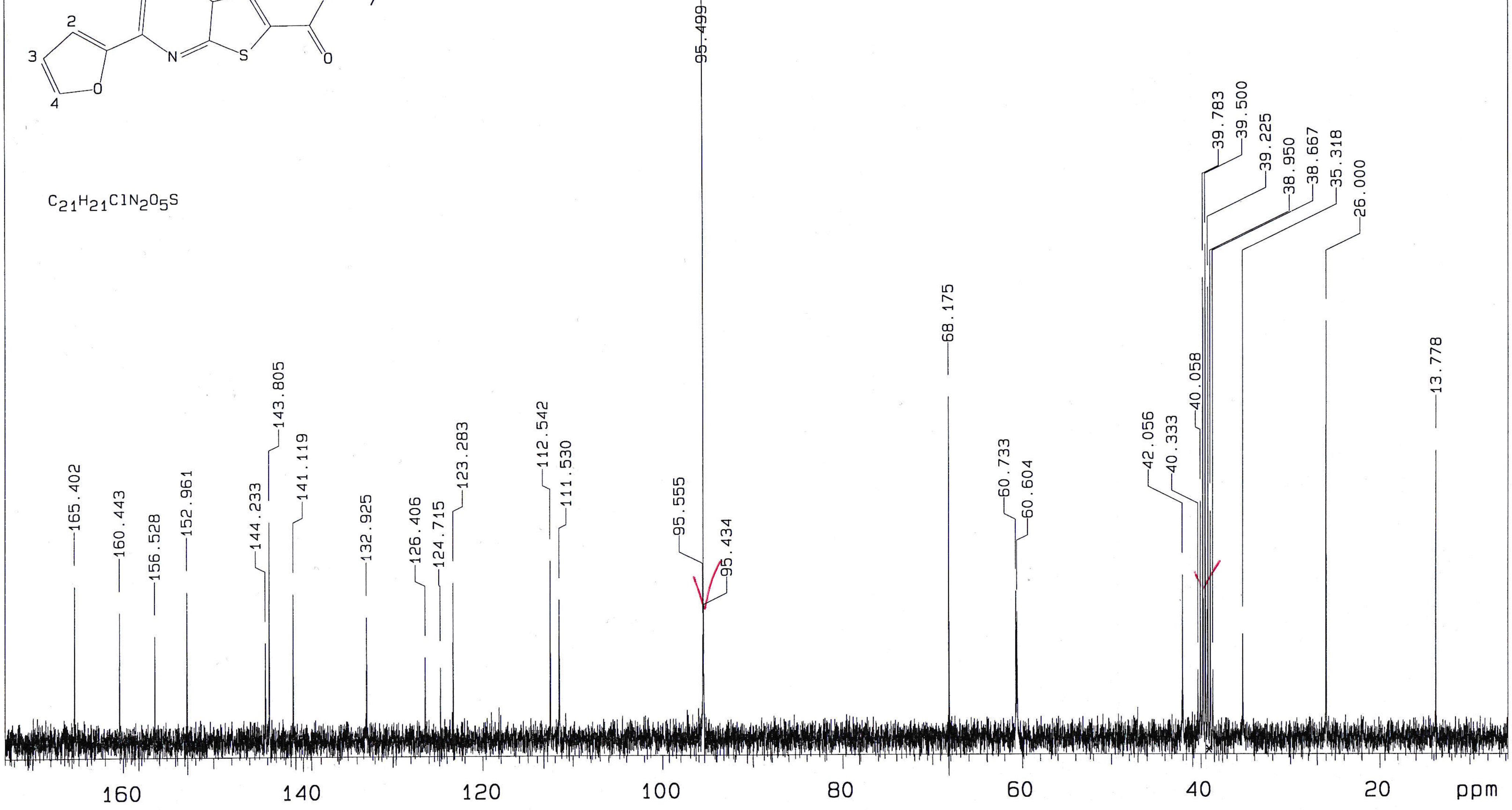

3a

Molecular Structure Research Centre, Yerevan, Armenia, Varian Mercury-300VX H1 300.088 MHz, nt=16, np=32000, temp=30.0 C, lb=-0.2, solvent=DMSO/CCl4 1/3 Nov 1 2023

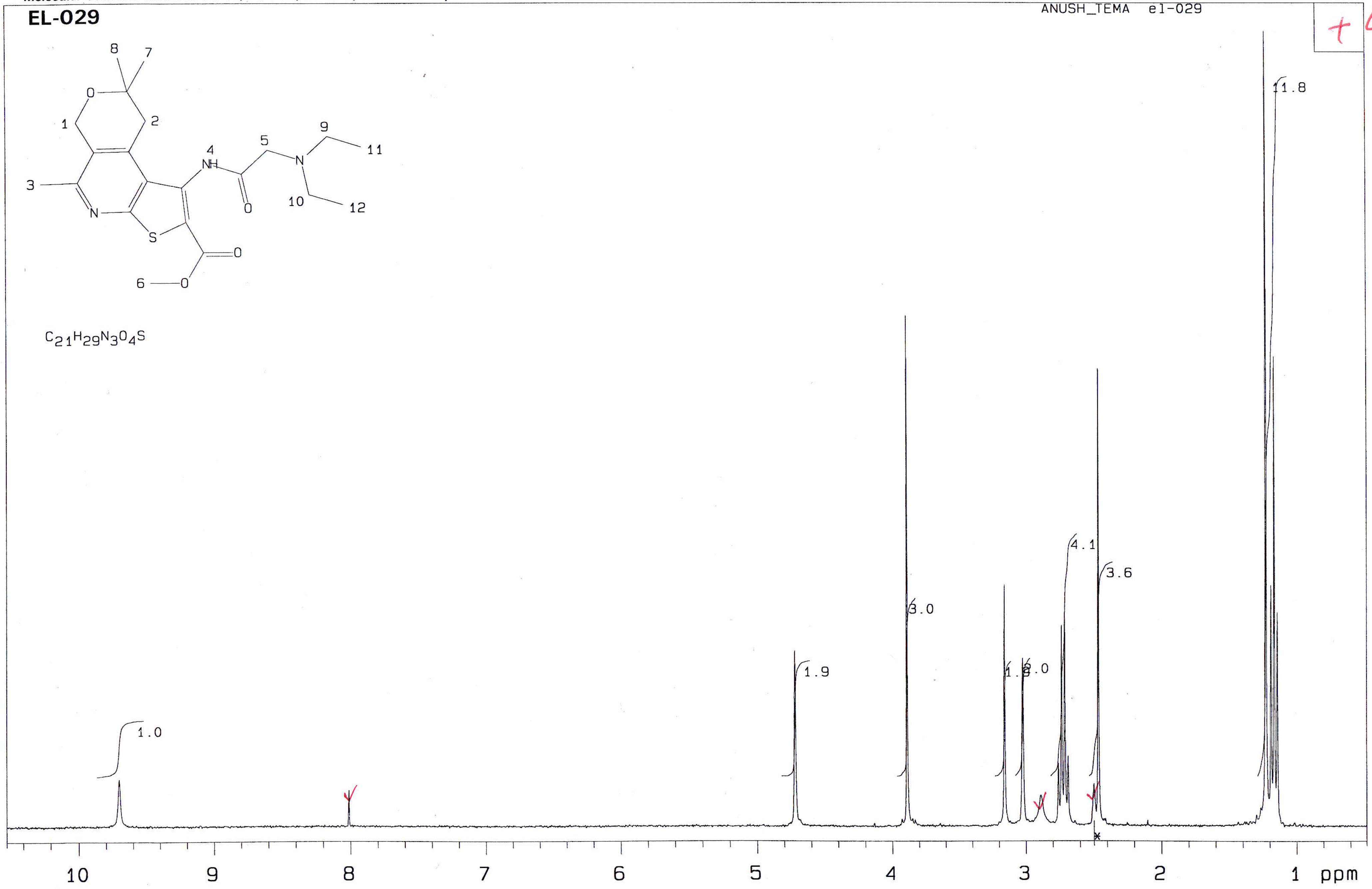

3a

Molecular Structure Research Centre, Yerevan, Armenia, Varian Mercury-300VX

C13 75.465 MHz, nt = 400, np = 19998, temp = 30.0 C, lb = 1.0, solvent = DMSO/CCl4 1/3

Nov 1 2023

ANUSH_TEMA e1-029

EL-029

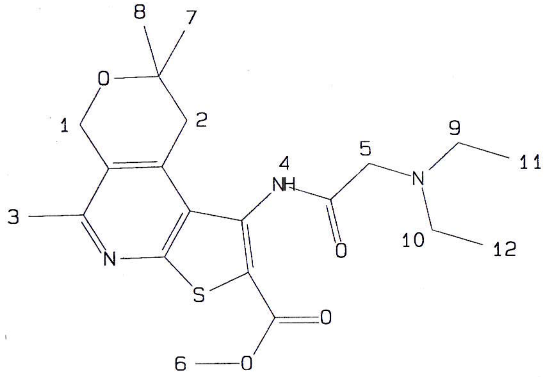

$C_{21}H_{29}N_3O_4S$

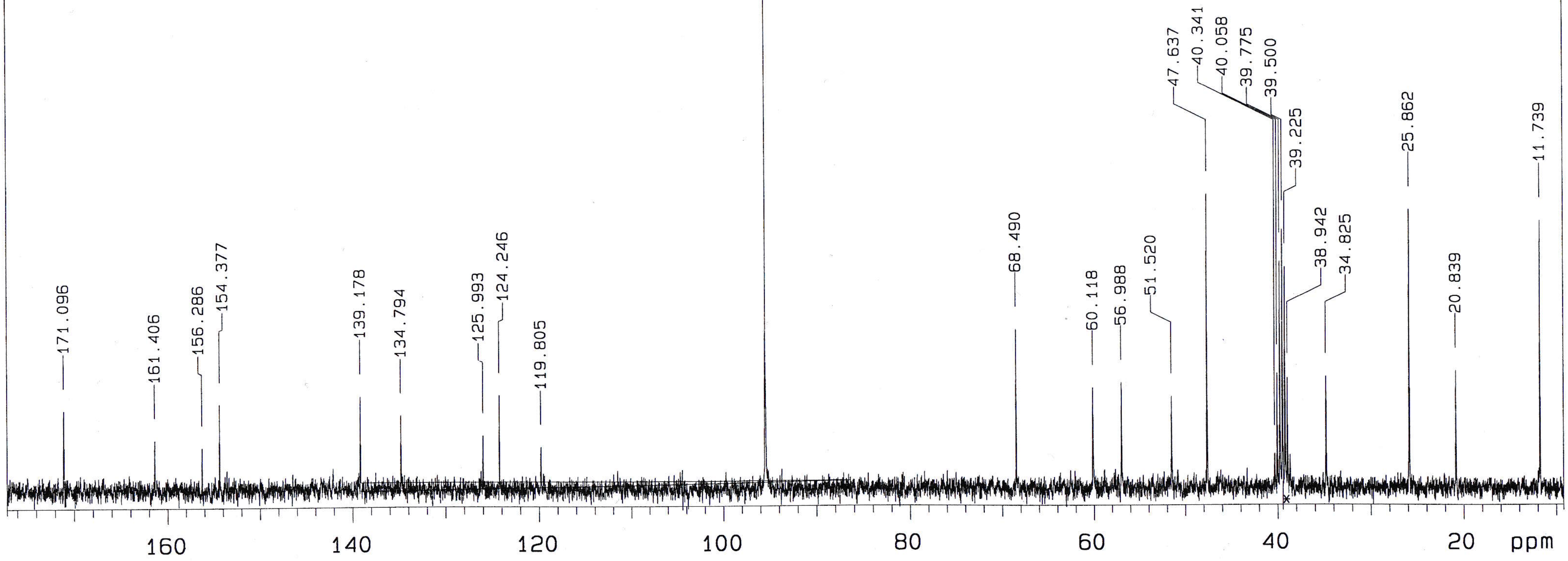

38

Molecular Structure Research Centre, Yerevan, Armenia, Varian Mercury-300VX

H1 300.088 MHz, nt=16, np=32000, temp=30.0 C, lb=-0.2, solvent=DMSO/CCl4 1/3

Nov 3 2023

ANUSH_TEMA el-034

EL-034

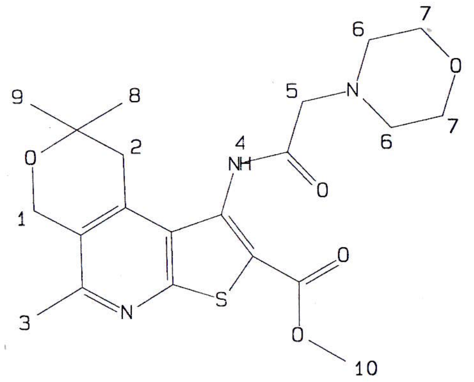

$C_{21}H_{27}N_3O_5S$

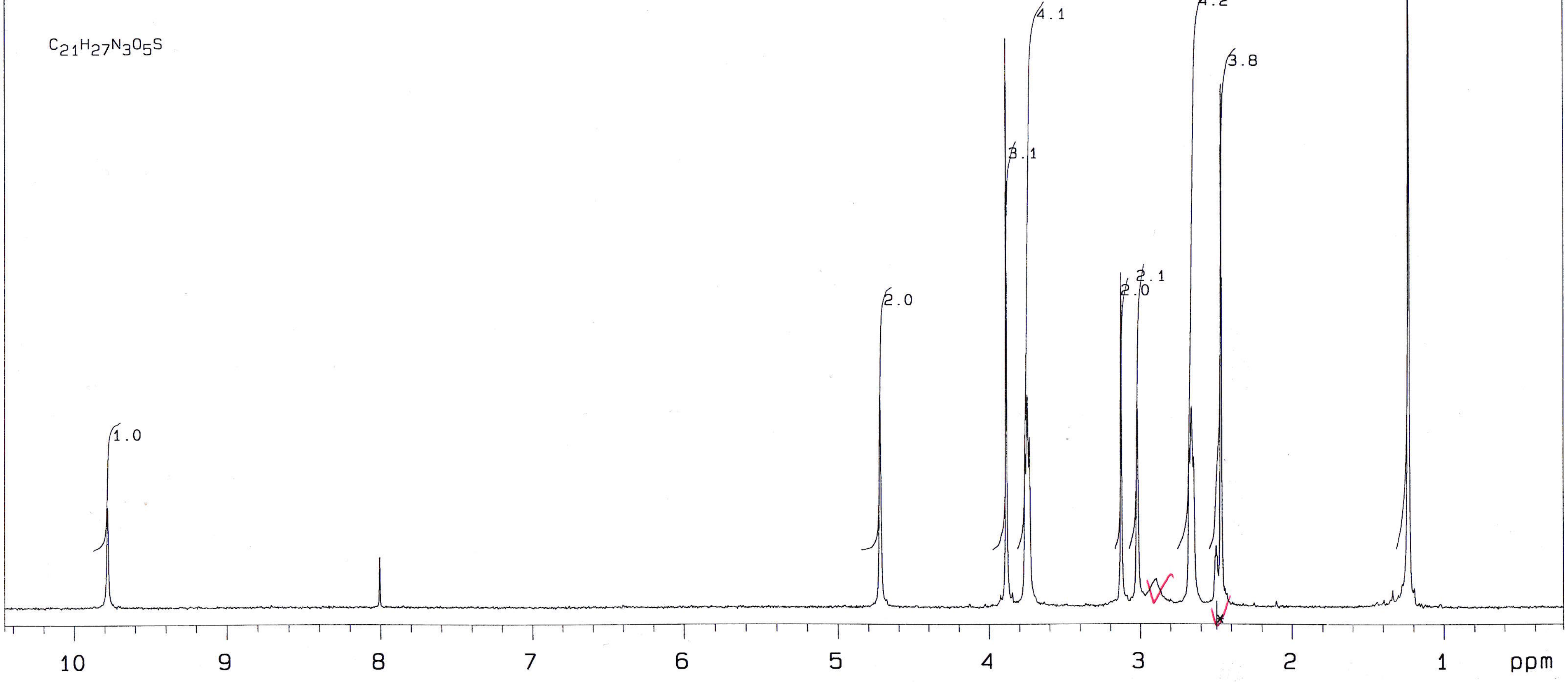

38

Molecular Structure Research Centre, Yerevan, Armenia, Varian Mercury-300VX

C13 75.465 MHz, nt=448, np=19998, temp=30.0 C, lb=1.0, solvent=DMSO/CCl4 1/3

Nov 3 2023

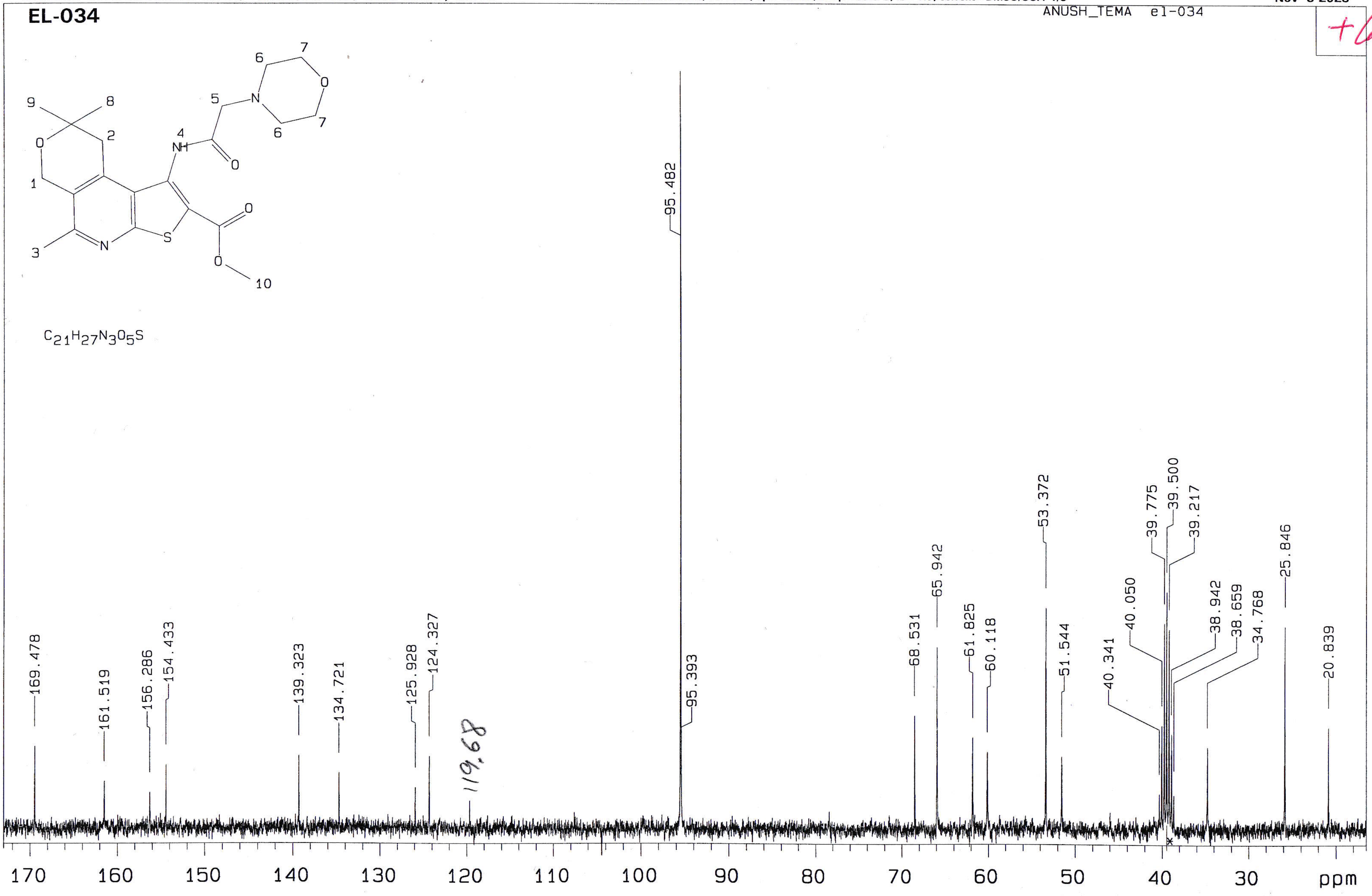

3c

Molecular Structure Research Centre, Yerevan, Armenia, Varian Mercury-300VX

H1 300.088 MHz, nt = 16, np = 32000, temp = 30.0 C, lb = -0.2, solvent = DMSO/CCl4 1/3

Jan 13 2020

TEM-067

NOCI_20 tem-067

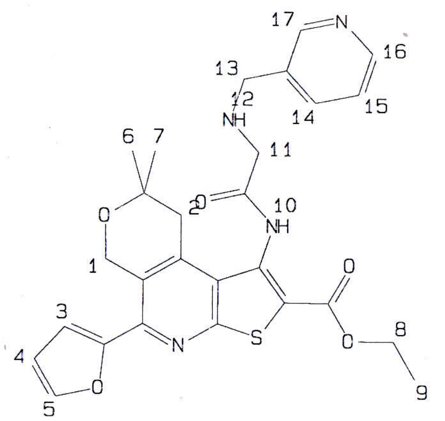

$C_{27}H_{28}N_4O_5S$

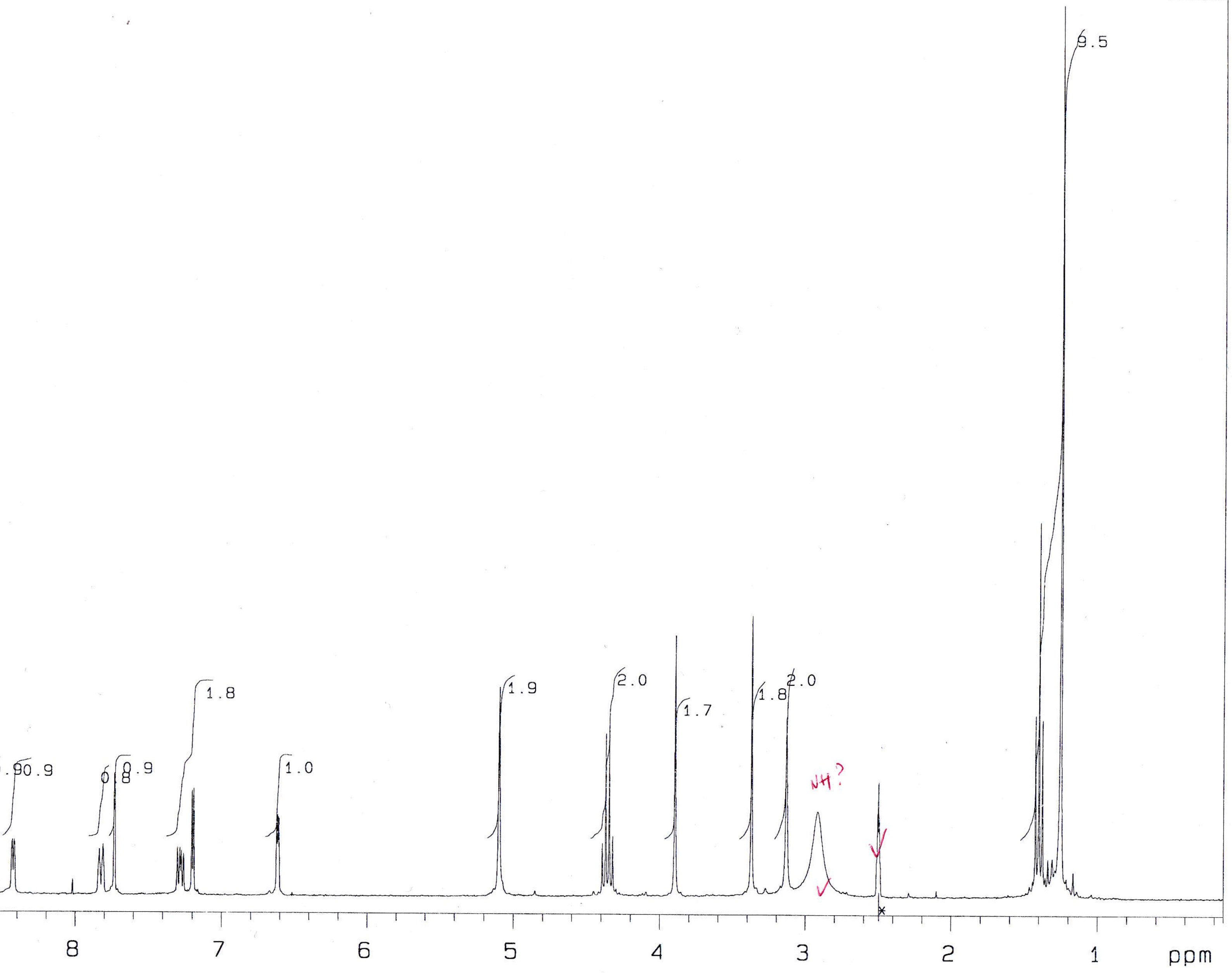

3c

Molecular Structure Research Centre, Yerevan, Armenia, Varian Mercury-300VX

C13 75.465 MHz, nt=848, np=19998, temp=30.0 C, lb=1.0, solvent=DMSO/CCl4 1/3

Jan 13 2020

TEM-067

NOCI_20 tem-067

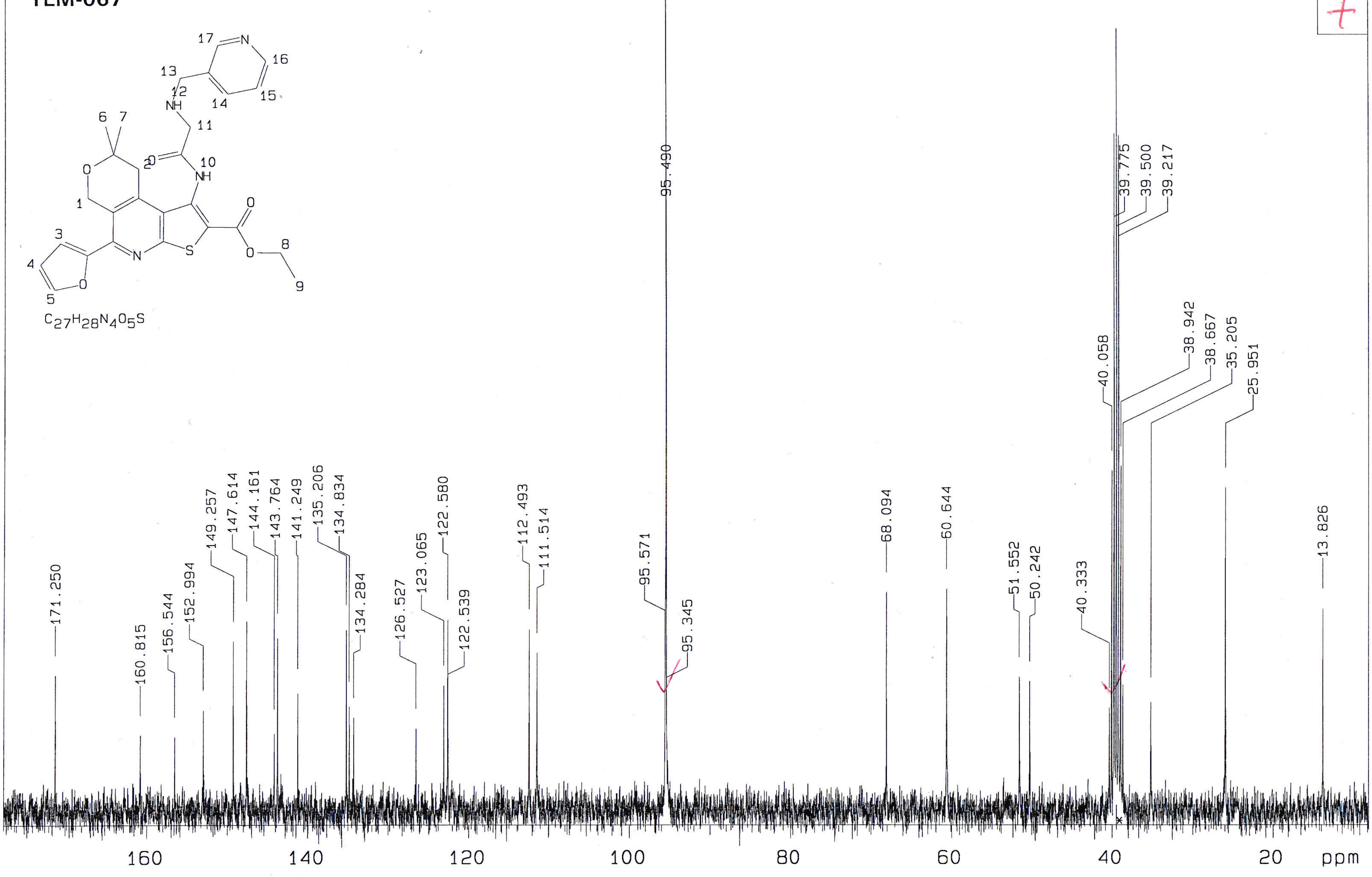

4a

Molecular Structure Research Centre, Yerevan, Armenia, Varian Mercury-300VX

H1 300.088 MHz, nt = 16, np = 32000, temp = 30.0 C, lb = -0.2, solvent = DMSO/CCl4 1/3

Nov 3 2023

EL-036

ANUSH_TEMA el-036

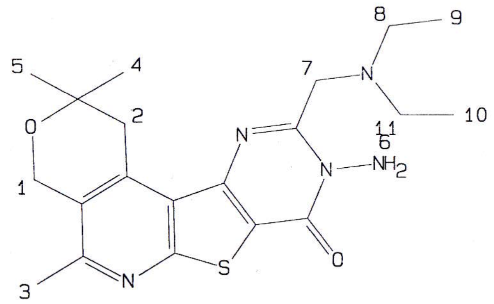

$C_{20}H_{27}N_5O_2S$

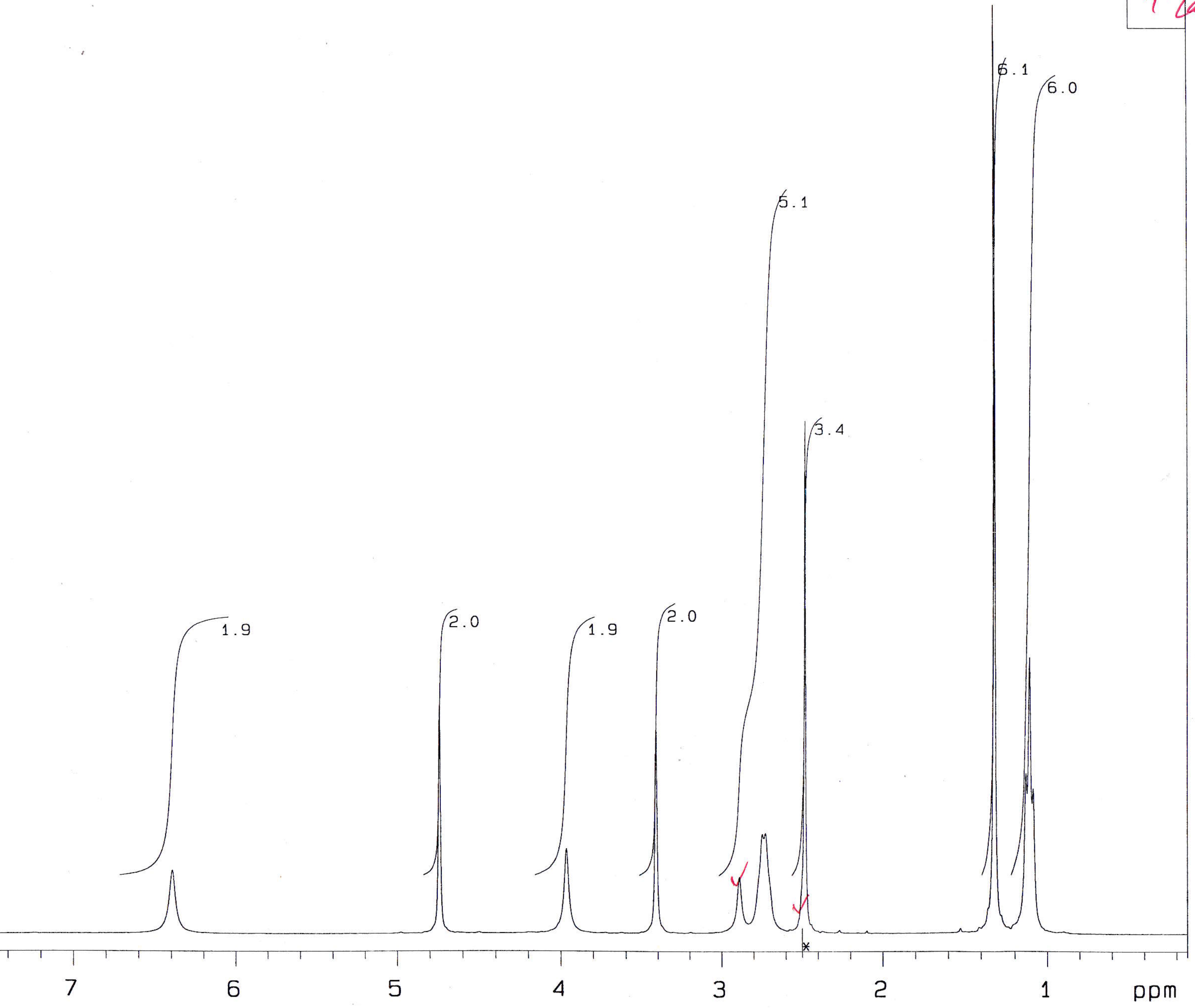

4a

Molecular Structure Research Centre, Yerevan, Armenia, Varian Mercury-300VX

C13 75.465 MHz, nt=512, np=19998, temp=30.0 C, lb=1.0, solvent=DMSO/CCl4 1/3

Nov 3 2023

ANUSH_TEMA e1-036

EL-036

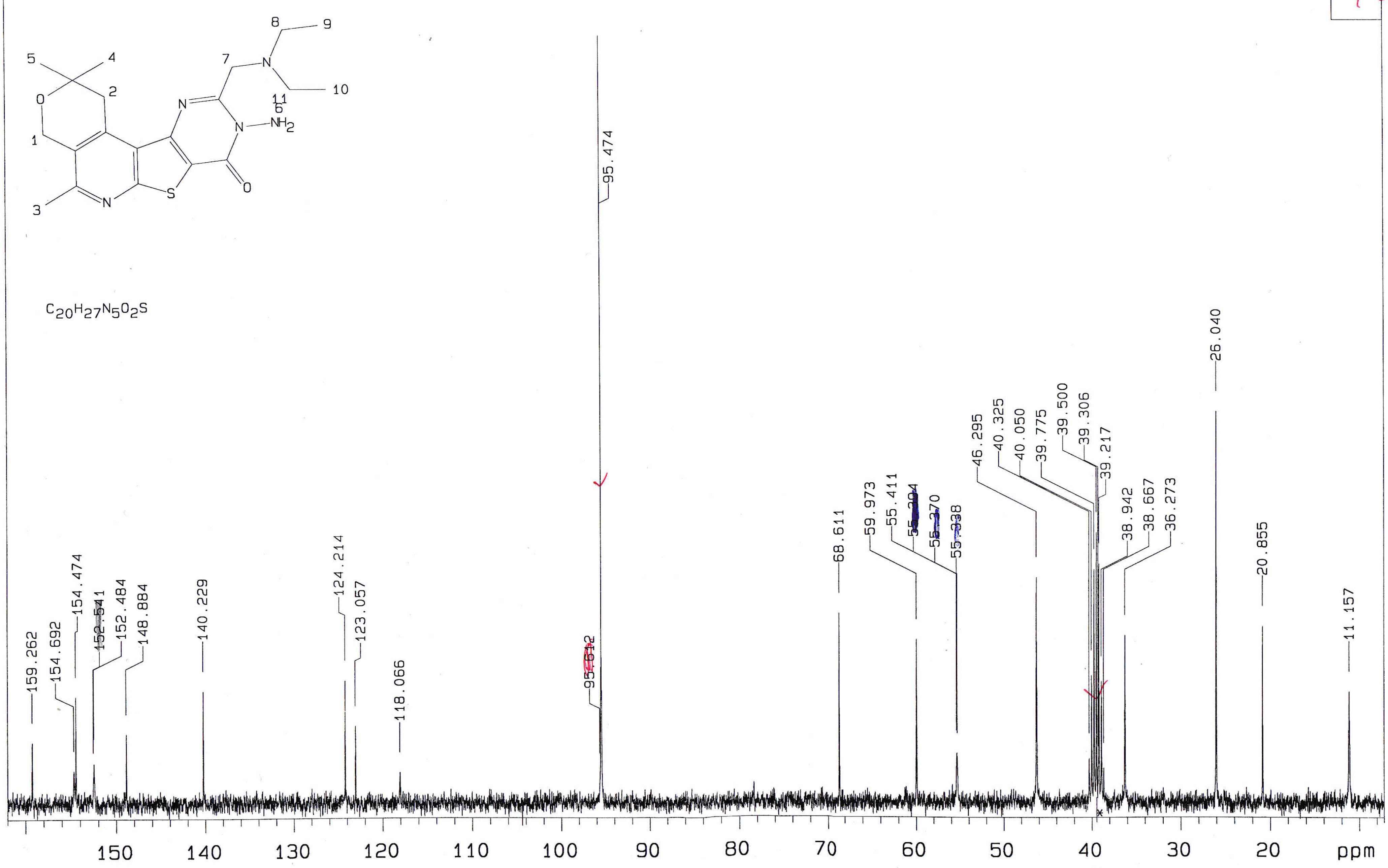

+

4B

Molecular Structure Research Centre, Yerevan, Armenia, Varian Mercury-300VX

H1 300.088 MHz, nt=16, np=32000, temp=30.0 C, lb=-0.2, solvent=DMSO/CCl4 ~~1/3~~ 1/1

Nov 8 2023

ANUSH_TEMA el-035

EL-035

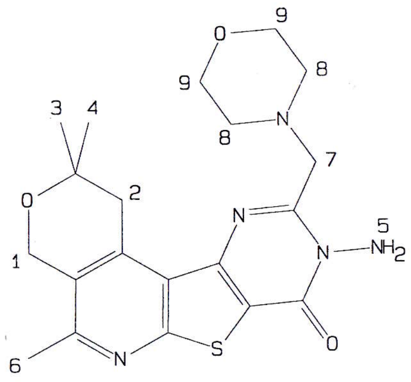

$C_{20}H_{25}N_{5}O_{3}S$

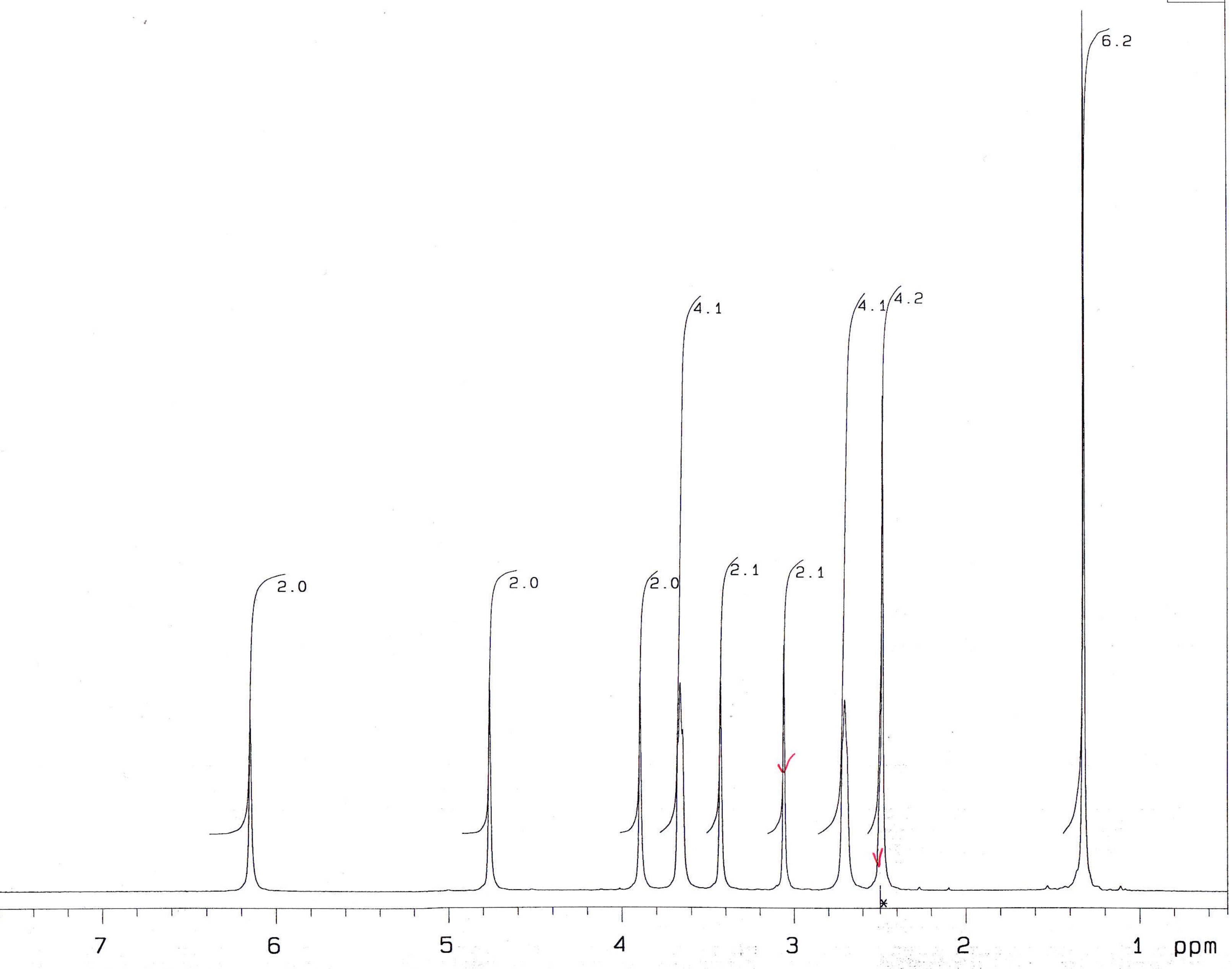

+

48

Molecular Structure Research Centre, Yerevan, Armenia, Varian Mercury-300VX C13 75.465 MHz, nt=416, np=19998, temp=30.0 C, lb=1.0, solvent=DMSO/CCl4 1/3 Nov 8 2023

EL-035

ANUSH_TEMA el-035

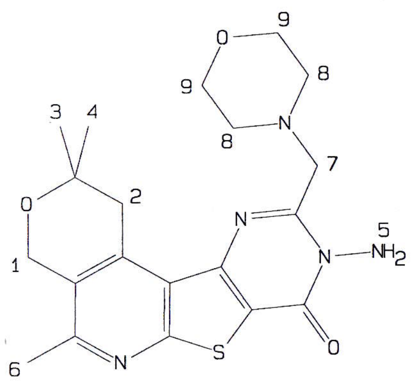

$C_{20}H_{25}N_5O_3S$

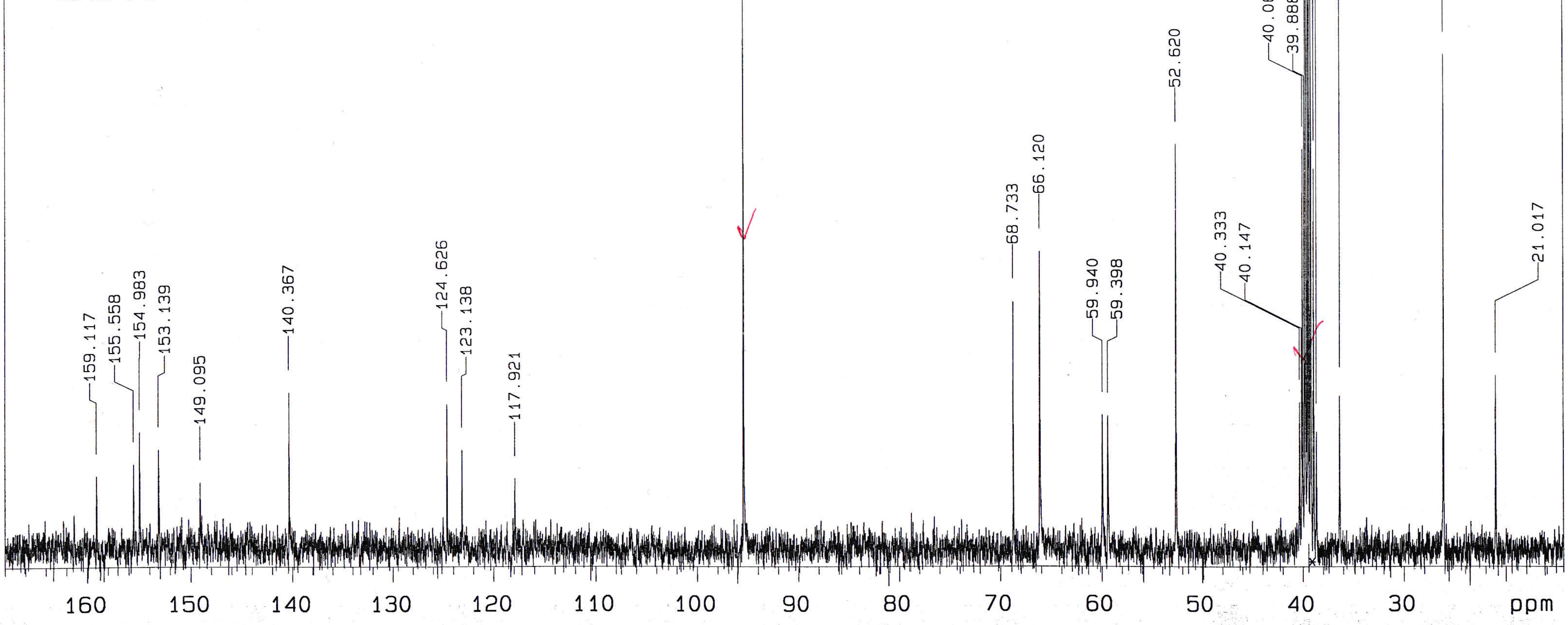

4 c

Molecular Structure Research Centre, Yerevan, Armenia, Varian Mercury-300VX H1 300.088 MHz, nt = 16, np = 32000, temp = 30.0 C, lb = -0.2, solvent = DMSO/CCl4 1/3 Jan 17 2020

TEM-070

SAMV_20 tem-070

+

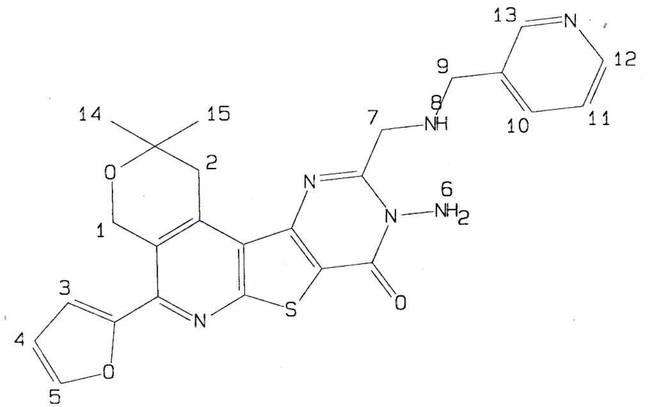

$C_{25}H_{24}N_6O_3S$

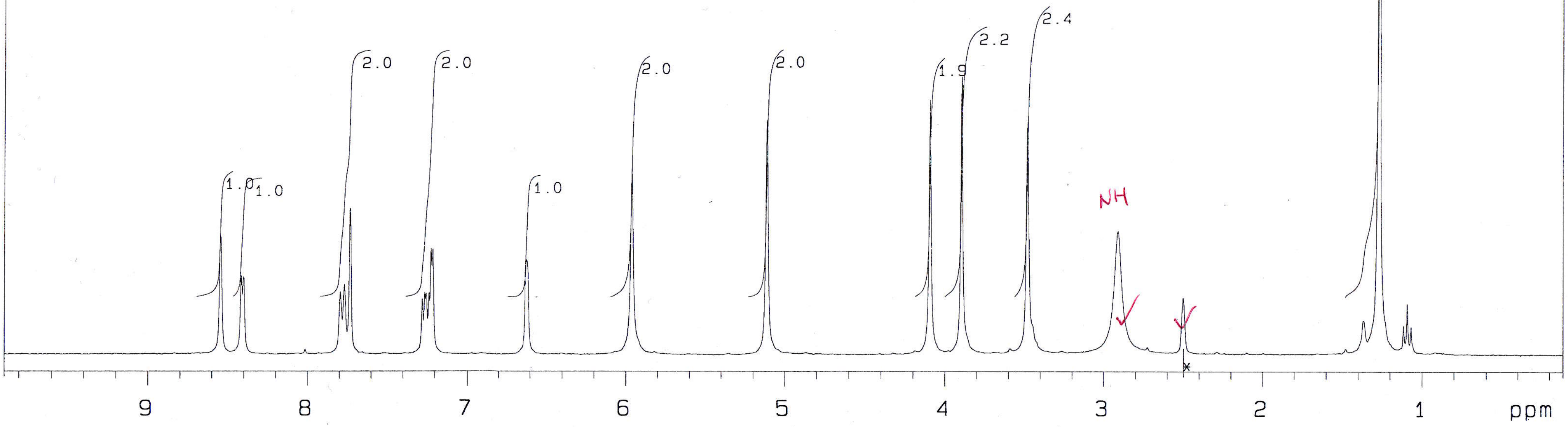

4c

Molecular Structure Research Centre, Yerevan, Armenia, Varian Mercury-300VX

C13 75.465 MHz, nt = 1200, np = 19998, temp = 30.0 C, lb = 1.0, solvent = DMSO/CCl4 1/3

Jan 17 2020

TEM-070

SAMV_20 tem-070

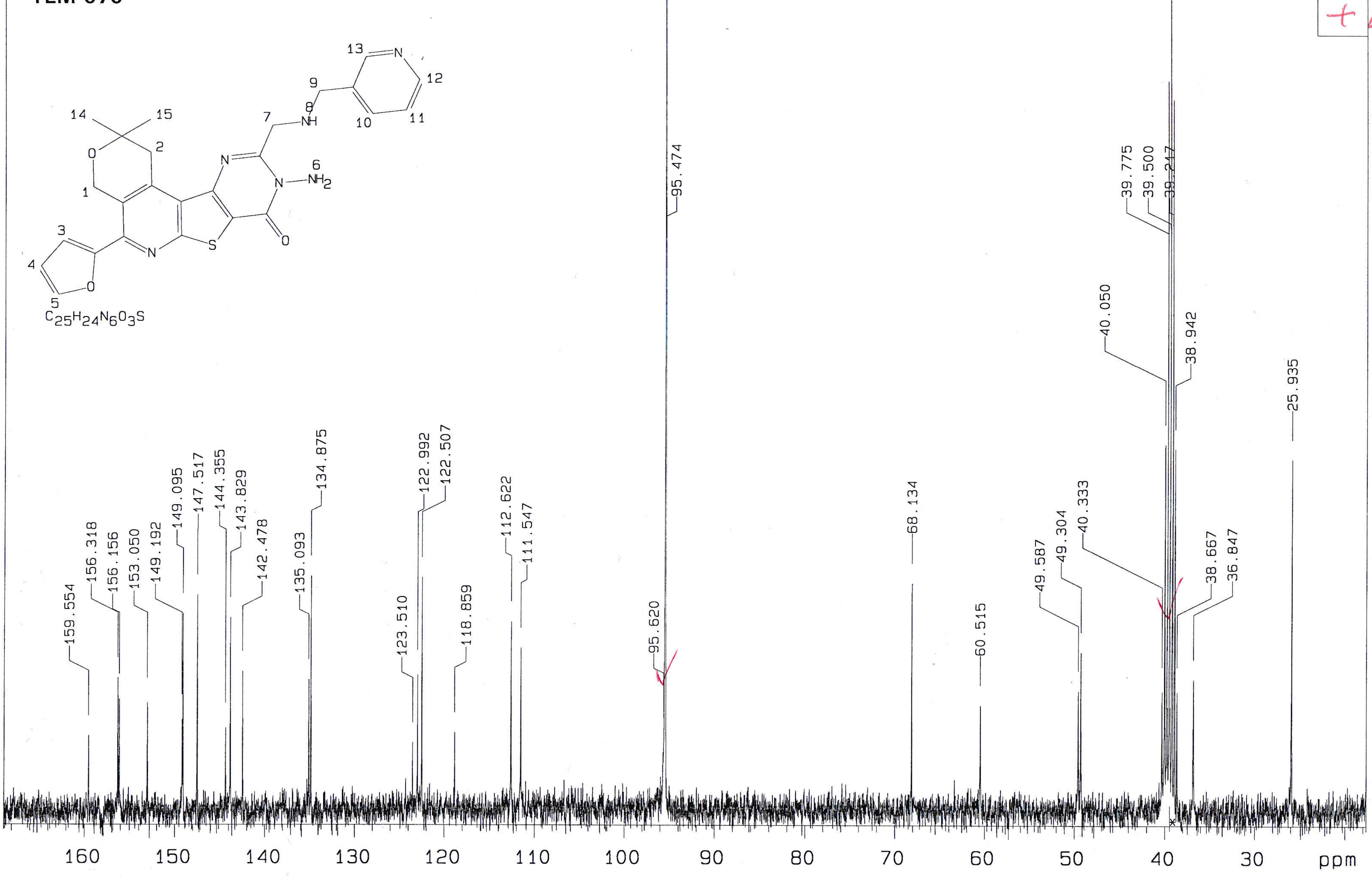

+

5

Molecular Structure Research Centre, Yerevan, Armenia, Varian Mercury-300VX

H1 300.088 MHz, nt = 16, np = 32000, temp = 30.0 C, lb = -0.2, solvent = DMSO/CCl4 1/3

Jan 30 2020

SAMV_20 tem-071

TEM-071

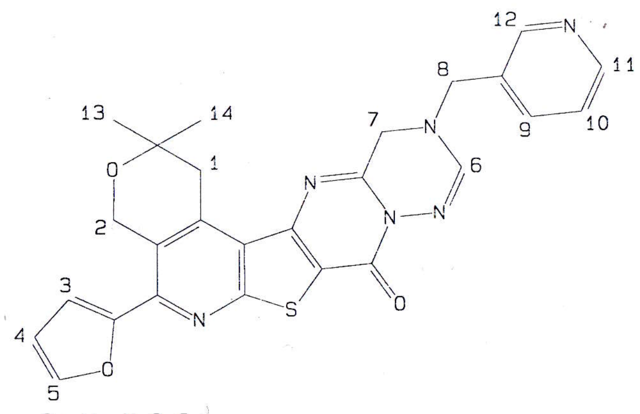

$C_{26}H_{22}N_6O_3S$

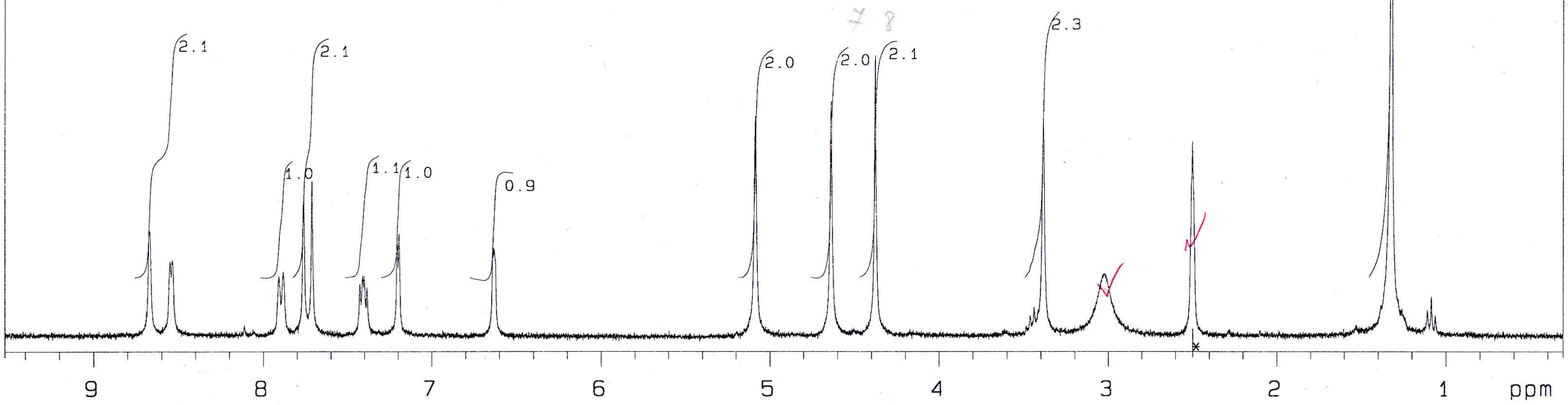

5

18.12.2023

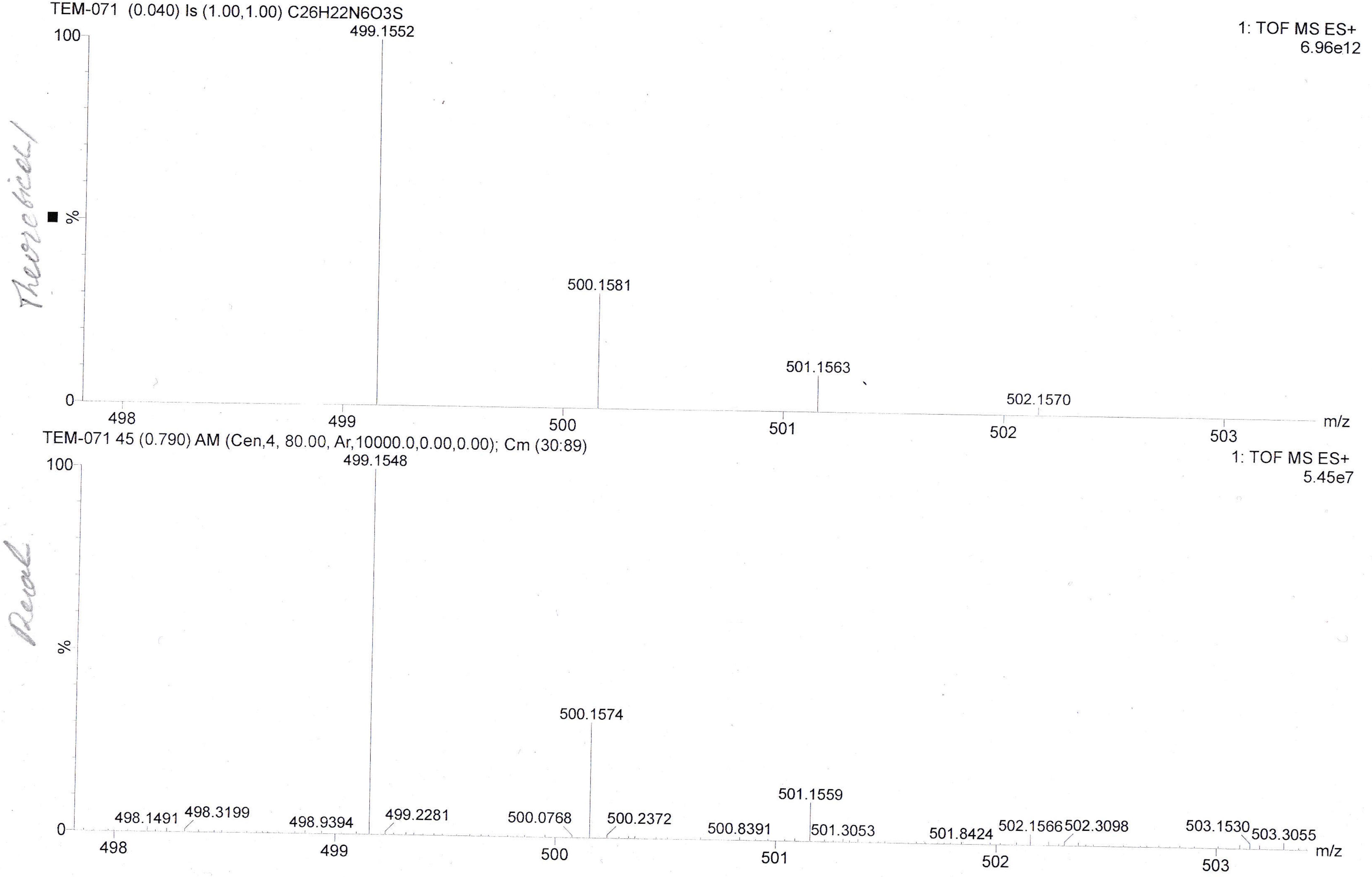

Theoretical
TEM-071 (0.040) Is (1.00,1.00) C26H22N6O3S
1: TOF MS ES+
6.96e12
100
%
0
499.1552
500.1581
501.1563
502.1570
498
499
500
501
502
503
m/z
Real
TEM-071 45 (0.790) AM (Cen,4, 80.00, Ar,10000.0,0.00,0.00); Cm (30:89)
1: TOF MS ES+
5.45e7
100
%
0
499.1548
500.1574
501.1559
498.1491
498.3199
498.9394
499.2281
500.0768
500.2372
500.8391
501.3053
501.8424
502.1566
502.3098
503.1530
503.3055
498
499
500
501
502
503
m/z

7a

Molecular Structure Research Centre, Yerevan, Armenia, Varian Mercury-300

H1 300.077 MHz, nt = 16, np = 16000, temp = 30.0 C, lb = -0.2, solvent = DMSO

May 26 2005

SAMV_05 s05-020

+

Inp. < 5%

S05-020

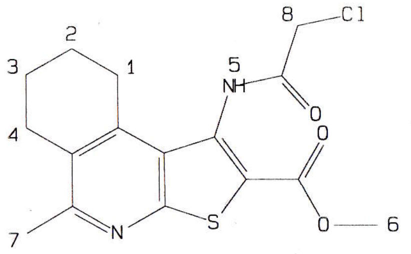

$C_{16}H_{17}ClN_2O_3S$

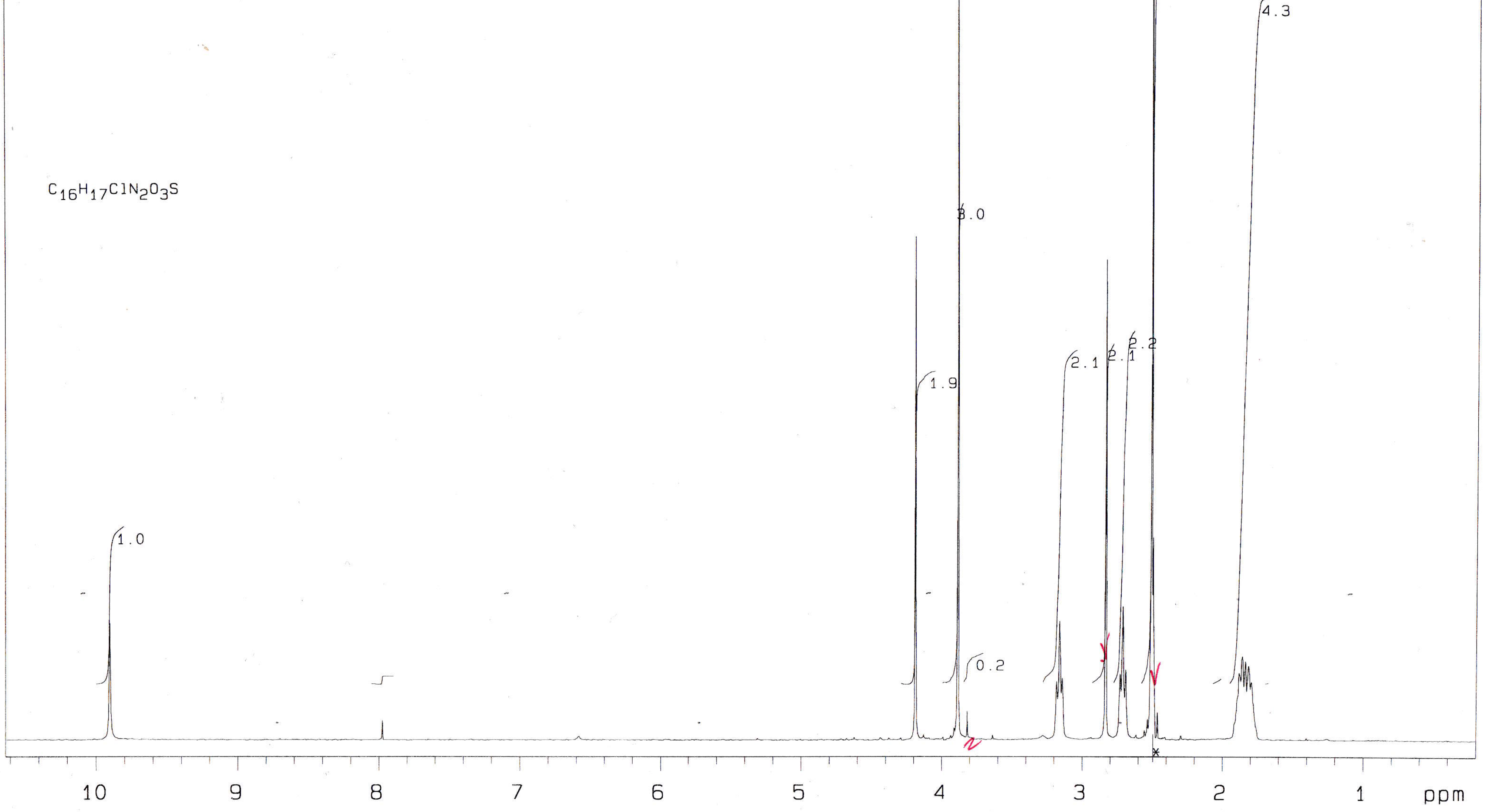

78

Molecular Structure Research Centre, Yerevan, Armenia, Varian Mercury-300VX

H1 300.088 MHz, nt = 16, np = 32000, temp = 30.0 C, lb = -0.2, solvent = DMSO/CCl4 1/3

Feb 6 2019

TEM-032

NOCI_19 tem-032

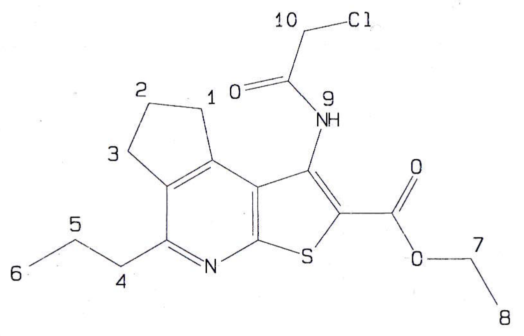

$C_{18}H_{21}ClN_2O_3S$

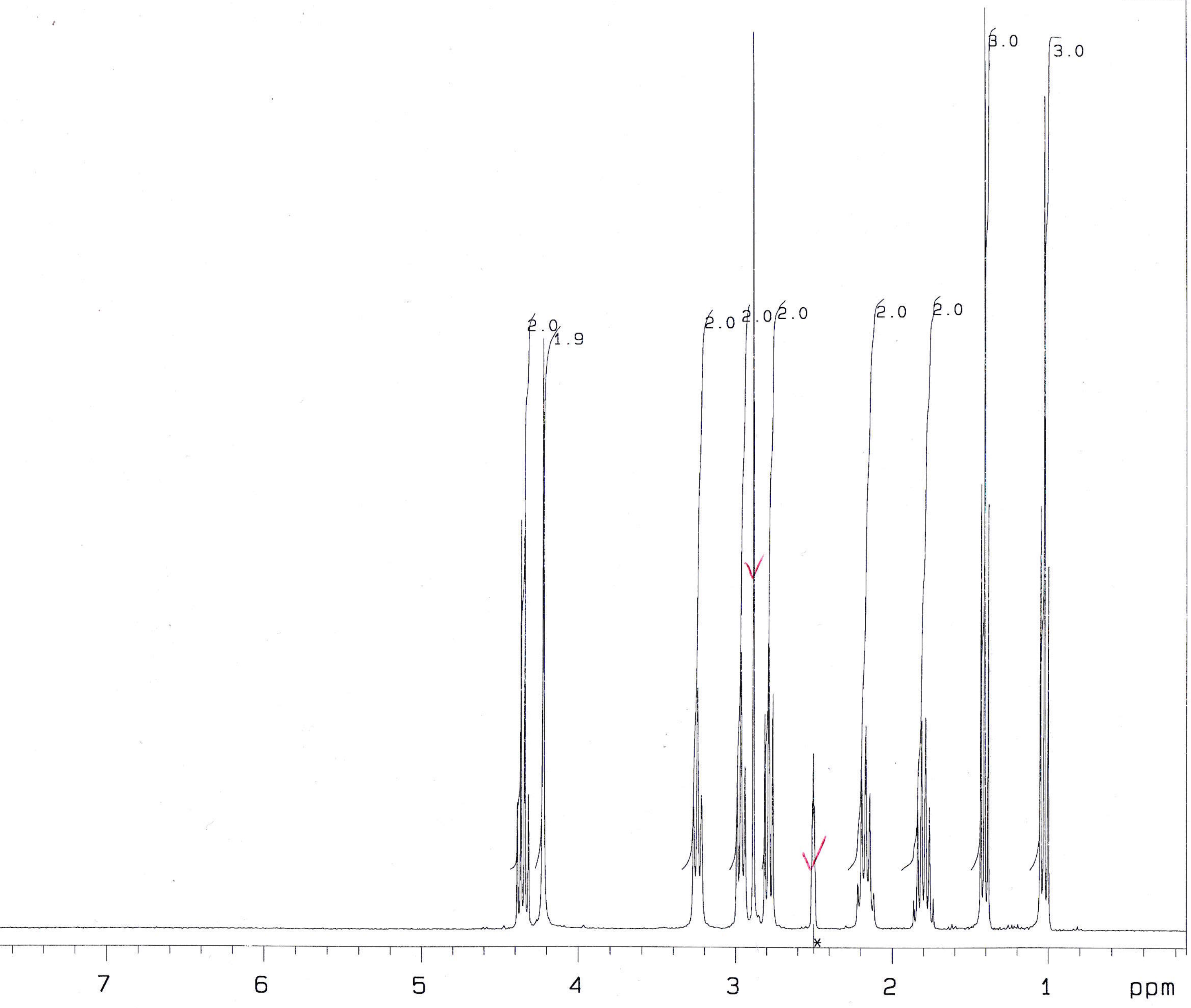

76

Molecular Structure Research Centre, Yerevan, Armenia, Varian Mercury-300VX

C13 75.465 MHz, nt=512, np=19998, temp=30.0 C, lb=1.0, solvent=DMSO/CCl4 1/3

Feb 6 2019

TEM-032

NOCI_19 tem-032

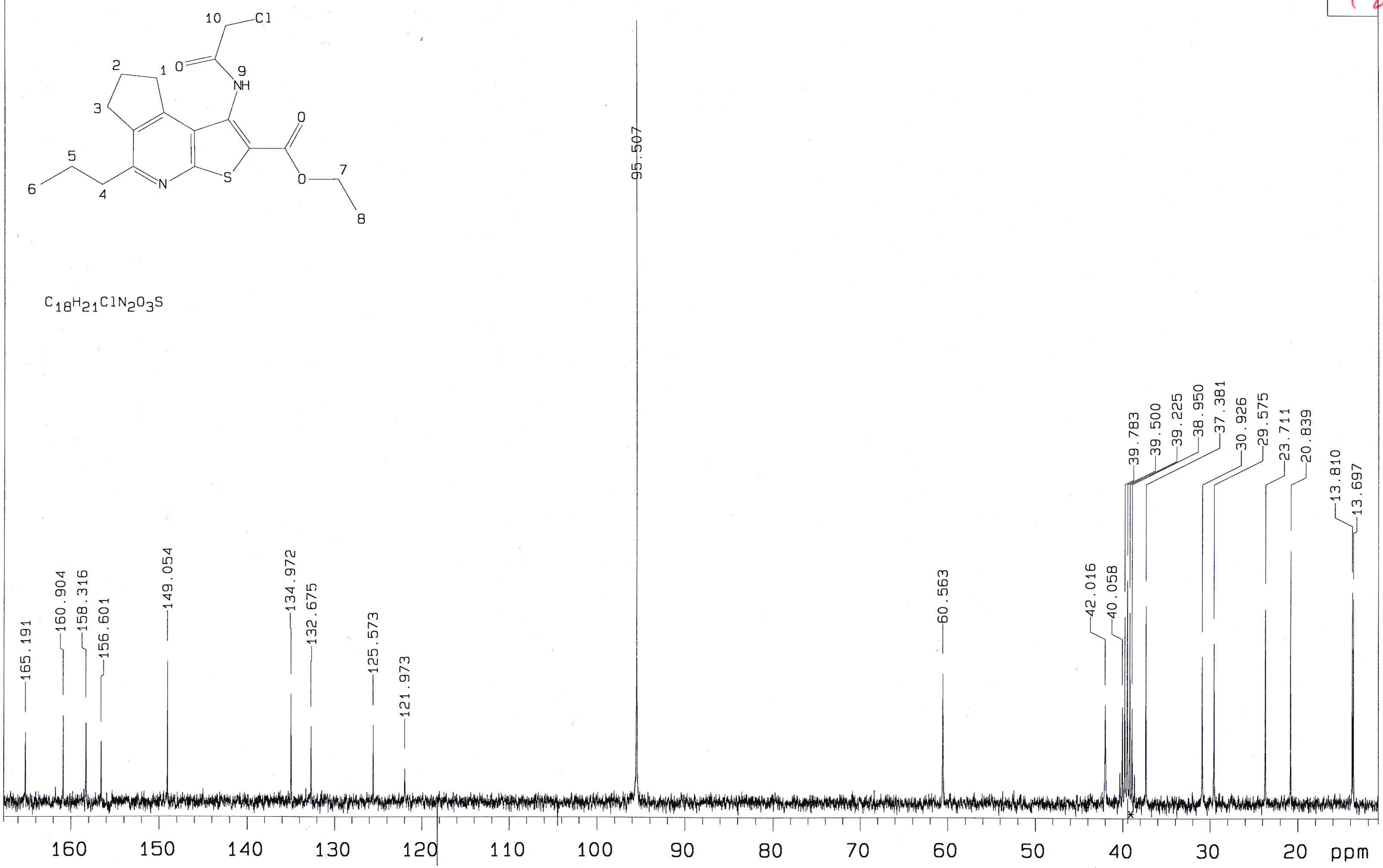

7c

Molecular Structure Research Centre, Yerevan, Armenia, Varian Mercury-300VX

H1 300.088 MHz, nt = 16, np = 32000, temp = 30.0 C, lb = -0.2, solvent = DMSO/CCl4 1/3

Oct 30 2023

ANUSH_TEMA el-021-2

EL-021-2

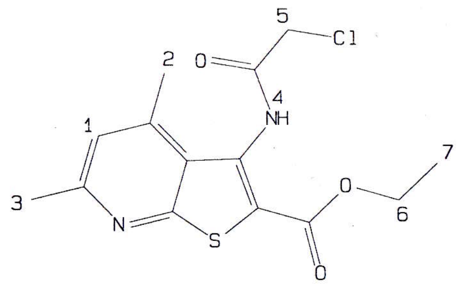

$C_{14}H_{15}ClN_2O_3S$

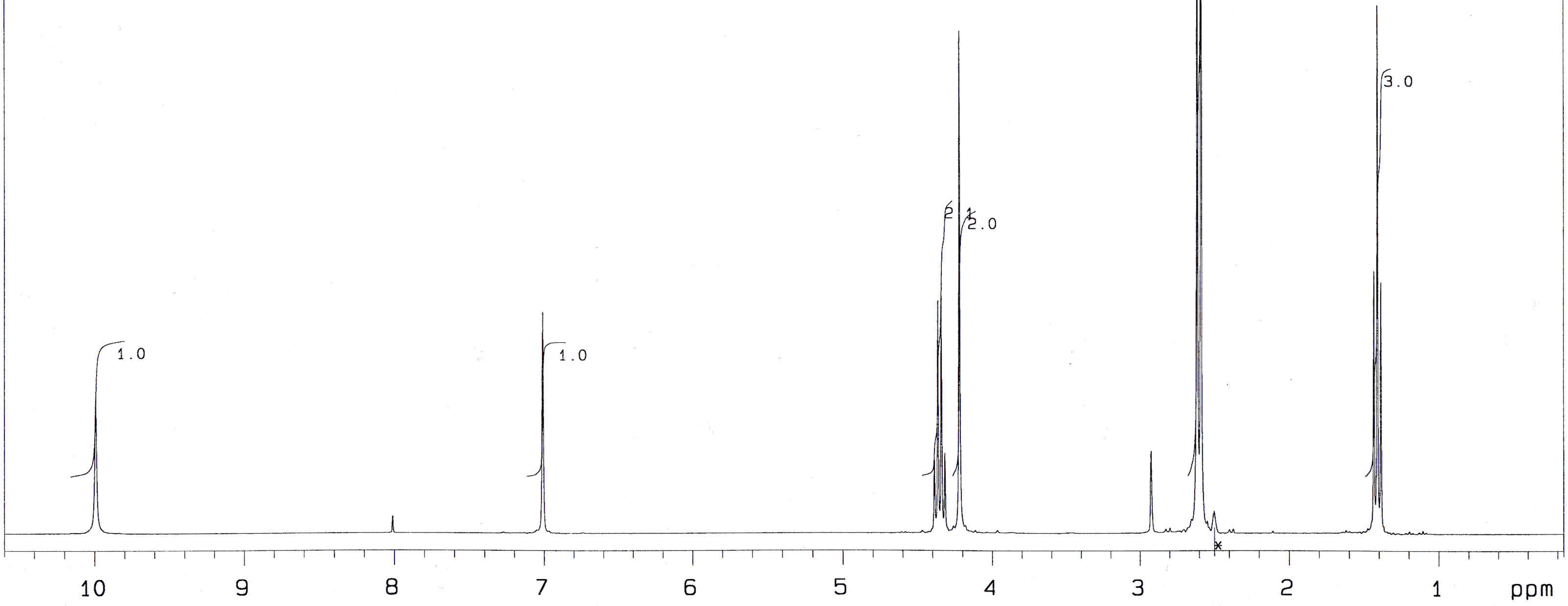

7C

Molecular Structure Research Centre, Yerevan, Armenia, Varian Mercury-300VX

C13 75.465 MHz, nt=96, np=19998, temp=30.0 C, lb=1.0, solvent=DMSO

Oct 30 2023

ANUSH_TEMA el-021-2

EL-021-2

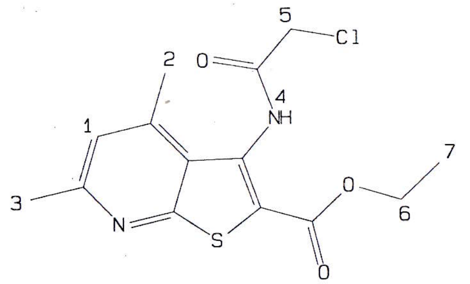

$C_{14}H_{15}ClN_2O_3S$

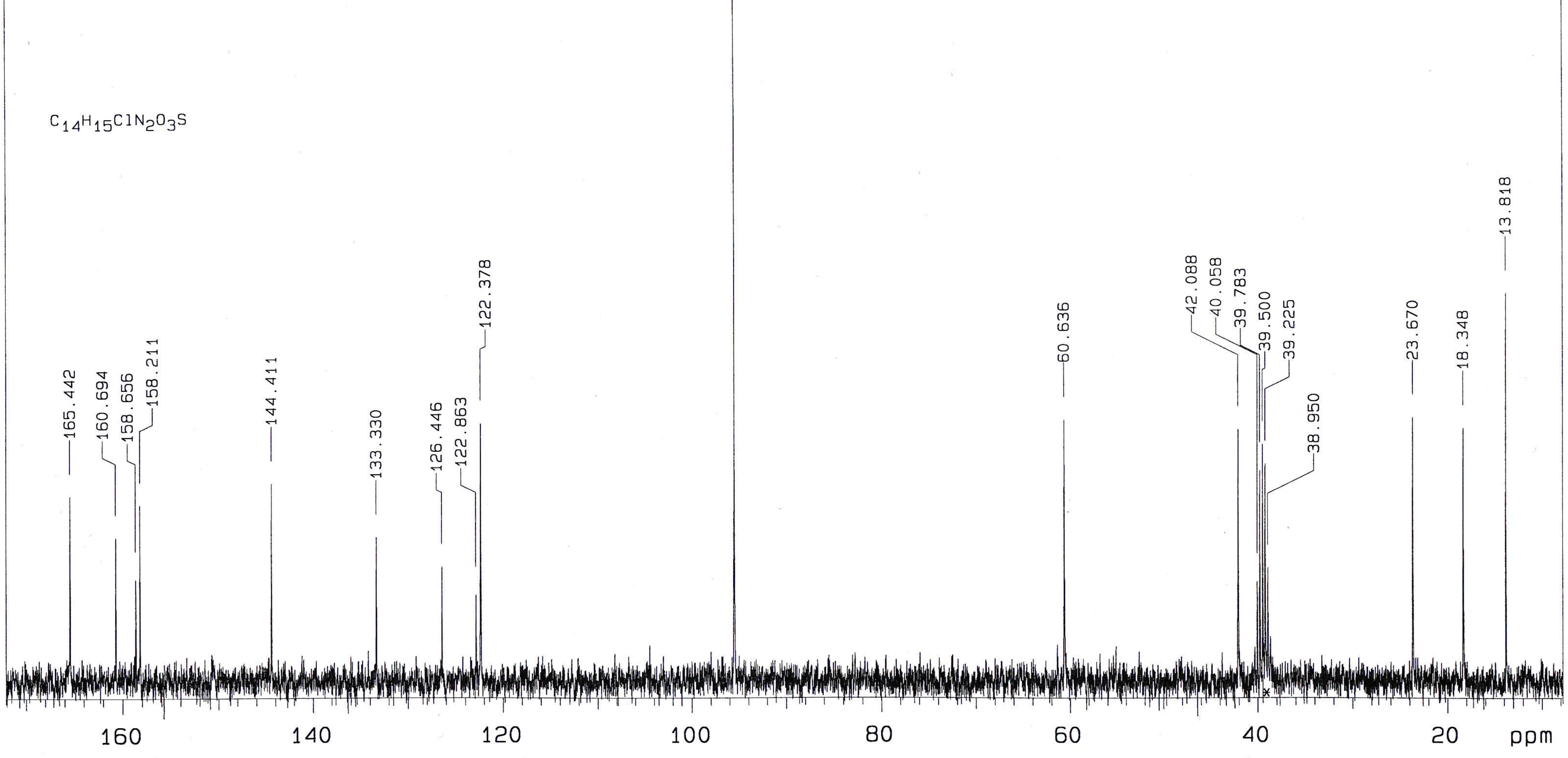

7d

Molecular Structure Research Centre, Yerevan, Armenia, Varian Mercury-300VX

H1 300.088 MHz, nt = 16, np = 32000, temp = 30.0 C, lb = -0.2, solvent = DMSO/CCl4 1/3

Feb 20 2024

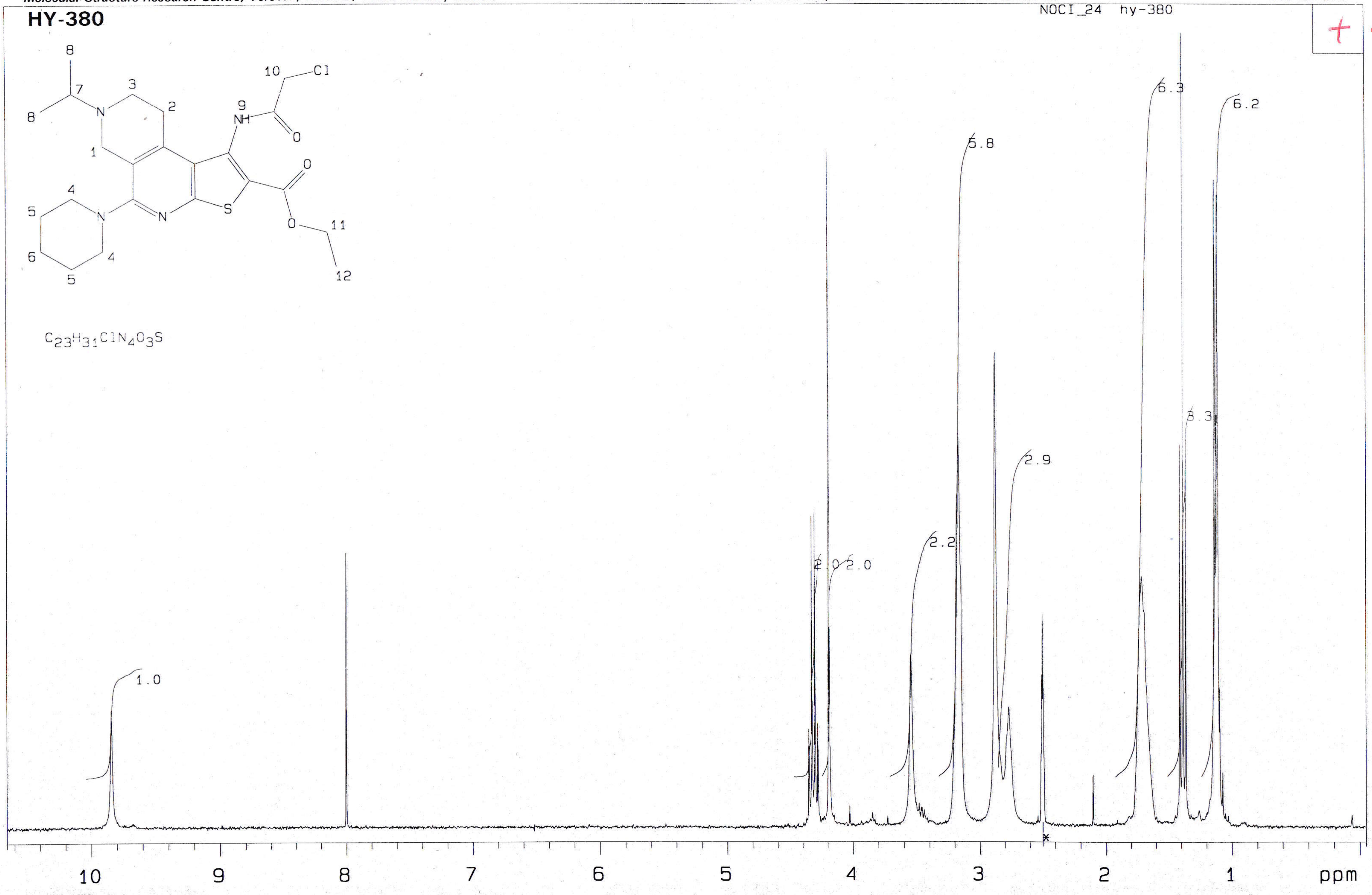

8a

Molecular Structure Research Centre, Yerevan, Armenia, Varian Mercury-300VX

H1 300.088 MHz, nt = 16, np = 32000, temp = 30.0 C, lb = -0.2, solvent = DMSO/CCl4 1/3

Nov 1 2023

ANUSH_TEMA e1-027

EL-027

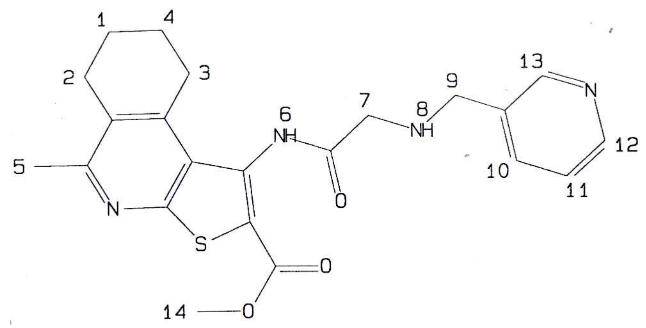

$C_{22}H_{24}N_4O_3S$

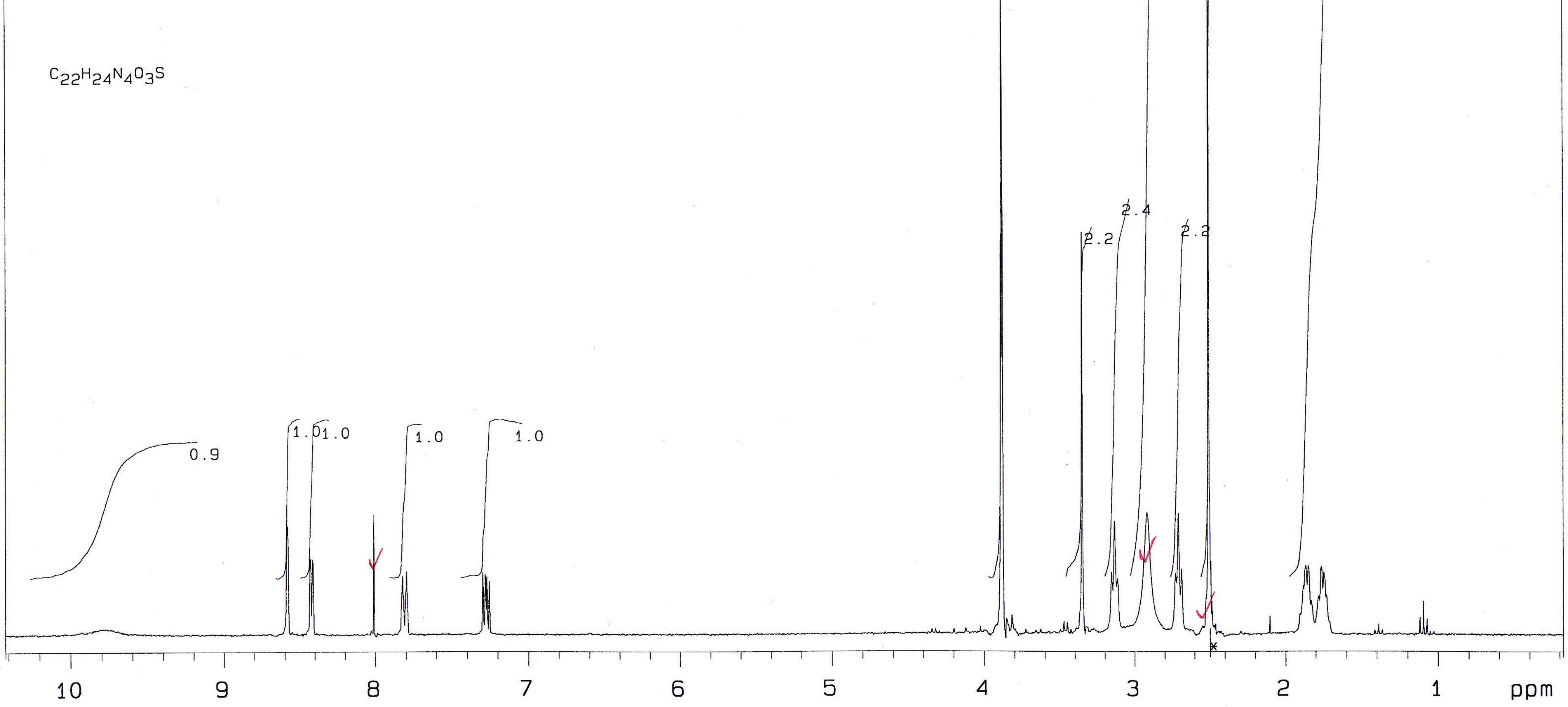

8a

Molecular Structure Research Centre, Yerevan, Armenia, Varian Mercury-300VX

C13 75.465 MHz, nt = 864, np = 19998, temp = 30.0 C, lb = 1.0, solvent = DMSO/CCl4 1/3

Nov 1 2023

ANUSH_TEMA el-027

EL-027

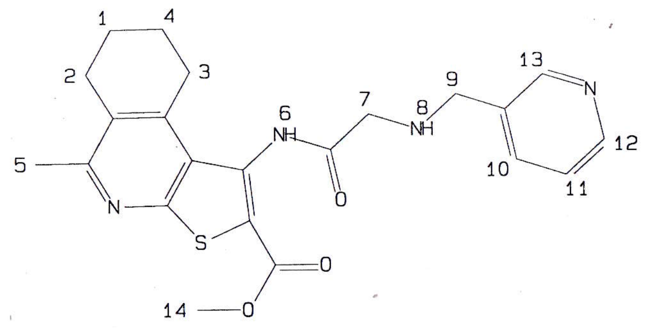

$C_{22}H_{24}N_4O_3S$

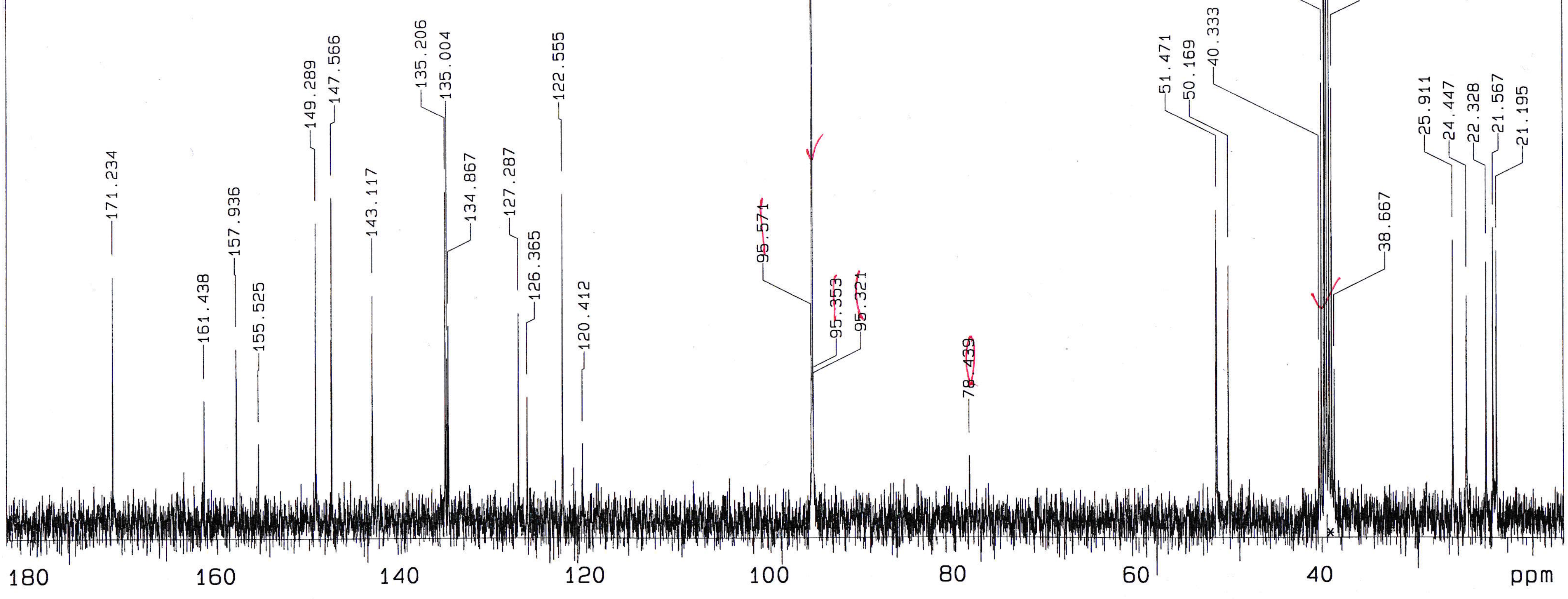

8б

Molecular Structure Research Centre, Yerevan, Armenia, Varian Mercury-300VX

H1 300.088 MHz, nt = 16, np = 32000, temp = 30.0 C, lb = -0.2, solvent = DMSO/CCl4 1/3

Feb 27 2019

TEM-037

SAMV_19 tem-037

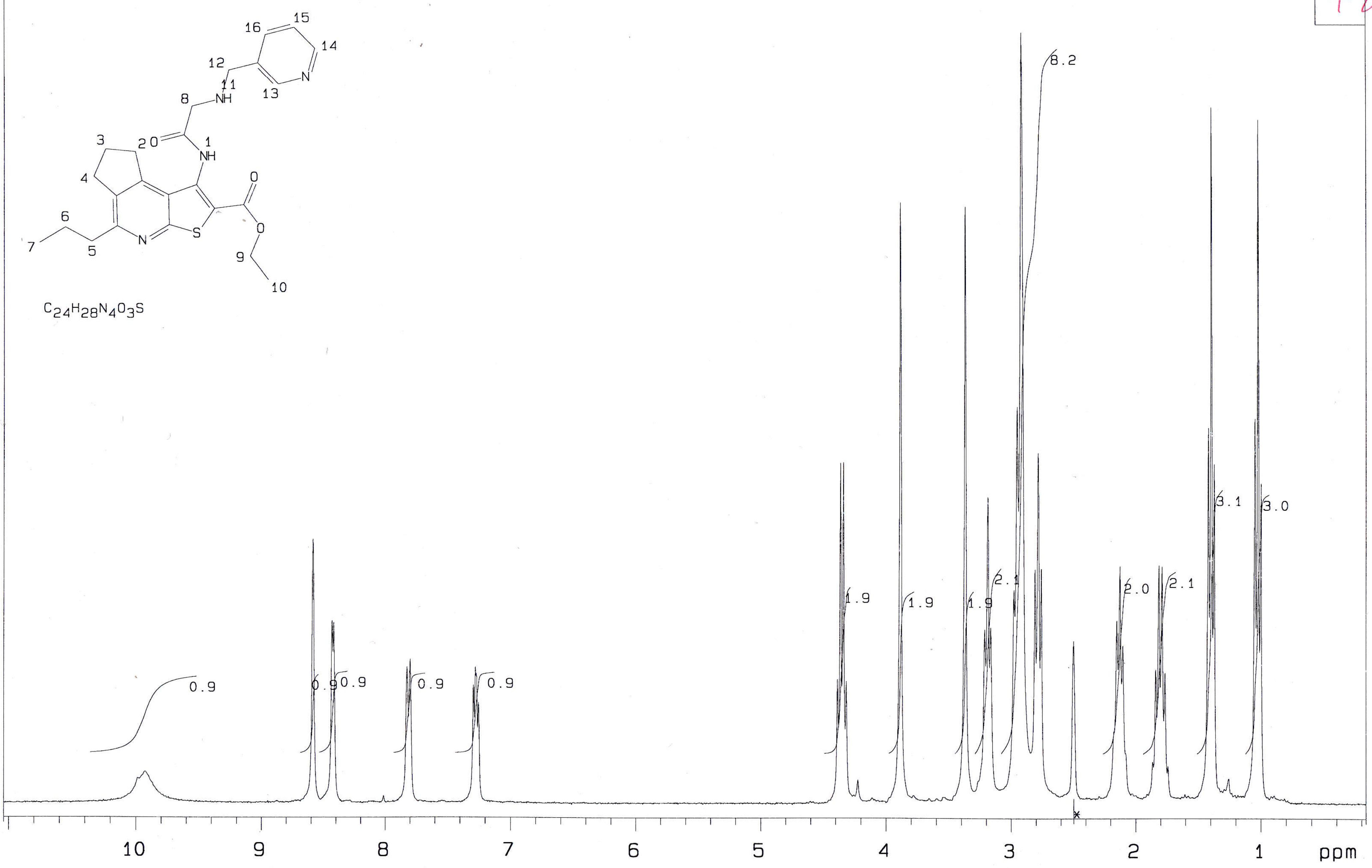

88

Molecular Structure Research Centre, Yerevan, Armenia, Varian Mercury-300VX

C13 75.465 MHz, nt=768, np=19998, temp=30.0 C, lb=1.0, solvent=DMSO/CCl4 1/3

Feb 27 2019

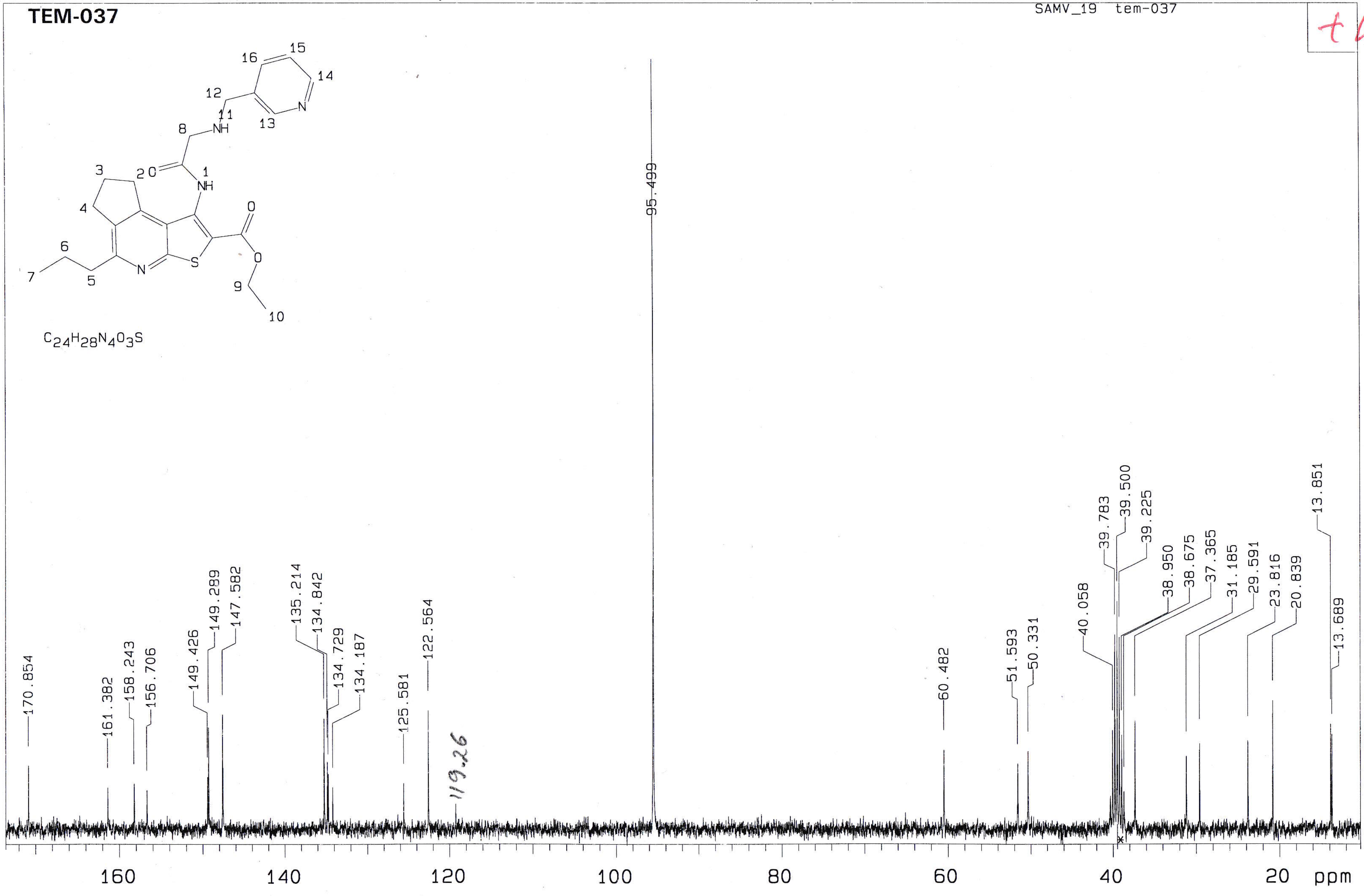

8c

Molecular Structure Research Centre, Yerevan, Armenia, Varian Mercury-300VX

H1 300.088 MHz, nt = 16, np = 32000, temp = 30.0 C, lb = -0.2, solvent = DMSO/CCl4 1/3

Nov 16 2023

ANUSH_TEMA e1-030-1

EL-030-1

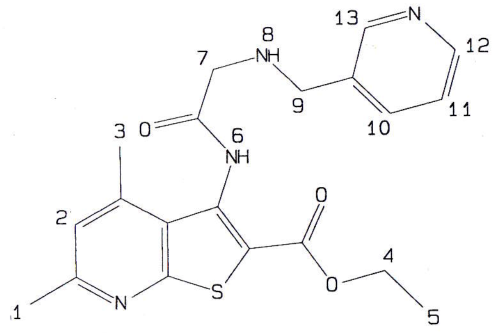

$C_{20}H_{22}N_4O_3S$

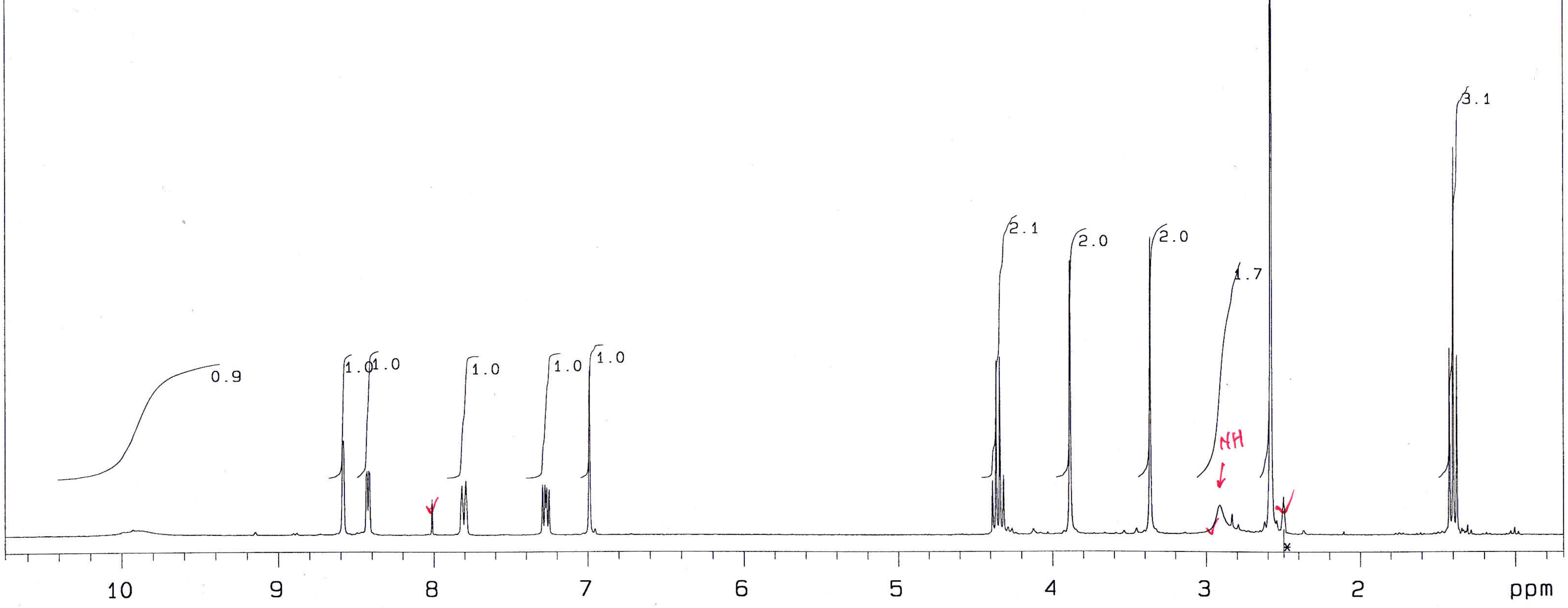

8c

Molecular Structure Research Centre, Yerevan, Armenia, Varian Mercury-300VX C13 75.465 MHz, nt=448, np=19998, temp=30.0 C, lb=1.0, solvent=DMSO/CCl4 1/3 Nov 16 2023

ANUSH_TEMA e1-030-1

EL-030-1

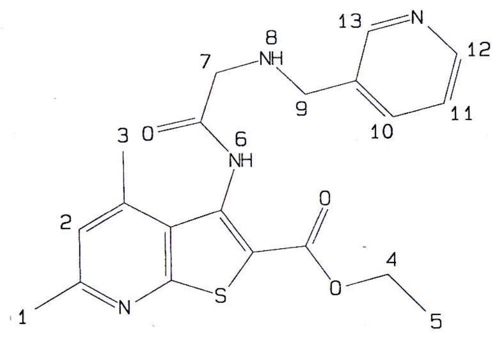

$C_{20}H_{22}N_4O_3S$

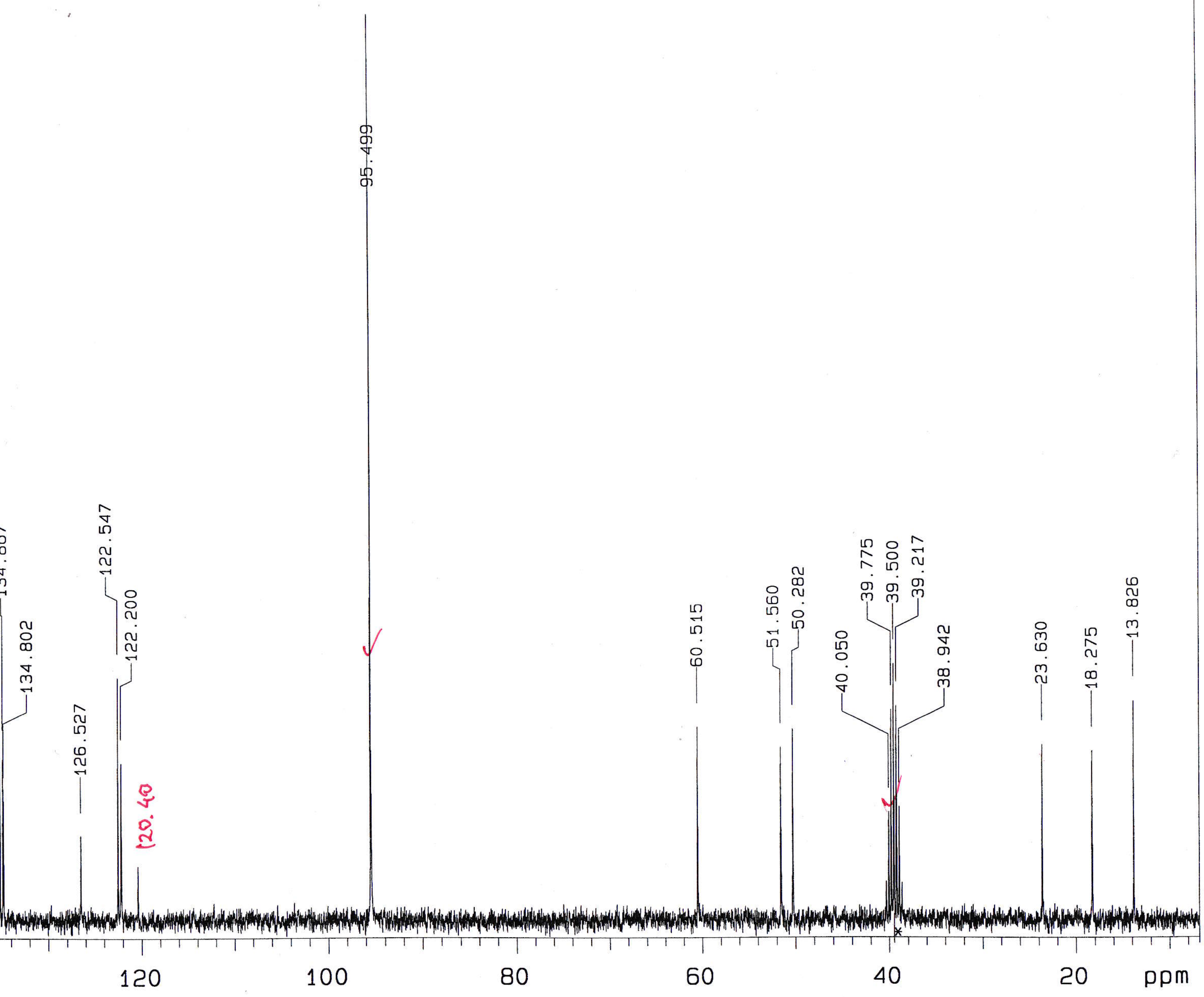

9a

Molecular Structure Research Centre, Yerevan, Armenia, Varian Mercury-300VX

H1 300.088 MHz, nt = 16, np = 32000, temp = 30.0 C, lb = -0.2, solvent = DMSO/CCl4 1/3

Jul 26 2017

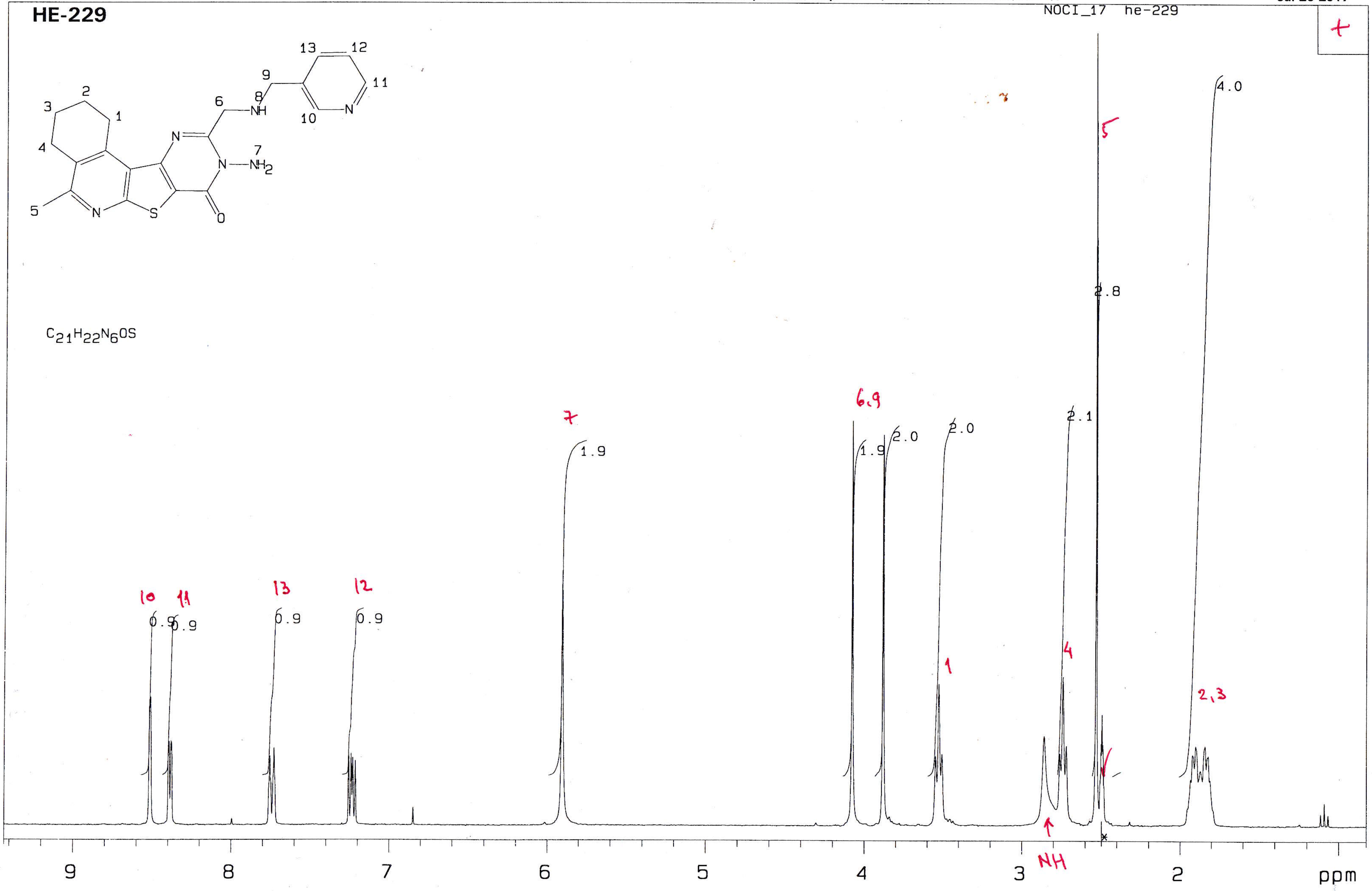

9a

Molecular Structure Research Centre, Yerevan, Armenia, Varian Mercury-300VX

C13 75.465 MHz, nt = 1280, np = 19998, temp = 30.0 C, lb = 1.0, solvent = DMSO/CCl4 1/3

Jul 26 2017

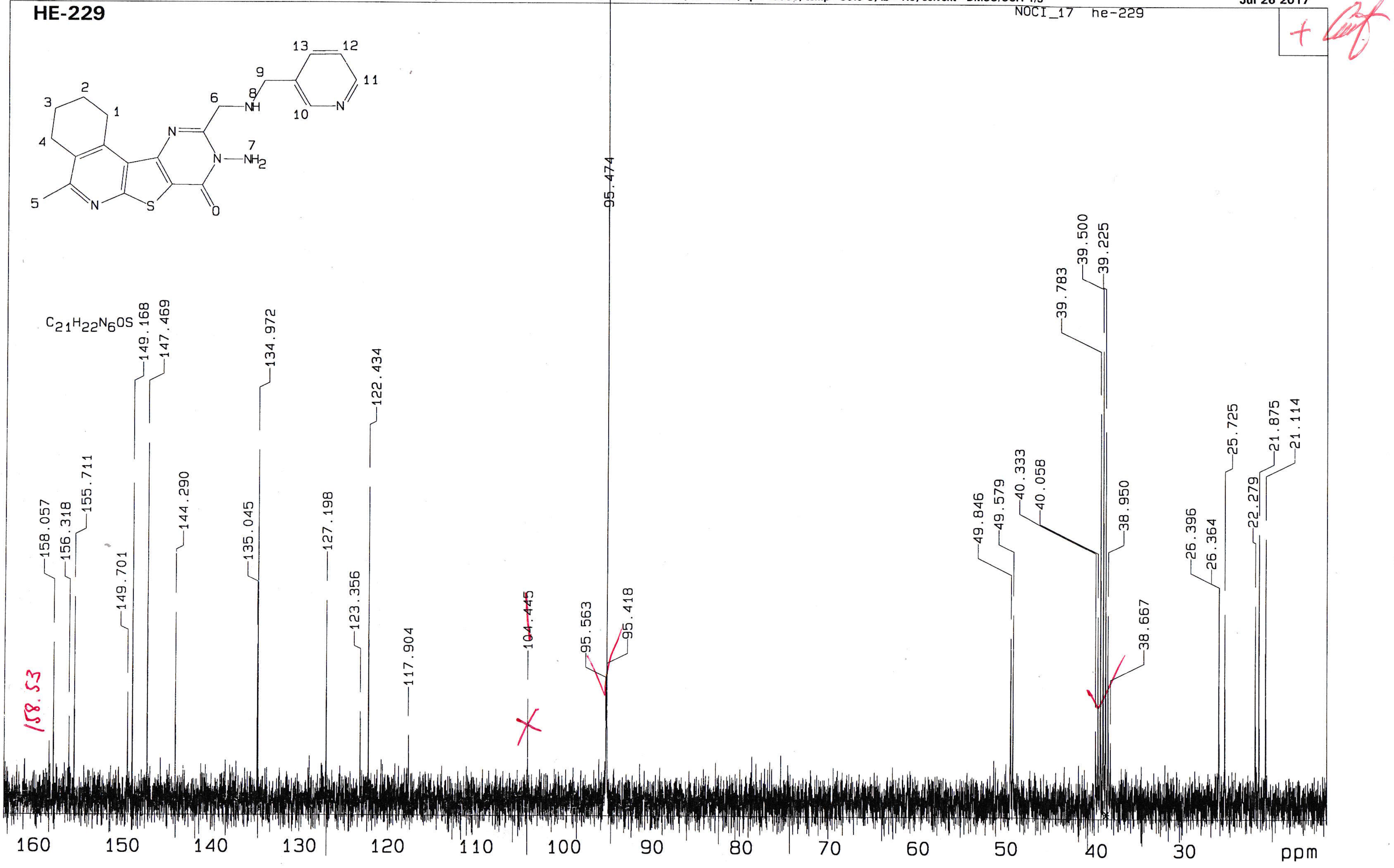

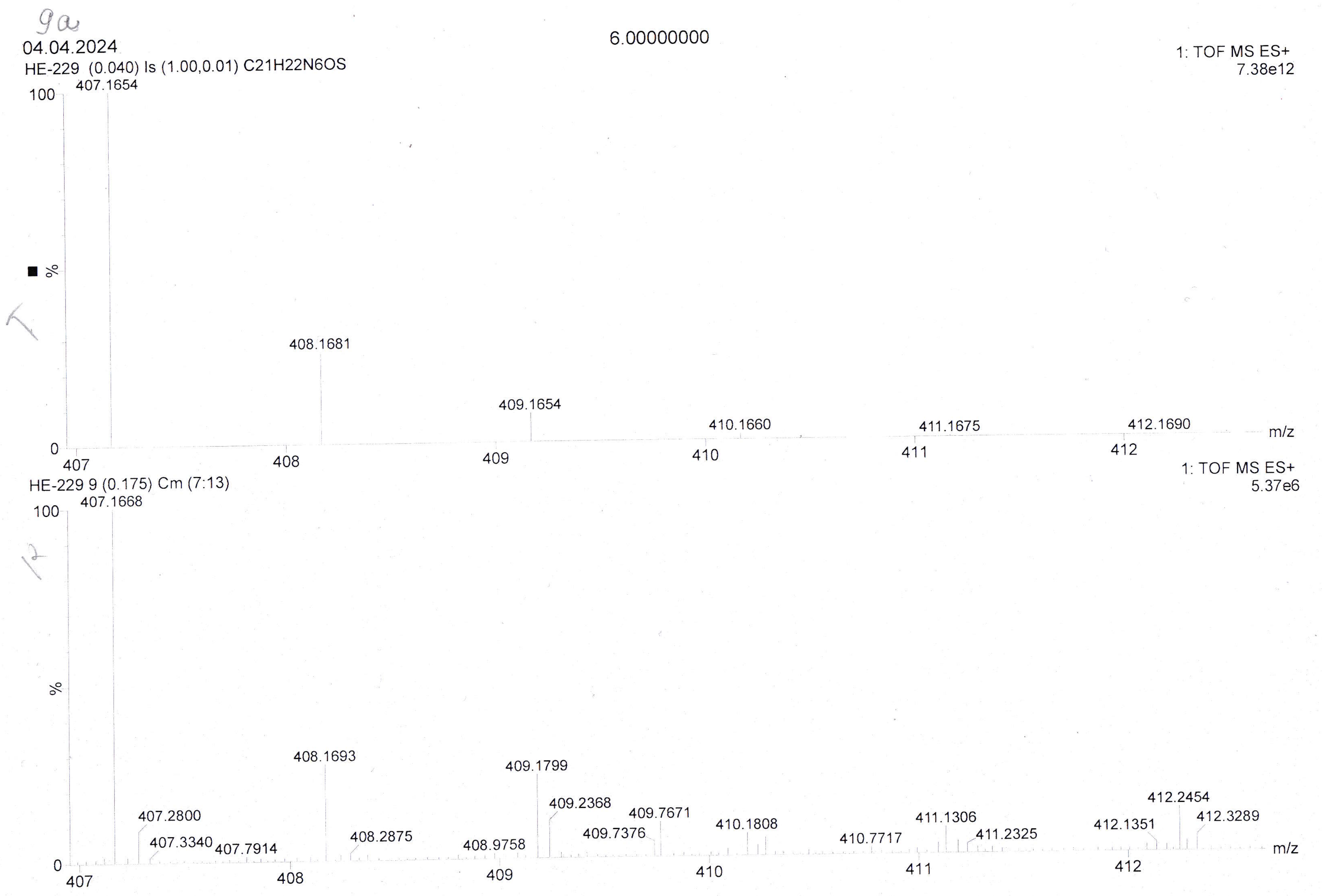
9a
04.04.2024
6.00000000
HE-229 (0.040) Is (1.00,0.01) C21H22N6OS
1: TOF MS ES+
7.38e12
407.1654
408.1681
409.1654
410.1660
411.1675
412.1690
HE-229 9 (0.175) Cm (7:13)
1: TOF MS ES+
5.37e6
407.1668
407.2800
407.3340
407.7914
408.1693
408.2875
408.9758
409.1799
409.2368
409.7376
409.7671
410.1808
410.7717
411.1306
411.2325
412.1351
412.2454
412.3289
m/z

96

Molecular Structure Research Centre, Yerevan, Armenia, Varian Mercury-300VX

H1 300.088 MHz, nt=16, np=32000, temp=30.0 C, lb=-0.2, solvent=DMSO/CCl4 1/3

Apr 2 2019

TEM-042

SAMV_19 tem-042

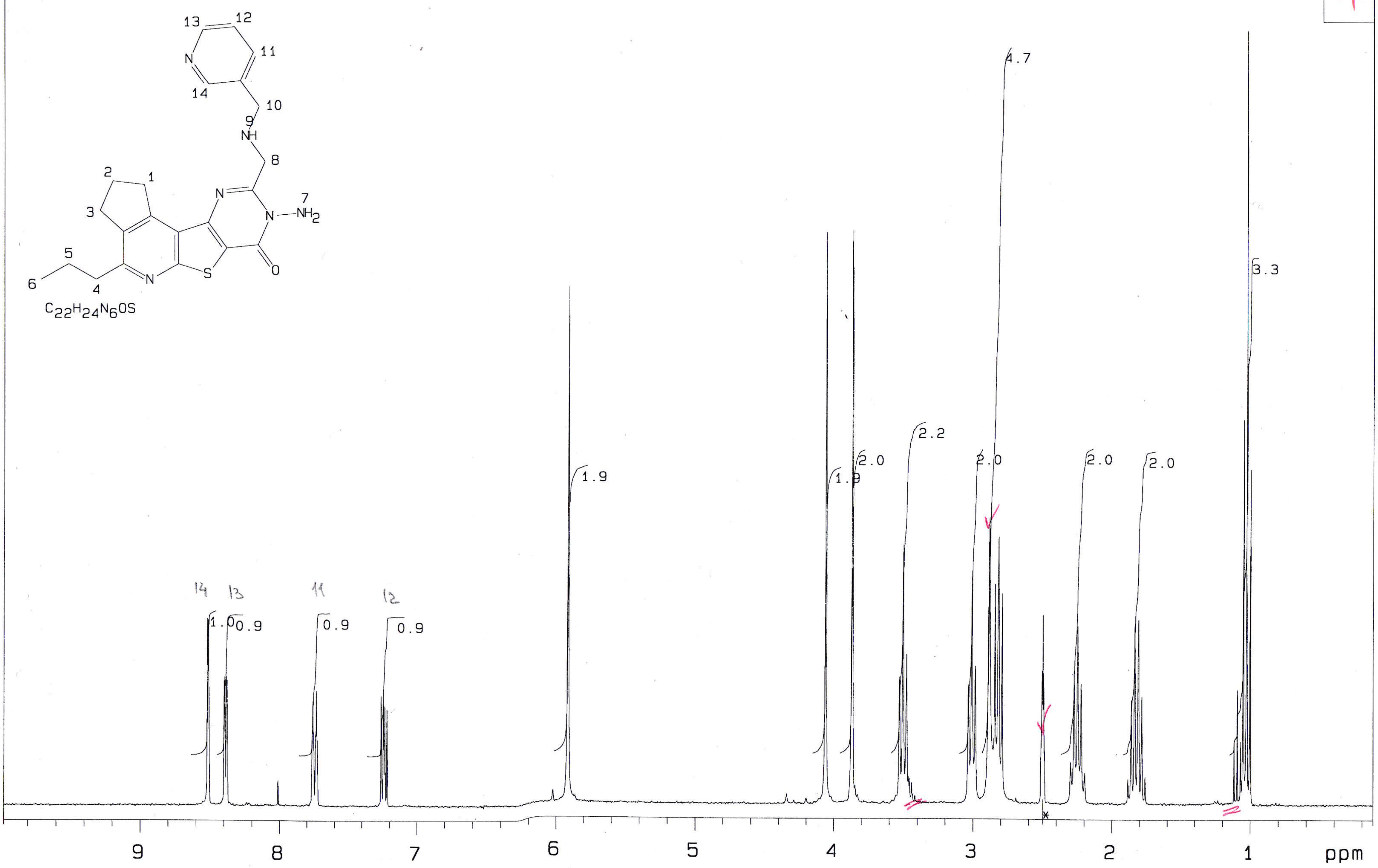

98

Molecular Structure Research Centre, Yerevan, Armenia, Varian Mercury-300VX

C13 75.465 MHz, nt = 624, np = 19998, temp = 30.0 C, lb = 1.0, solvent = DMSO/CCl4 1/3

Apr 2 2019

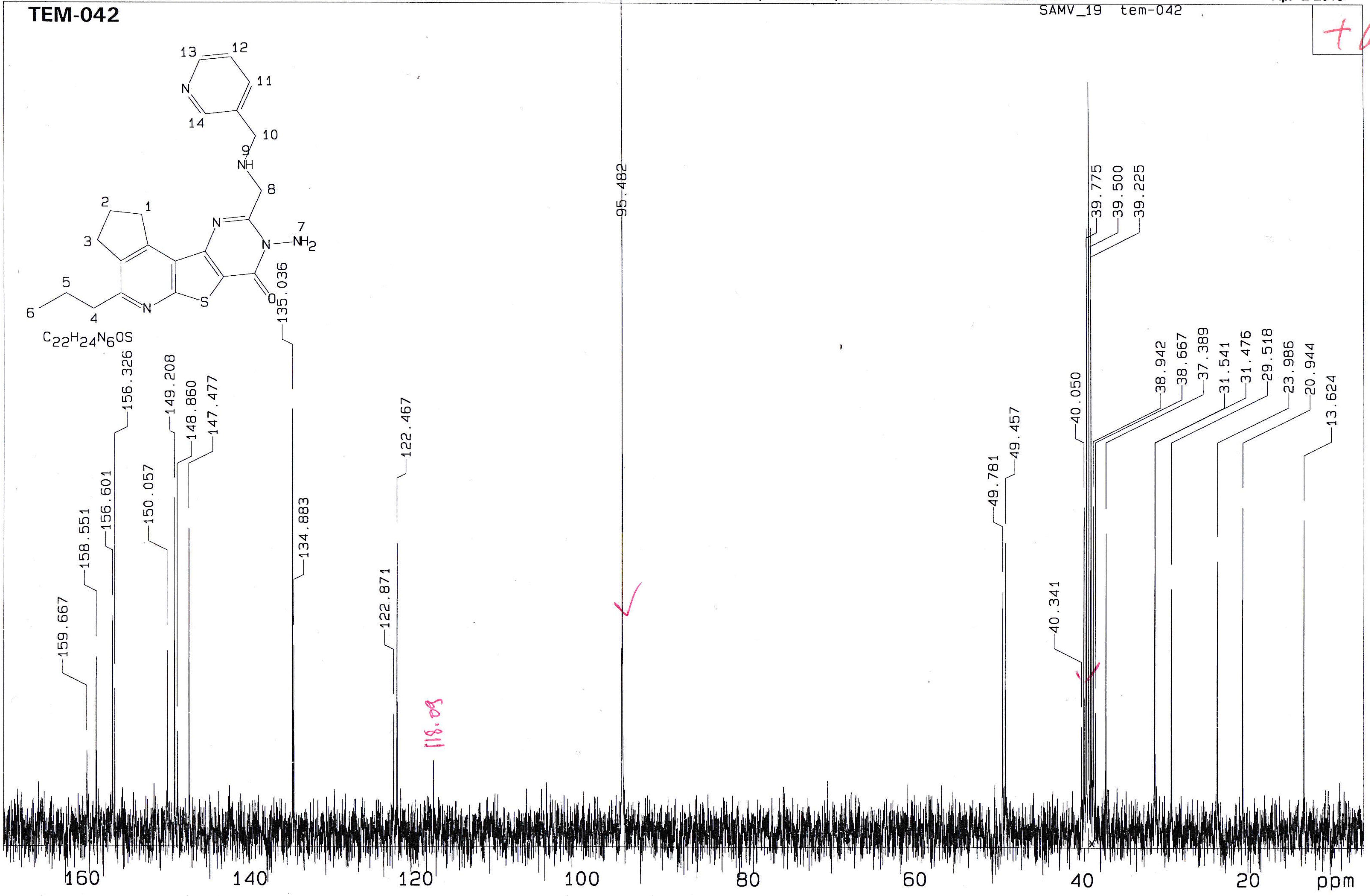

96

04.04.2024

6.00000000

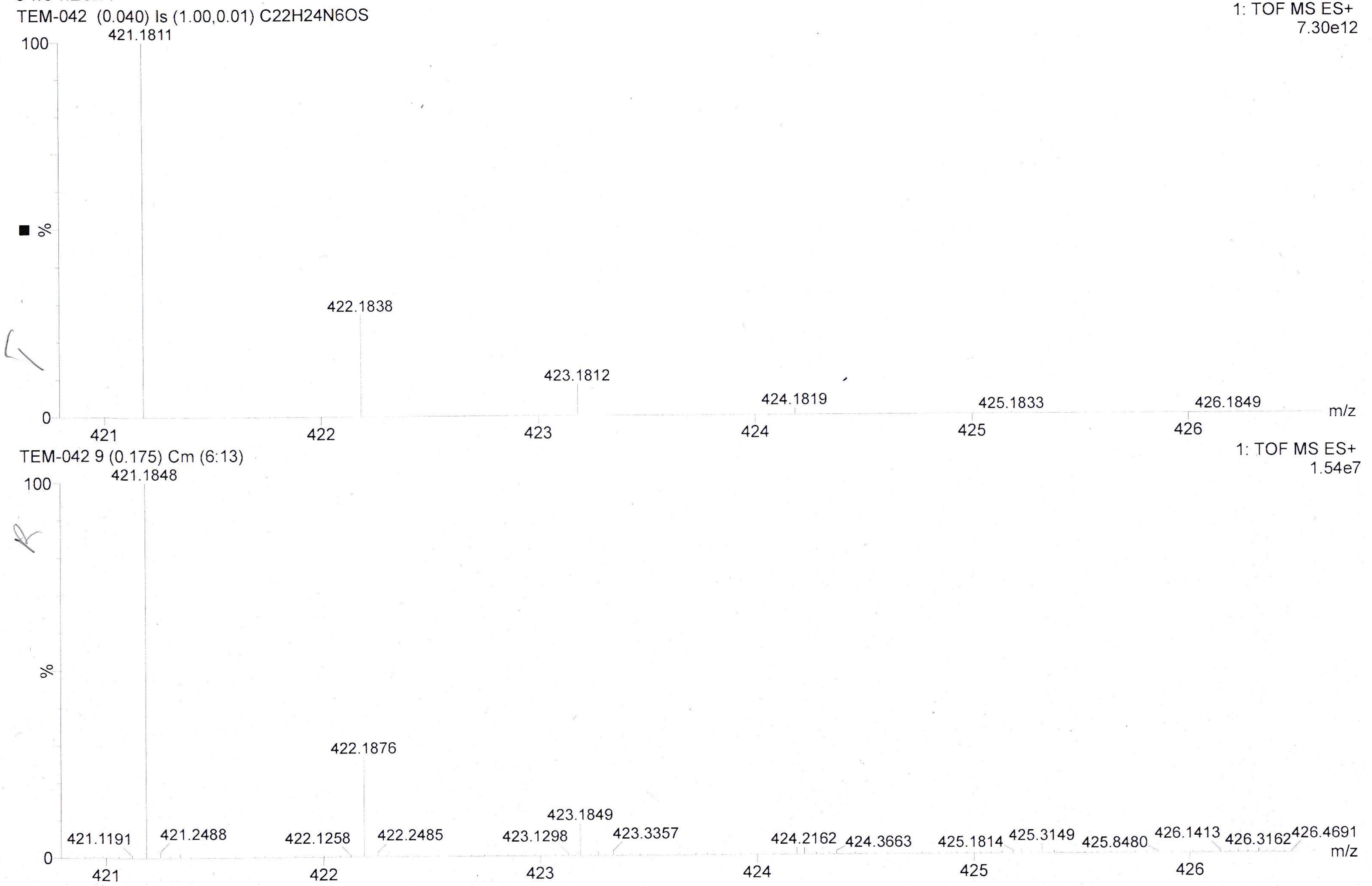

9c

Molecular Structure Research Centre, Yerevan, Armenia, Varian Mercury-300VX

H1 300.088 MHz, nt=16, np=32000, temp=30.0 C, lb=-0.2, solvent=DMSO/CCl4 1/3

Nov 29 2023

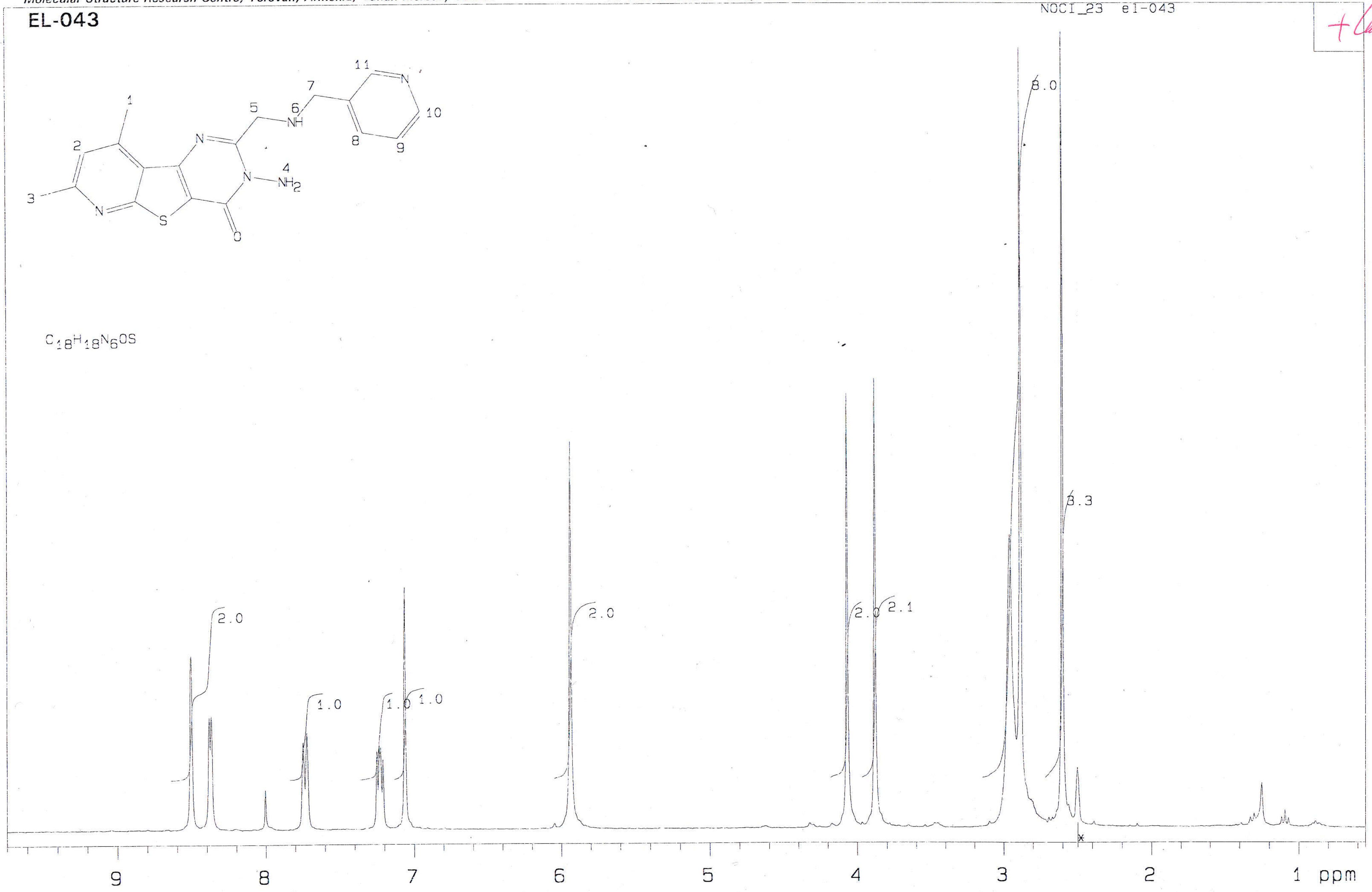

9c

Molecular Structure Research Centre, Yerevan, Armenia, Varian Mercury-300VX

C13 75.465 MHz, nt = 528, np = 19998, temp = 30.0 C, lb = 1.0, solvent = DMSO/CCl4 1/3

Nov 29 2023

NOCI_23 el-043

EL-043

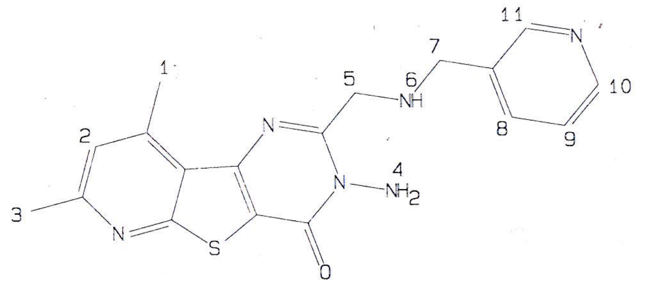

$C_{18}H_{18}N_6OS$

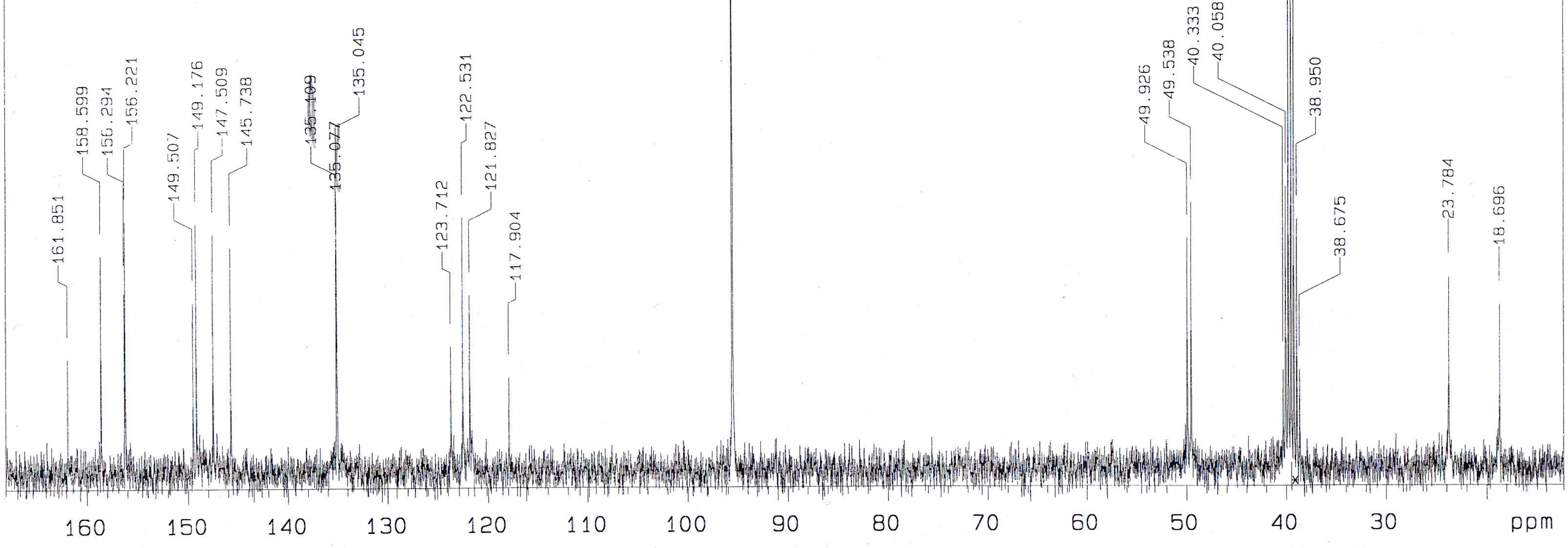

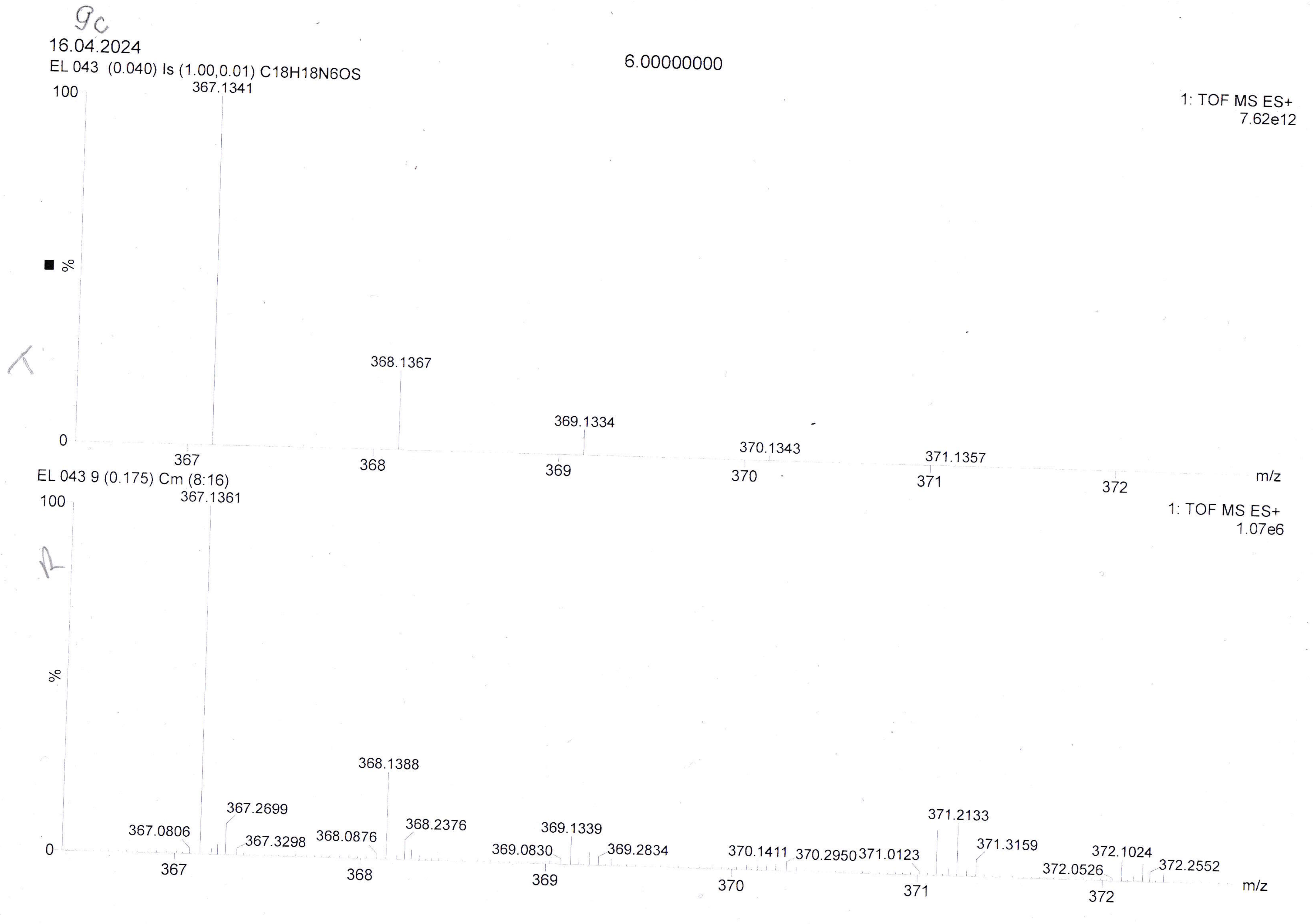
9c
16.04.2024
EL 043 (0.040) Is (1.00,0.01) C18H18N6OS
6.00000000
1: TOF MS ES+
7.62e12
T
367.1341
368.1367
369.1334
370.1343
371.1357
%
100
0
367
368
369
370
371
372
m/z
EL 043 9 (0.175) Cm (8:16)
1: TOF MS ES+
1.07e6
R
367.1361
367.0806
367.2699
367.3298
368.0876
368.1388
368.2376
369.0830
369.1339
369.2834
370.1411
370.2950
371.0123
371.2133
371.3159
372.0526
372.1024
372.2552
%
100
0
367
368
369
370
371
372
m/z

9d

Molecular Structure Research Centre, Yerevan, Armenia, Varian Mercury-300VX

H1 300.088 MHz, nt = 16, np = 32000, temp = 30.0 C, lb = -0.2, solvent = DMSO/CCl4 1/3

Mar 6 2024

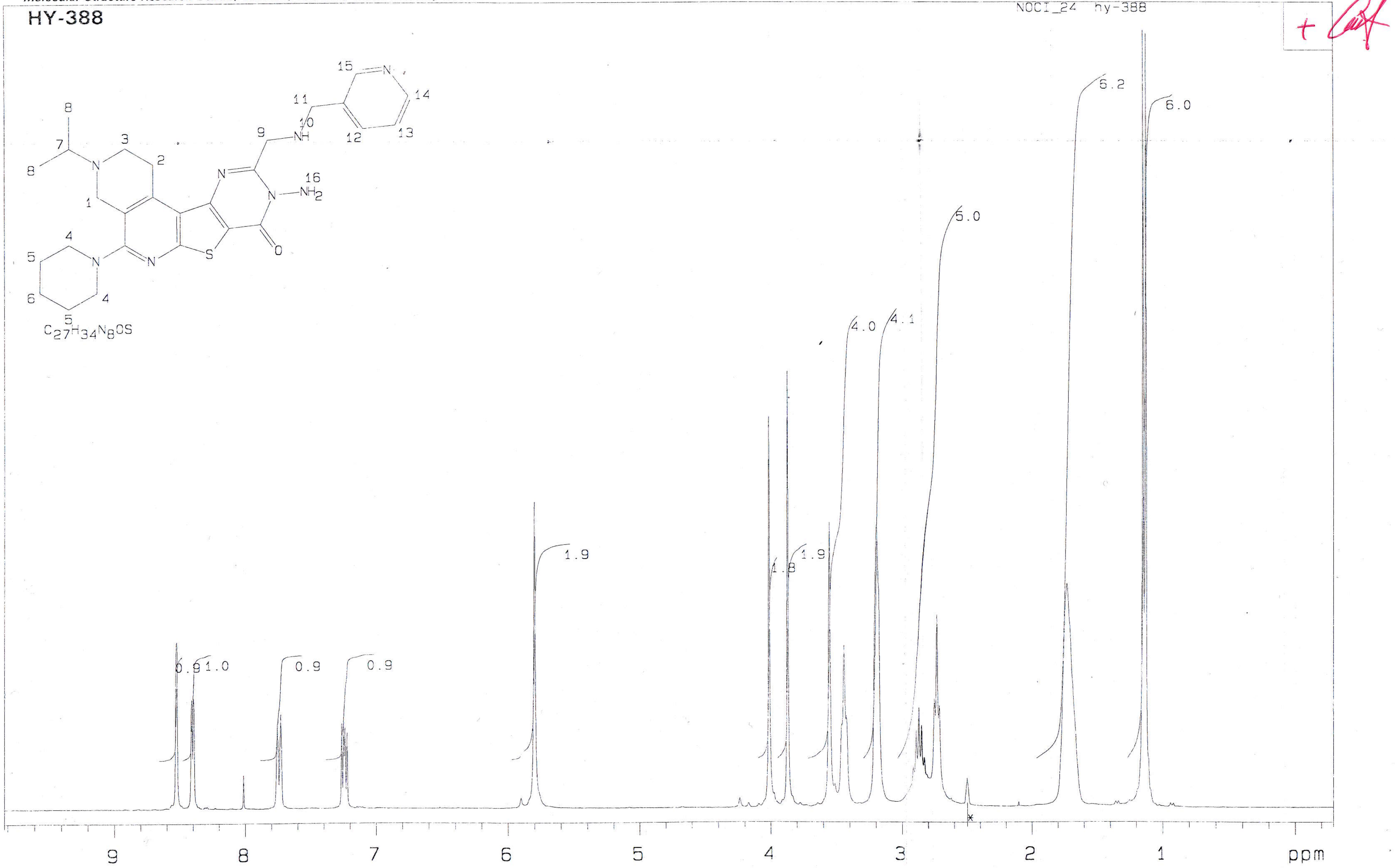

9d

Molecular Structure Research Centre, Yerevan, Armenia, Varian Mercury-300VX

C13 75.465 MHz, nt = 2272, np = 19998, temp = 30.0 C, lb = 1.0, solvent = DMSO/CCl4 1/3

Mar 6 2024

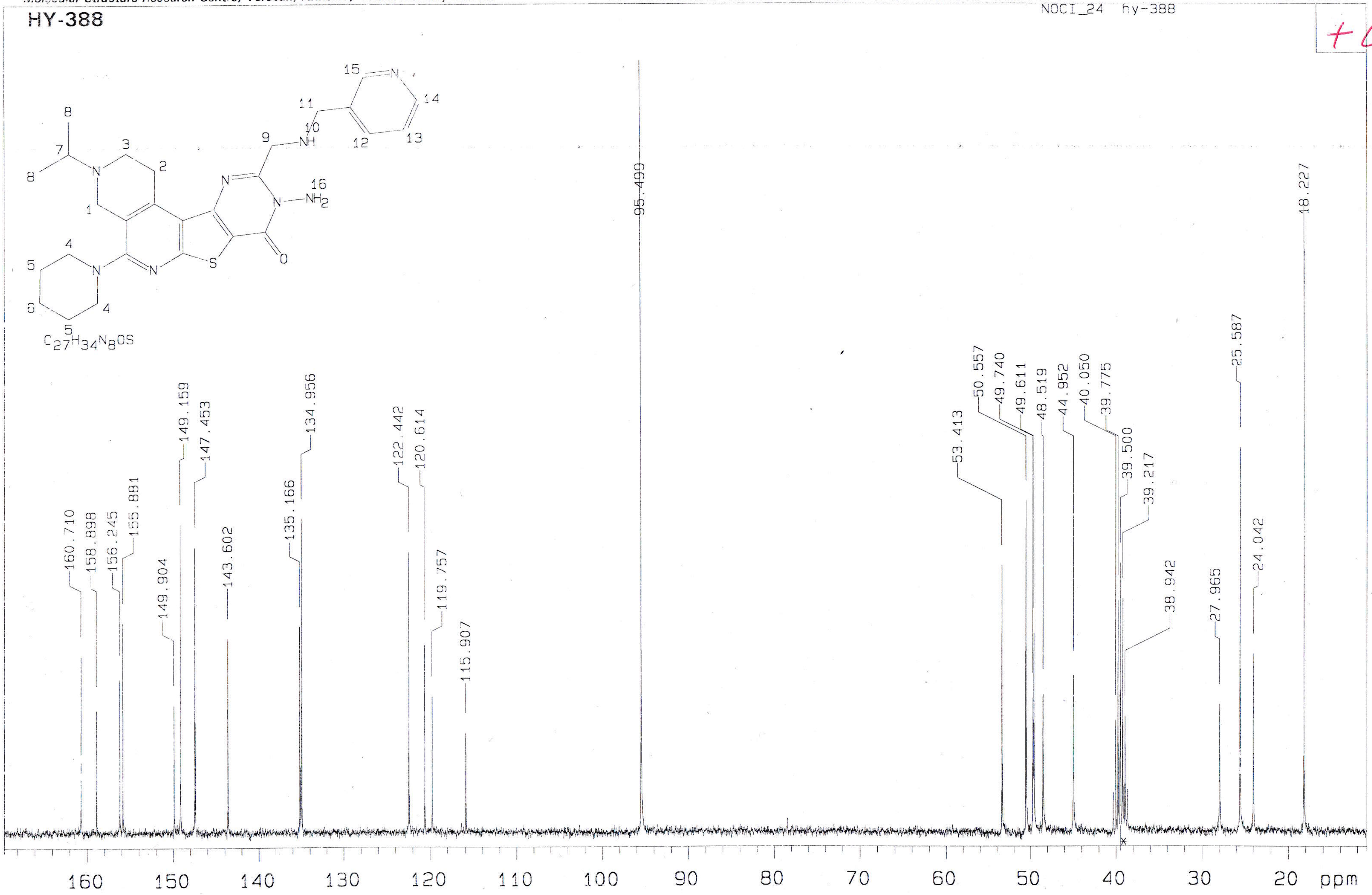

+ [illegible]

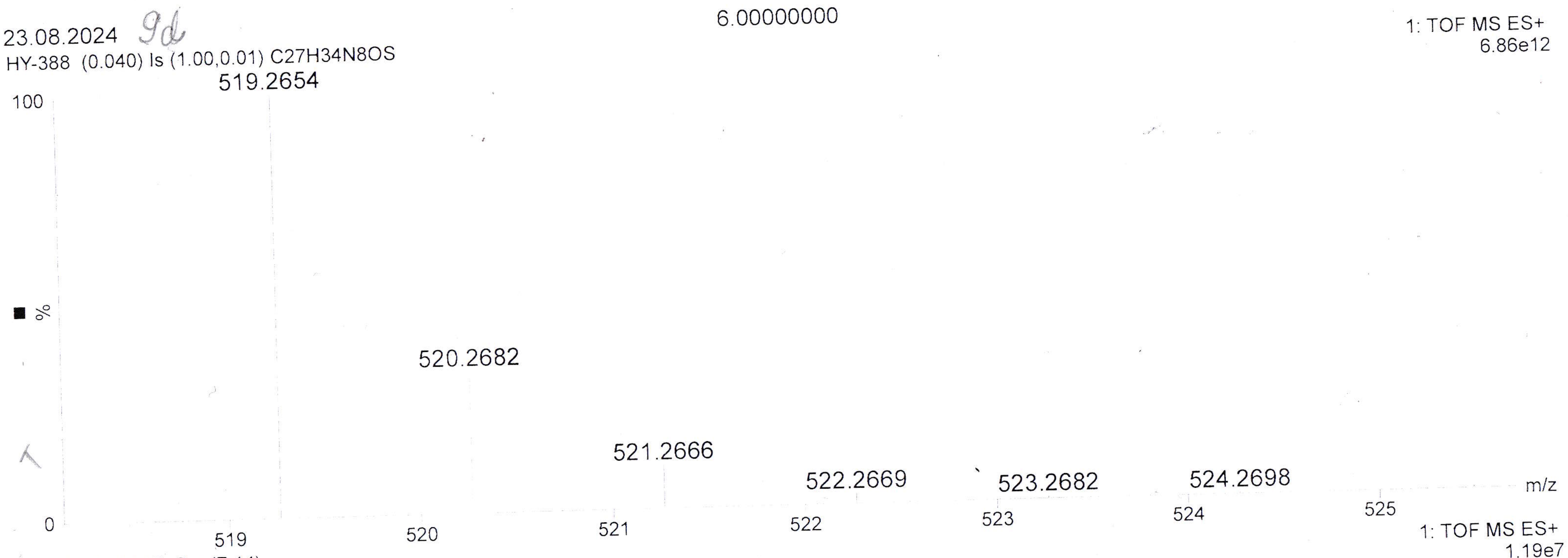

23.08.2024 9d
6.00000000
1: TOF MS ES+
6.86e12
HY-388 (0.040) Is (1.00,0.01) C27H34N8OS
519.2654
100
%
T
520.2682
521.2666
522.2669
523.2682
524.2698
m/z
0
519
520
521
522
523
524
525

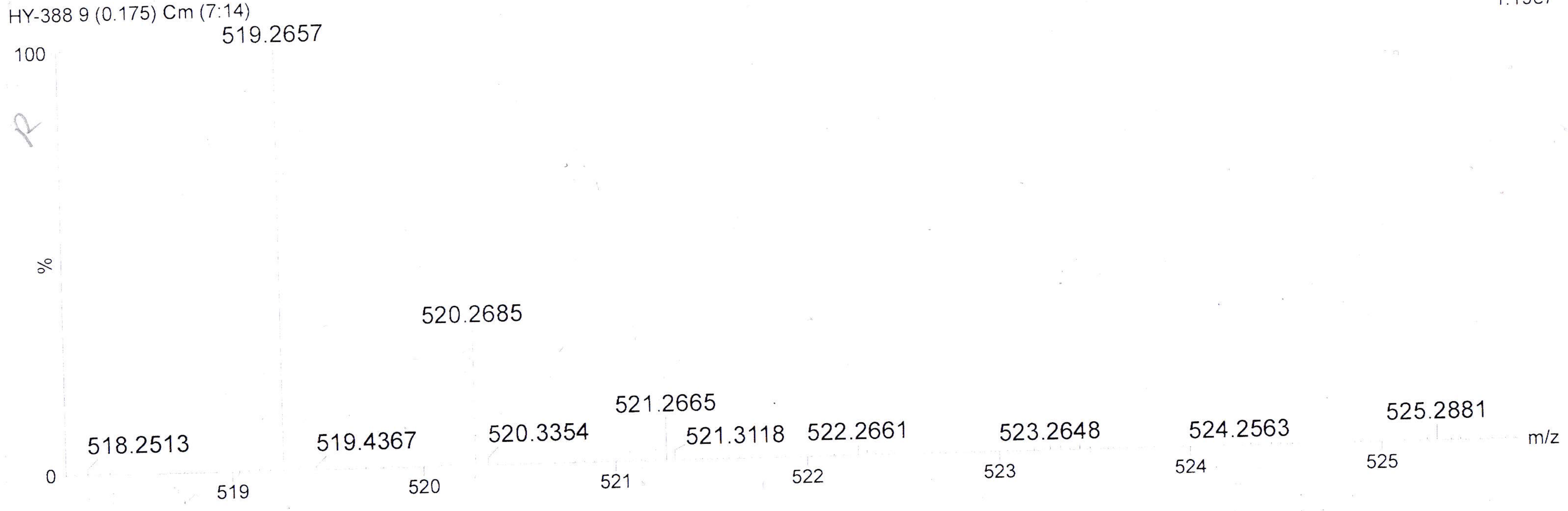

1: TOF MS ES+
1.19e7
HY-388 9 (0.175) Cm (7:14)
519.2657
100
R
%
520.2685
521.2665
518.2513
519.4367
520.3354
521.3118
522.2661
523.2648
524.2563
525.2881
m/z
0
519
520
521
522
523
524
525

10a

Molecular Structure Research Centre, Yerevan, Armenia, Varian Mercury-300VX

H1 300.088 MHz, nt=16, np=32000, temp=30.0 C, lb=-0.2, solvent=DMSO/CCl4 1/3

Aug 11 2017

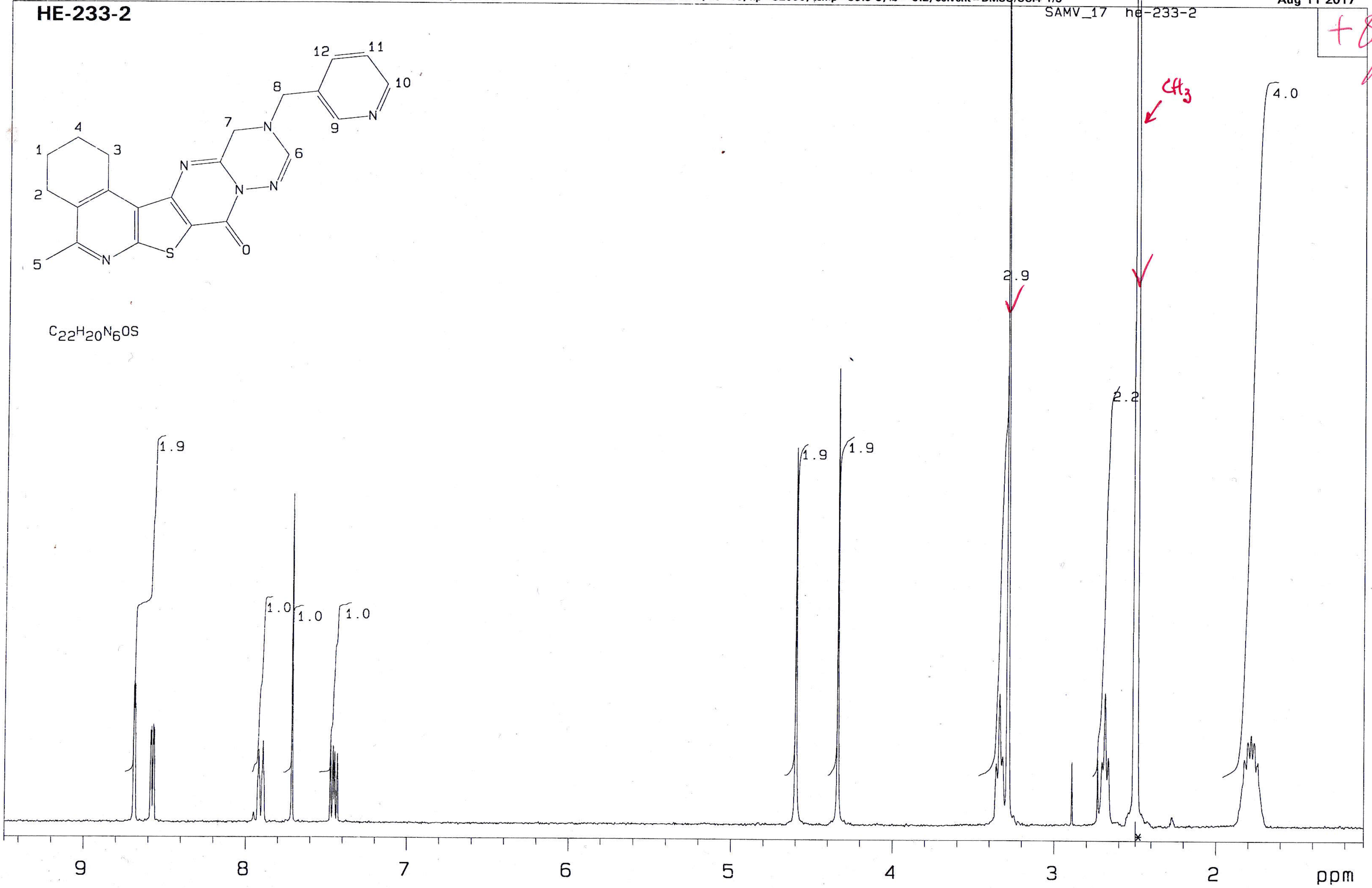

10a

30.01.2024

6.00000000

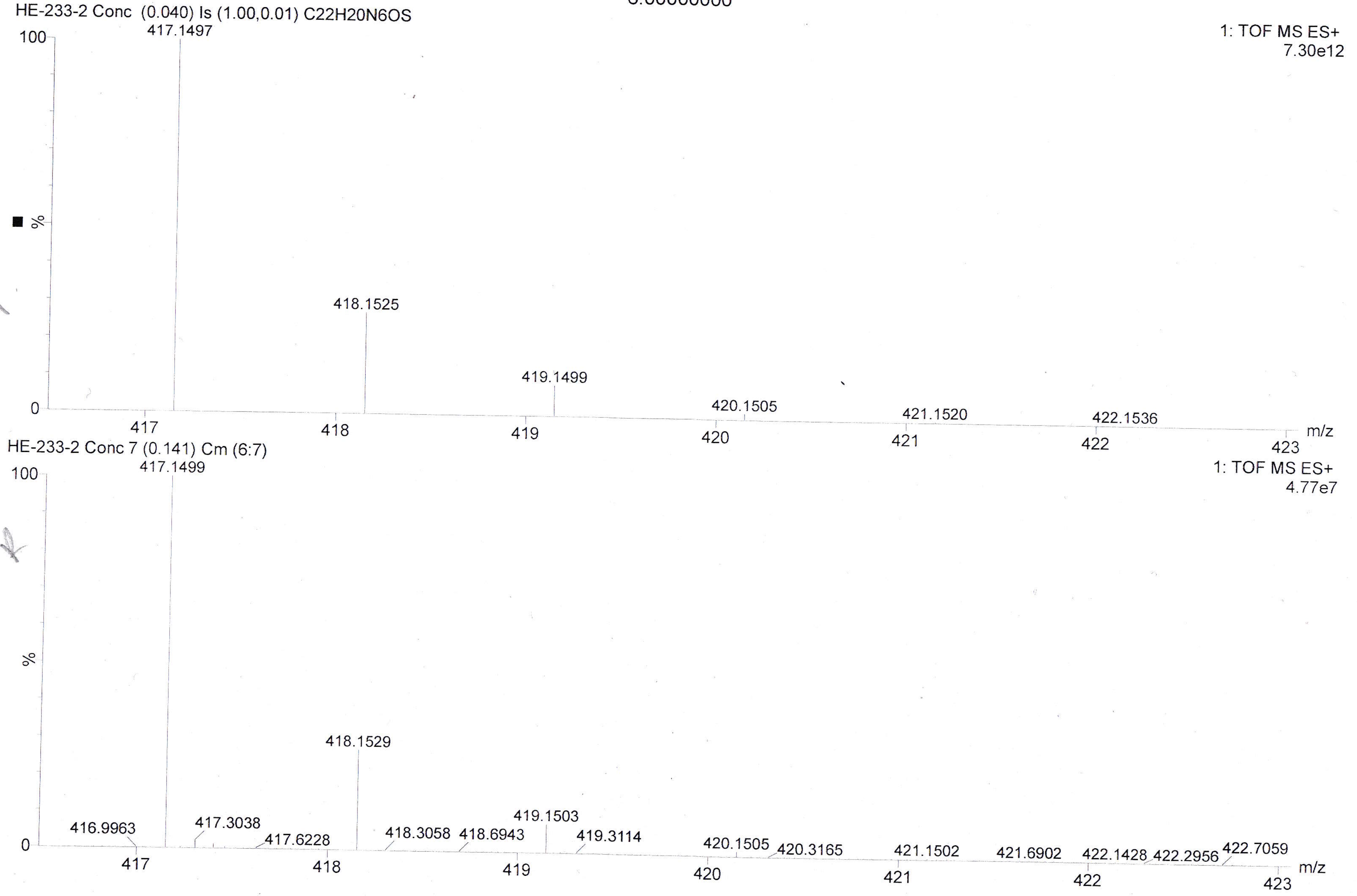

108

Molecular Structure Research Centre, Yerevan, Armenia, Varian Mercury-300VX — H1 300.088 MHz, nt=16, np=32000, temp=30.0 C, lb=-0.2, solvent=DMSO/CCl4 1/3 — Apr 8 2019

TEM-049

NOCI_19 tem-049

$C_{23}H_{22}N_6OS$

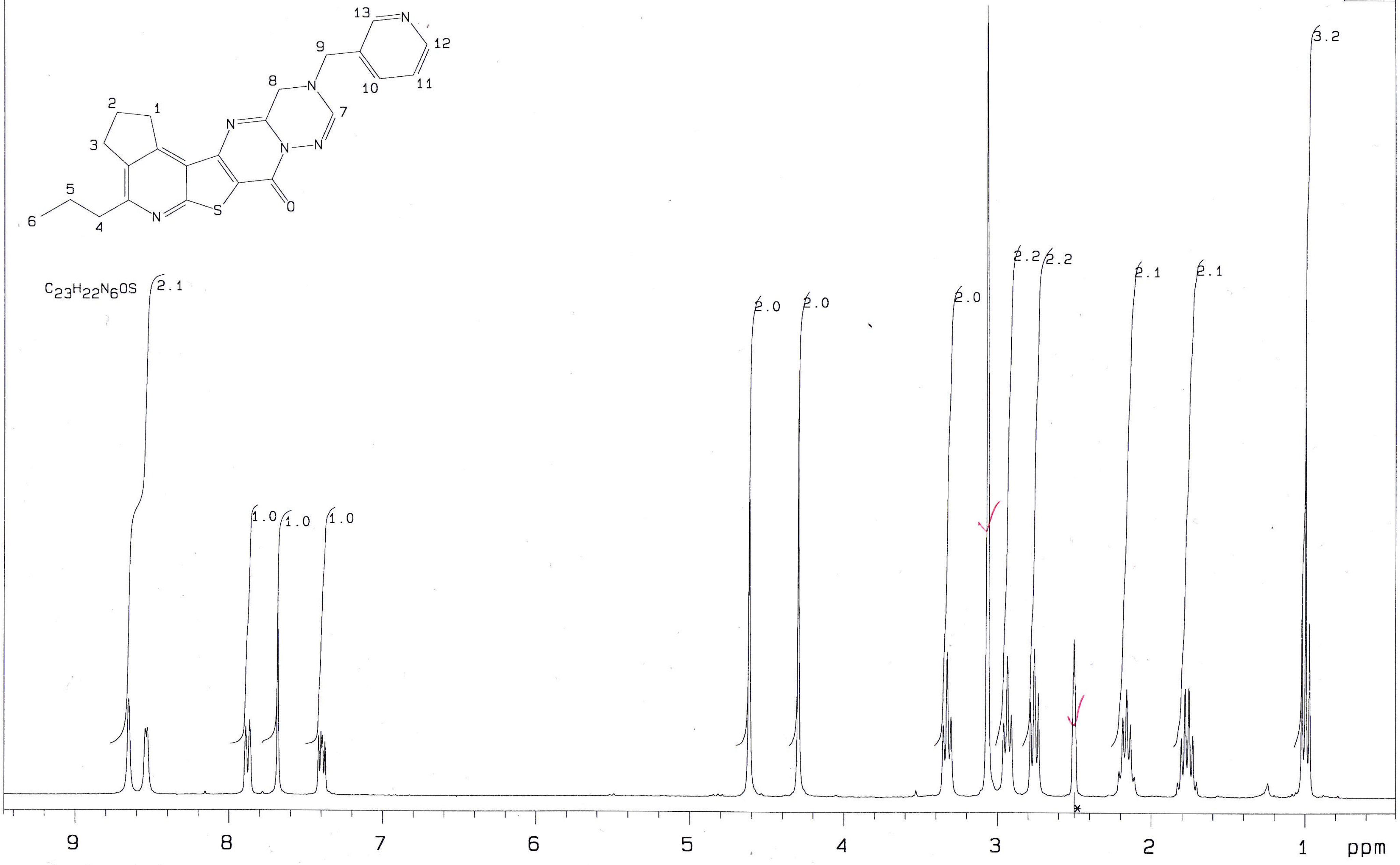

Molecular Structure Research Centre, Yerevan, Armenia, Varian Mercury-300VX

C13 75.465 MHz, nt=560, np=19998, temp=30.0 C, lb=1.0, solvent=DMSO/CCl4 1/3

Apr 8 2019

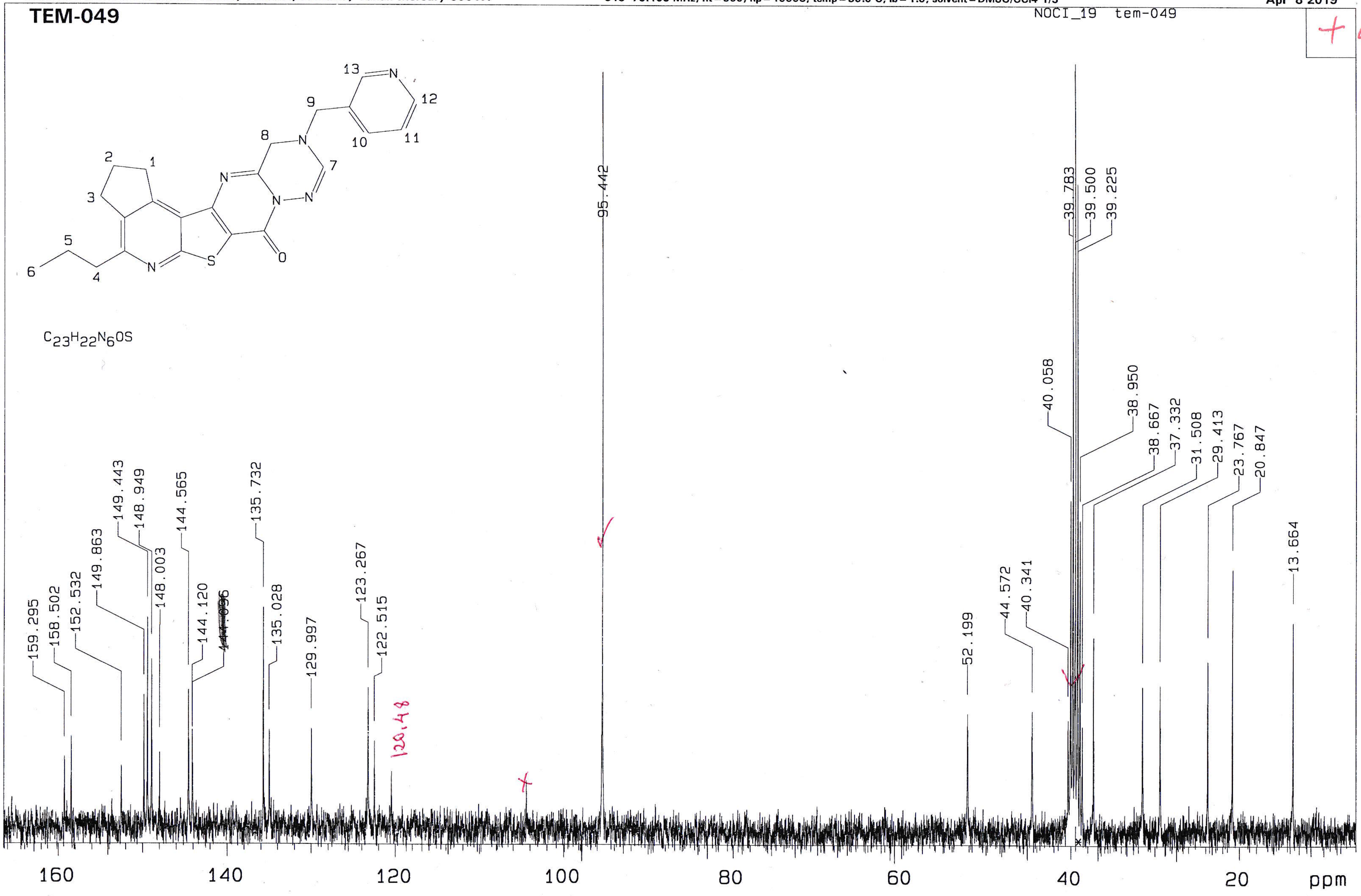

108

18.12.2023

TEM-049 (0.040) Is (1.00,1.00) C23H22N6OS

Theoritical

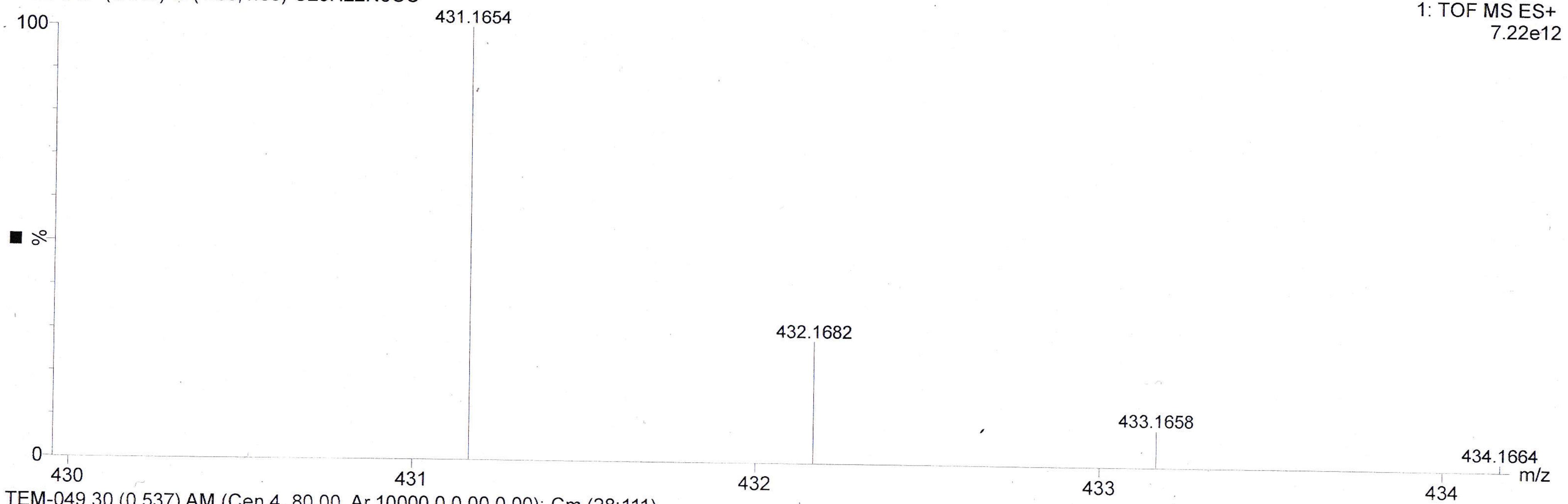

TEM-049 30 (0.537) AM (Cen,4, 80.00, Ar,10000.0,0.00,0.00); Cm (28:111)

Real

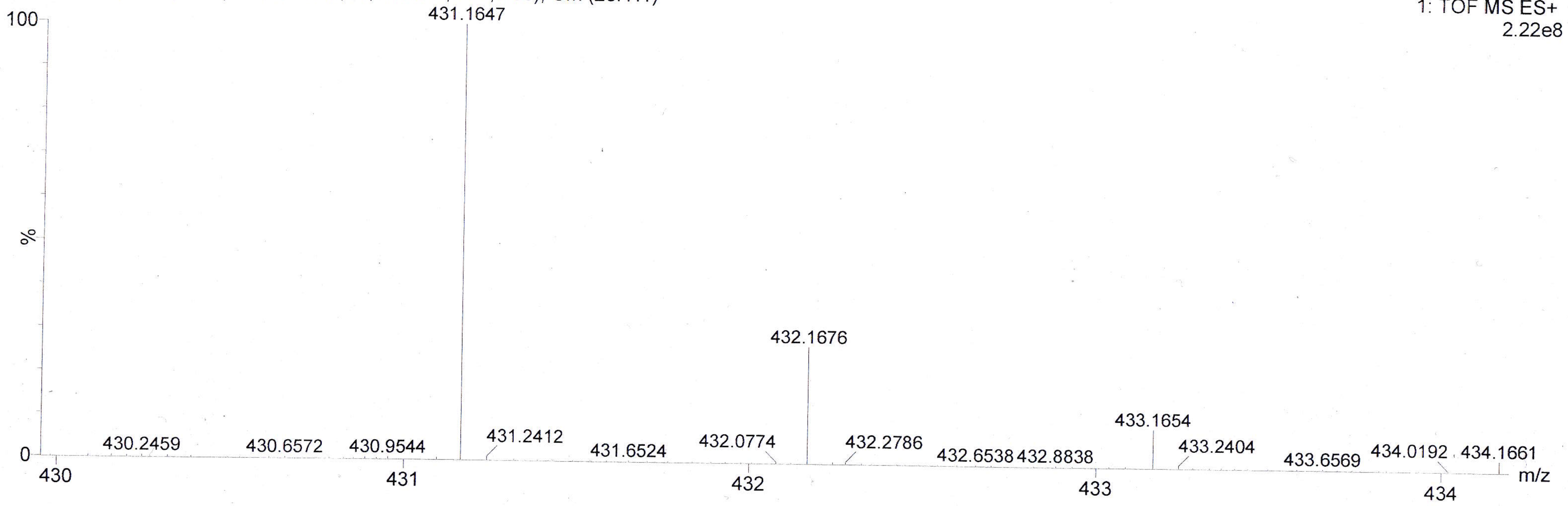

10c

Molecular Structure Research Centre, Yerevan, Armenia, Varian Mercury-300VX

H1 300.088 MHz, nt = 16, np = 32000, temp = 30.0 C, lb = -0.2, solvent = DMSO

Jan 17 2024

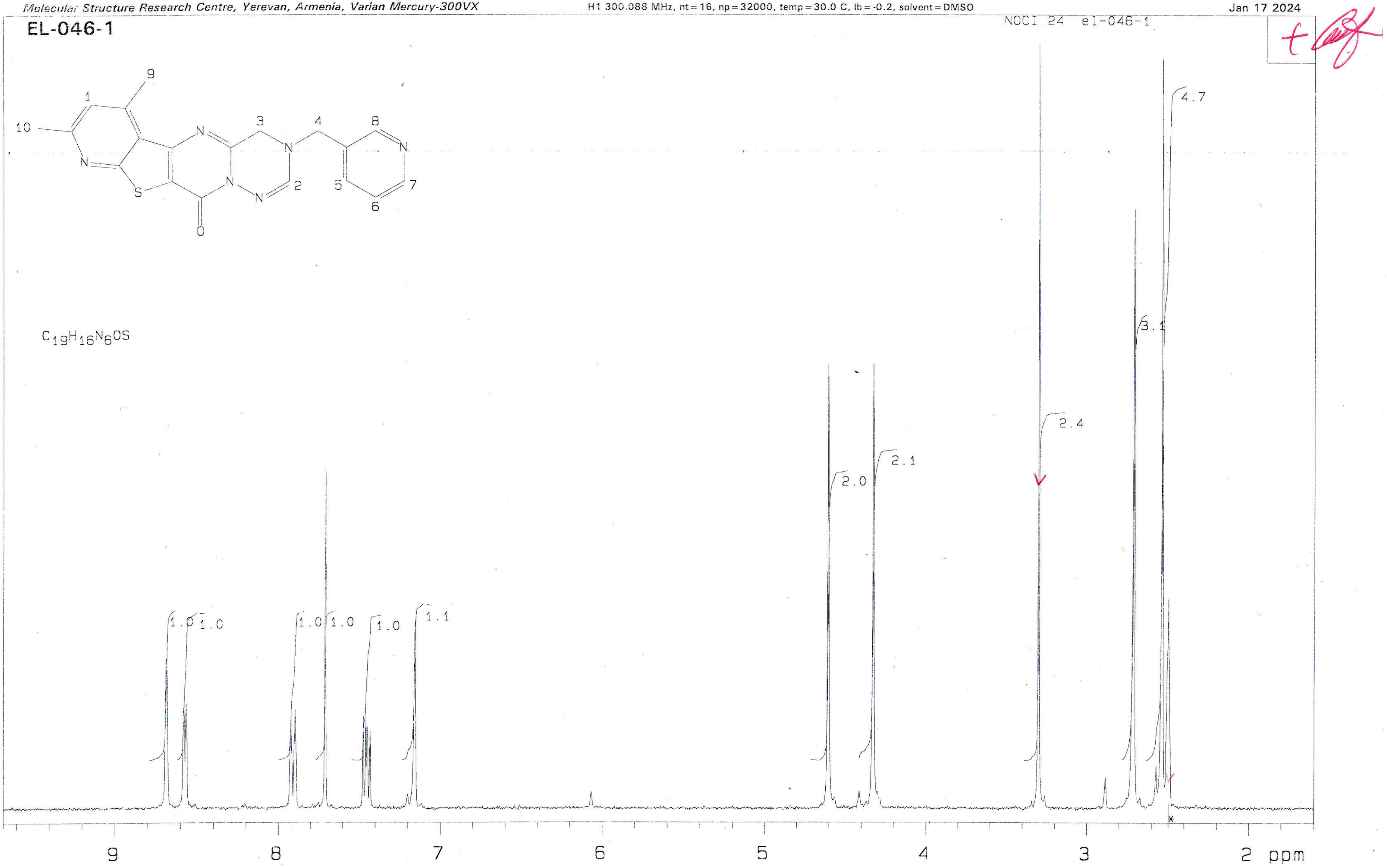

10c

Molecular Structure Research Centre, Yerevan, Armenia. Varian Mercury-300VX C13 75.465 MHz, nt=608, np=19998, temp=30.0 C, lb=1.0, solvent=DMSO Jan 16 2024

EL-046

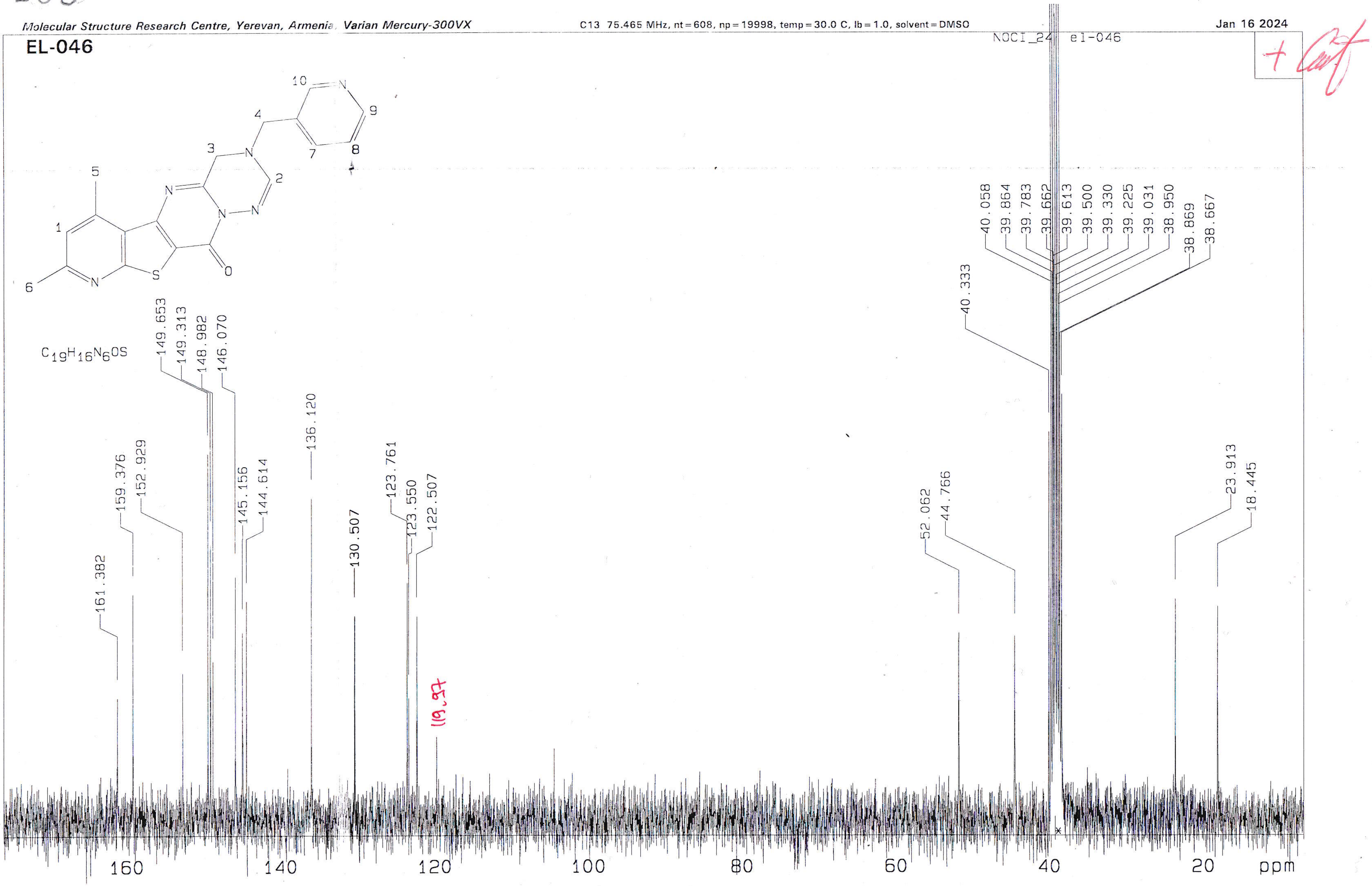

10c

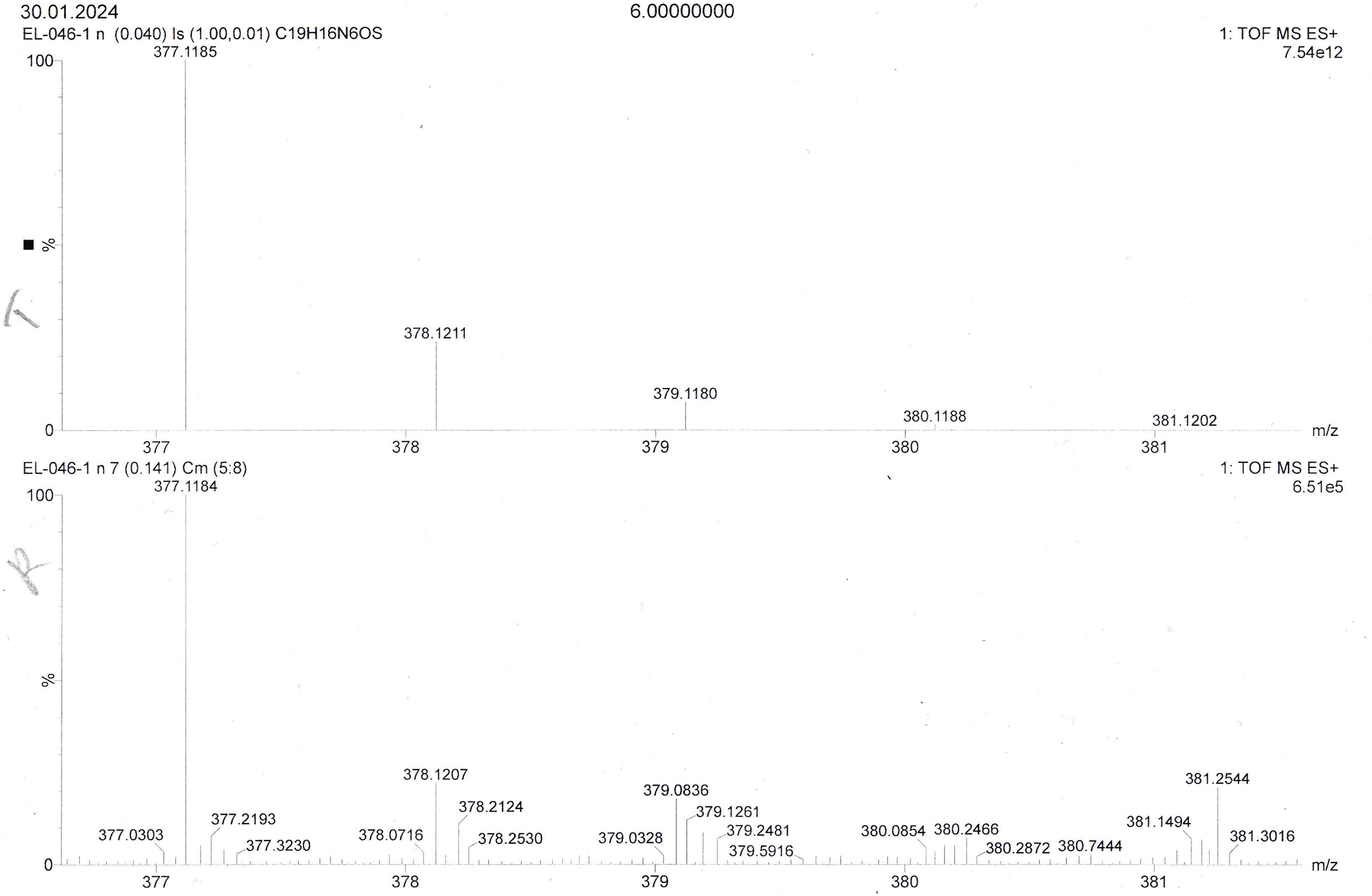
30.01.2024
6.00000000
EL-046-1 n (0.040) Is (1.00,0.01) C19H16N6OS
1: TOF MS ES+
7.54e12
100
%
0
377.1185
378.1211
379.1180
380.1188
381.1202
377
378
379
380
381
m/z
EL-046-1 n 7 (0.141) Cm (5:8)
1: TOF MS ES+
6.51e5
100
%
0
377.1184
377.0303
377.2193
377.3230
378.0716
378.1207
378.2124
378.2530
379.0328
379.0836
379.1261
379.2481
379.5916
380.0854
380.2466
380.2872
380.7444
381.1494
381.2544
381.3016
377
378
379
380
381
m/z

10d

Molecular Structure Research Centre, Yerevan, Armenia, Varian Mercury-300VX

H1 300.086 MHz, nt = 16, np = 32000, temp = 30.0 C, lb = -0.2, solvent = CDCL3

Jul 24 2024

NOCI_24 hy-391-2

HY-391-2

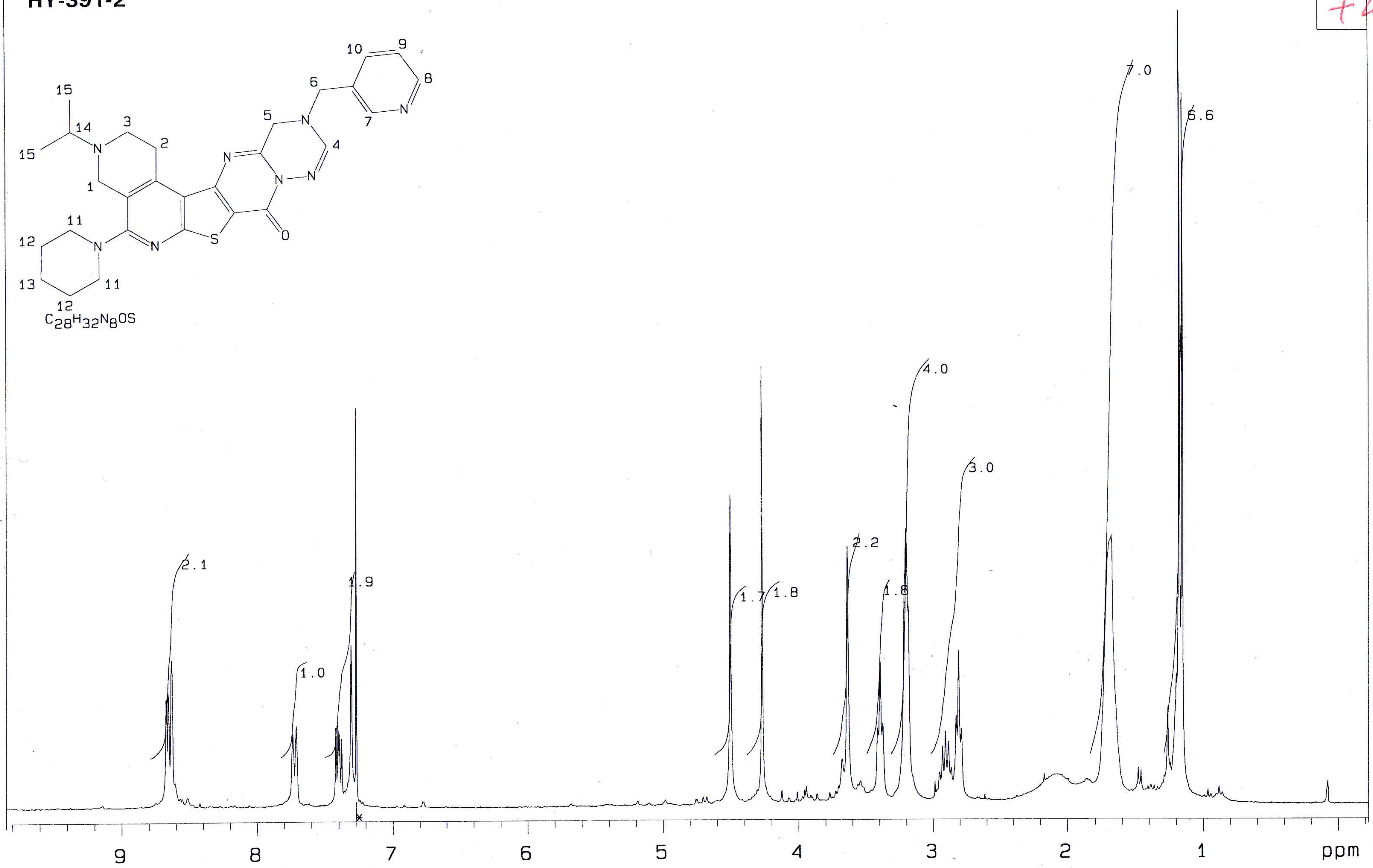

10d

Molecular Structure Research Centre, Yerevan, Armenia, Varian Mercury-300VX

C13 75.464 MHz, nt=1312, np=32000, temp=30.0 C, lb=1.0, solvent=CDCL3

Jul 24 2024

NOCI_24 hy-391-2

HY-391-2

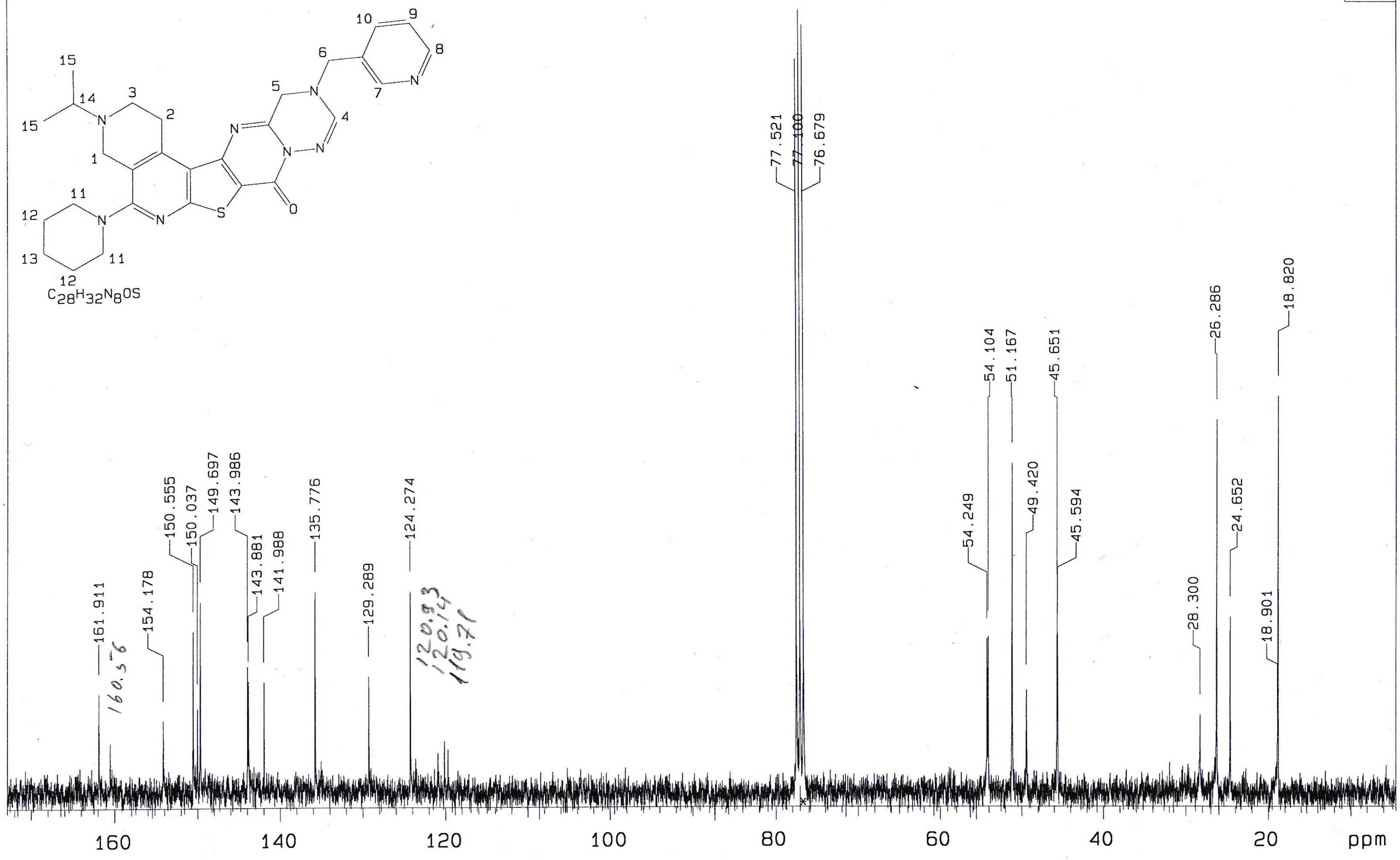

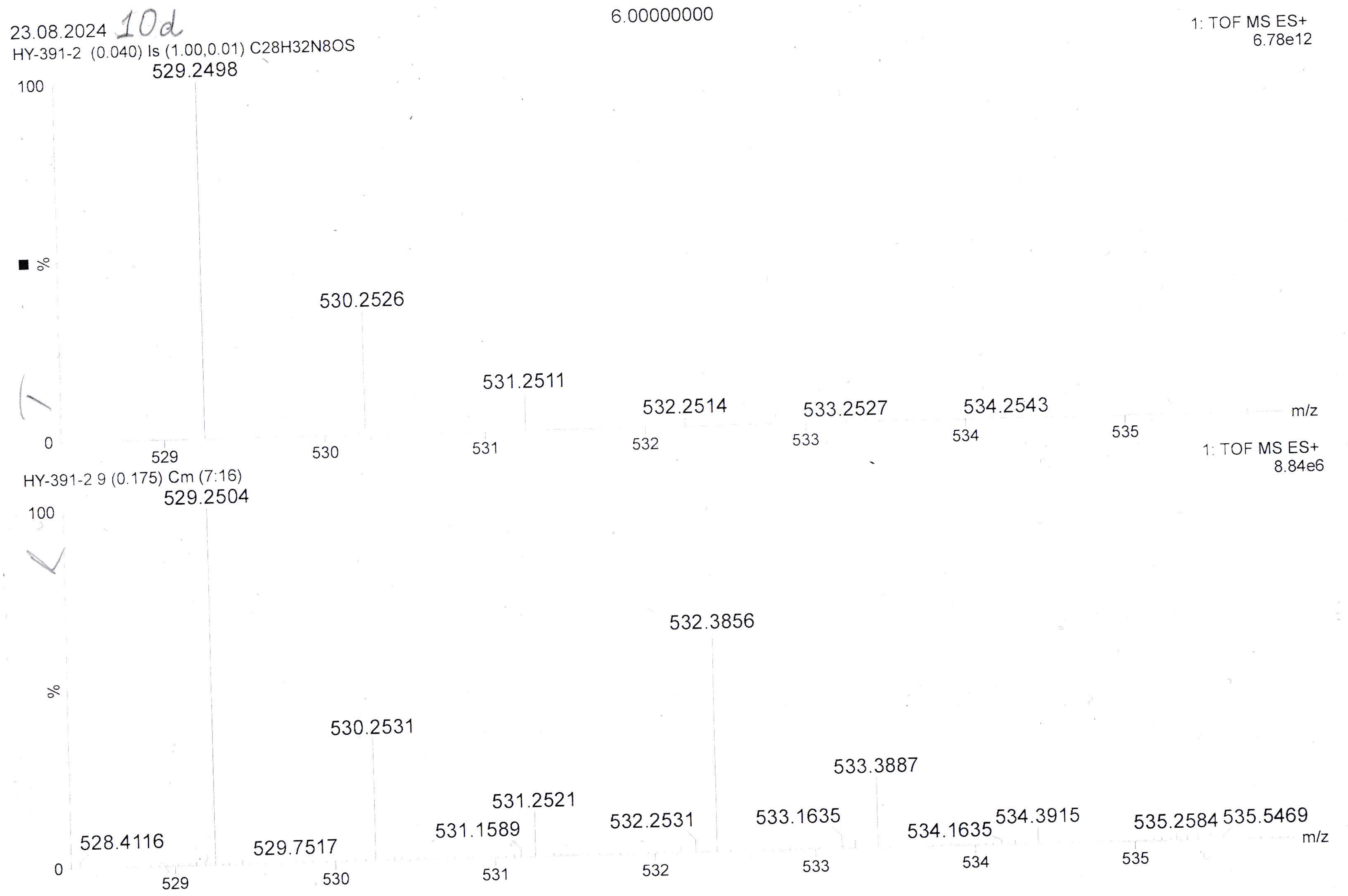
23.08.2024 10d
HY-391-2 (0.040) Is (1.00,0.01) C28H32N8OS
6.00000000
1: TOF MS ES+
6.78e12
100
%
0
529.2498
530.2526
531.2511
532.2514
533.2527
534.2543
529
530
531
532
533
534
535
m/z
HY-391-2 9 (0.175) Cm (7:16)
1: TOF MS ES+
8.84e6
100
%
0
529.2504
532.3856
530.2531
533.3887
531.2521
528.4116
529.7517
531.1589
532.2531
533.1635
534.1635
534.3915
535.2584
535.5469
529
530
531
532
533
534
535
m/z
